# Endothelial cell-derived GABA signaling modulates neuronal migration and postnatal behavior

**DOI:** 10.1038/cr.2017.135

**Published:** 2017-10-31

**Authors:** Suyan Li, Peeyush Kumar T, Sampada Joshee, Timo Kirschstein, Sivan Subburaju, Jahan S Khalili, Jonas Kloepper, Chuang Du, Abdallah Elkhal, Gábor Szabó, Rakesh K Jain, Rüdiger Köhling, Anju Vasudevan

**Affiliations:** 1 Department of Psychiatry, Harvard Medical School, Boston, MA 02215, USA;; 2 Angiogenesis and Brain Development Laboratory, Division of Basic Neuroscience, McLean Hospital, 115 Mill Street, Belmont, MA 02478, USA;; 3 Oscar-Langendorff-Institute of Physiology, Rostock University Medical Center, Gertrudenstrasse 9, 18057 Rostock, Germany;; 4 Program in Structural and Molecular Neuroscience, McLean Hospital, 115 Mill Street, Belmont, MA 02478, USA;; 5 Personal Peptides LLC, Houston, TX 77002, USA;; 6 Edwin L. Steele Laboratories, Department of Radiation Oncology, Massachusetts General Hospital and Harvard Medical School, Boston, MA 02114, USA;; 7 Department of Neuroscience, Tufts University School of Medicine, Boston, MA 02148, USA;; 8 Department of Surgery, Harvard Medical School, Boston, MA 02115, USA;; 9 Division of Transplantation, Brigham and Women's Hospital, 221 Longwood Avenue, EBRC 309, Boston, MA 02115, USA;; 10 Laboratory of Molecular Biology and Genetics, Department of Gene Technology and Developmental Neurobiology, Institute of Experimental Medicine, 1083 Budapest, Hungary

**Keywords:** blood vessel, brain, angiogenesis, neuronal migration, GABA signaling, GABAergic neurons, endothelial cells

## Abstract

The cerebral cortex is essential for integration and processing of information that is required for most behaviors. The exquisitely precise laminar organization of the cerebral cortex arises during embryonic development when neurons migrate successively from ventricular zones to coalesce into specific cortical layers. While radial glia act as guide rails for projection neuron migration, pre-formed vascular networks provide support and guidance cues for GABAergic interneuron migration. This study provides novel conceptual and mechanistic insights into this paradigm of vascular-neuronal interactions, revealing new mechanisms of GABA and its receptor-mediated signaling via embryonic forebrain endothelial cells. With the use of two new endothelial cell specific conditional mouse models of the GABA pathway (*Gabrb3^ΔTie2-Cre^* and *Vgat^ΔTie2-Cre^*), we show that partial or complete loss of GABA release from endothelial cells during embryogenesis results in vascular defects and impairs long-distance migration and positioning of cortical interneurons. The downstream effects of perturbed endothelial cell-derived GABA signaling are critical, leading to lasting changes to cortical circuits and persistent behavioral deficits. Furthermore, we illustrate new mechanisms of activation of GABA signaling in forebrain endothelial cells that promotes their migration, angiogenesis and acquisition of blood-brain barrier properties. Our findings uncover and elucidate a novel endothelial GABA signaling pathway in the CNS that is distinct from the classical neuronal GABA signaling pathway and shed new light on the etiology and pathophysiology of neuropsychiatric diseases, such as autism spectrum disorders, epilepsy, anxiety, depression and schizophrenia.

## Introduction

Today, one in four people worldwide suffer every year from some form of neuropsychiatric illness. Drugs used in psychiatry usually act to ease symptoms with no cure, due to lack of mechanistic insights into how these diseases initiate. One factor known to exert extremely broad influence on brain development and network formation is gamma-aminobutyric acid (GABA). Abnormalities in GABAergic neurons and defects in cortical inhibition are implicated underlying the etiology of autism spectrum disorders (ASD), epilepsy, schizophrenia, anxiety and depression^1,2,3,4,5,6^. Given the significance of abnormal early brain development that leads to these serious neuropsychiatric conditions^2,3,7^, GABA-mediated signaling by neuronal progenitors/neurons during development has been extensively studied^8,9,10^. Brain development, however, is not limited to neuronal changes but is also supported by concomitant development of its vasculature. After establishment of the periventricular vascular gradient by embryonic day 11^11^, excitatory glutamatergic projection neurons and inhibitory GABAergic interneurons navigate along diverse courses from ventricular zones, radially and tangentially, to adopt final laminar positions and synchronize cortical microcircuits^12,13,14^. While radial glia were established as the substrate for radial neuronal migration in the early seventies^15^, our recent studies have shown that the developing vascular system exquisitely patterned amidst neurons is the substrate for GABAergic neuronal tangential migration^16^. These findings highlighted the autonomy of periventricular versus pial vascular networks and revealed that they are independently capable of guiding deep versus superficial GABAergic neuronal populations *en route* to the cortex. Not only is the periventricular vascular network acting as a physical substrate for the migration of large populations of deep GABAergic neurons in the embryonic telencephalon, but also it holds the key to several novel developmental mechanisms. Many genes traditionally believed to be confined to GABAergic neurons and their precursors were found to be enriched in forebrain periventricular endothelial cells when compared to pial endothelial cells or control endothelial cells prepared from midbrain and hindbrain^16^. These results suggested that telencephalic endothelial cells house a novel GABA signaling pathway that is distinct from the traditional neuronal GABA signaling pathway with new significance for brain development and neuropsychiatric disease.

Several mouse models with abnormal GABA_A_ receptors and GABA function, which recapitulated defective behaviors similar to those seen in conditions like autism, epilepsy, schizophrenia, mood and anxiety disorders as well as human studies have been vital for understanding the pathobiology of these neurological and psychiatric illnesses^1,2,3,4,5,6,7,8,9,10,17,18,19,20^. However, all of the mouse models reported until now are systemic or region-specific knockouts of the GABA_A_ receptor-GABA pathway^2,8,9,17,18,19,20^. With such models, it is impossible to establish a cause-effect relationship between neuronal and endothelial development.

To discover the significance of GABA-related gene expression specifically in endothelial cells during embryonic development, we designed strategies to selectively modulate components of the endothelial GABA signaling pathway *in vivo*. This approach markedly affected endothelial GABA release levels, disturbed periventricular angiogenesis and in turn impaired GABAergic neuronal tangential migration in the embryonic brain. Concurrent vascular dysfunction and GABA cell deficits persisted in the postnatal cerebral cortex and manifested as diverse neuropsychiatric behavioral symptoms. Our results highlight the importance of a novel GABA signaling pathway operating via forebrain endothelial cells that has an intricate and powerful control of cerebral cortex development leaving its lasting signature on behavioral outcomes. It shows for the first time how prenatal forebrain angiogenesis has the remarkable potential to modulate postnatal and adult behaviors.

## Results

### Autonomous roles of endothelial GABA_A_ receptors in telencephalic development

**Figure 1:**
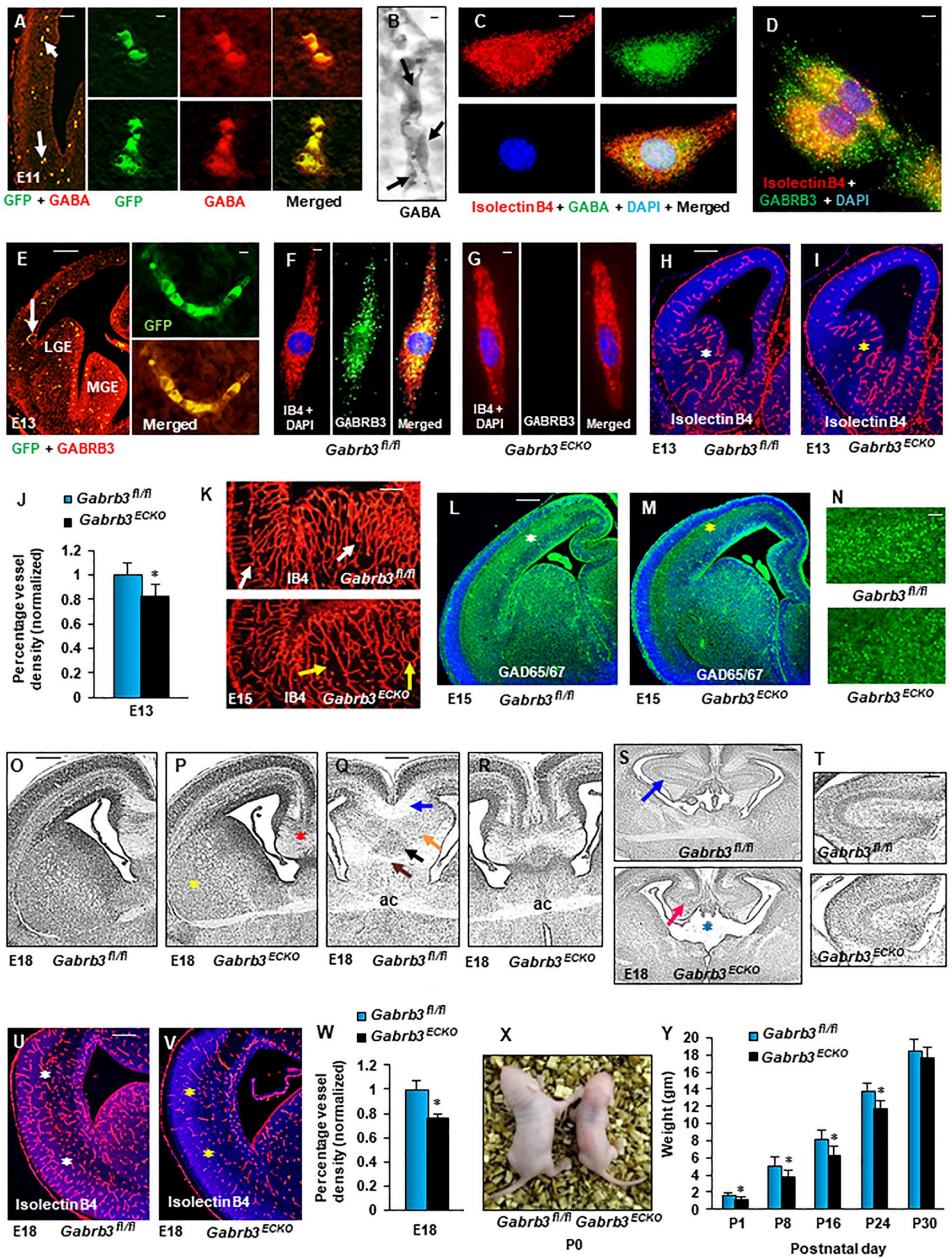
Endothelial *Gabrb3* regulates telencephalic development. **(A)** GABA expression (red) in E11 Tie2-GFP dorsal telencephalon with specific labeling in Tie2-GFP^+^ endothelial cells (co-label in yellow). White arrows illustrate high magnifications (20×) of endothelial cells showing individual and merged images of GFP and GABA. **(B)** A high-magnification image of GABA labeling of endothelial cells in a periventricular vessel from E12 neocortex obtained by DAB immunohistochemistry (60×). **(C)** Individual isolectin 4, GABA, DAPI and merged image of a periventricular endothelial cell (pv ec, 60×). **(D)** Co-labeled image of isolectin 4, GABRB3 and DAPI labeling of pv ecs (40×). **(E)** *In vivo* expression of GABRB3 in periventricular endothelial cells of Tie2-GFP telencephalon at E13. White arrow illustrates the region of high-magnification images (20×), which show GFP-positive endothelial cells lining a vessel, co-labeled with GABRB3. **(F)** Individual Isolectin 4, GABRB3, DAPI and merged image of a *Gabrb3^fl/fl^* pv ec (60×). **(G)** No GABRB3 expression in pv ecs was detected in *Gabrb3^ECKO^* embryos (60×). **(H**-**J)** Fewer isolectin B4^+^ vessels in E13 *Gabrb3^ECKO^* telencephalon (yellow asterisk, **I**) compared to *Gabrb3^fl/fl^* telencephalon (white asterisk, **H**). **(J)** Morphometric analysis of isolectin B4 labeling revealed significant reduction in vessel densities in E13 *Gabrb3^ECKO^* telencephalon; Data represent mean ± SD (*n* = 8, ^*^P < 0.05, Student's *t*-test). **(K)** While the tube-like plexus of periventricular vessels, labeled by isolectin B4, in the ganglionic eminence and dorsal telencephalon was continuous and well formed in *Gabrb3^fl/fl^* telencephalon, (white arrows), it was discontinuous and irregular (yellow arrows) in *Gabrb3^ECKO^* telencephalon. **(L**, **M)** GAD65/67 immunoreactivity showed decreased stream of GABA neurons in E15 *Gabrb3^ECKO^* telencephalon (yellow asterisk, **M**) when compared to *Gabrb3^fl/fl^* telencephalon (white asterisk, **L**). **(N)** High-magnification image (40×) revealing fewer GAD65/67 cells in *Gabrb3^ECKO^* dorsal telencephalon versus *Gabrb3^fl/fl^* telencephalon. **(O**-**T)** H&E stainings revealed no marked changes in cortical lamination in E18 *Gabrb3^ECKO^* dorso-lateral telencephalon **(P)** in comparison with *Gabrb3^fl/fl^* telencephalon **(O)**. However, morphological abnormalities were observed in medial *Gabrb3^ECKO^* telencephalon (red asterisk, **P**). Striatal compartments were enlarged in *Gabrb3^ECKO^* telencephalon (yellow asterisk, **P**). The corpus callosum (blue arrow), hippocampus oriens layer (orange arrow), triangular septal nucleus (black arrow) and ventral hippocampal commissure (brown arrow) were normally formed in *Gabrb3^fl/fl^* telencephalon **(Q)** but perturbed in *Gabrb3^ECKO^* telencephalon **(R)**. The two limbs of the anterior commissure (ac) crossed at the midline in both *Gabrb3^fl/fl^* and *Gabrb3^ECKO^* embryos **(Q**, **R)**. Ventricular defects (blue asterisk, **S**) and reduced hippocampus (red arrow, **S**) were observed in E18 *Gabrb3^ECKO^* telencephalon in comparison to *Gabrb3^fl/fl^* telencephalon (blue arrow, **S**). **(T)** High-magnification images of hippocampus from S. **(U**, **V)** Fewer isolectin B4^+^ vessels in E18 *Gabrb3^ECKO^* pallium (yellow asterisks, **V**) compared with *Gabrb3^fl/fl^* pallium (white asterisks, **U**). **(W)** Significant reduction in cortical vessel densities in E18 *Gabrb3^ECKO^* embryos; Data represent mean ± SD (*n* = 8, ^*^P < 0.05, Student's *t*-test). **(X)** *Gabrb3^ECKO^* mice at P0 were smaller in size than *Gabrb3^fl/fl^* mice. **(Y)** Weight chart of *Gabrb3^ECKO^* mice compared to *Gabrb3^fl/fl^* mice from P1 to P30; Data represent mean ± SD (*n* = 12, ^*^P < 0.05, Student's *t*-test). Scale bars: **A**, 60 μm (applies to **N**); **B**, 30 μm (applies to **D**); **C**, 15 μm; (applies to **F**, **G**), **E**, 100 μm; (applies to **H**, **I**, **K**-**M**, **O**-**S**, **U**, **V**); **T**, 40 μm, high-magnification insets in **A** and **E**, 30 μm.

The earliest GABA expression in the embryonic cerebral cortex has been described on embryonic day 10 (E10) near the pial surface and it has been difficult to explain the diffuse GABA staining present throughout the neuroepithelium between E10 and E12, even before GABAergic interneurons arrive^21^. With the use of Tie2-GFP mice, we observed that GFP^+^ endothelial cells of the periventricular vessel gradient^11^ at E11 express GABA (Figure 1A). GABA expression was distinct and robust in periventricular vessels of the E12 dorsal telencephalon (Figure 1B) and cultured periventricular endothelial cells (Figure 1C)^16^. Additionally, we observed expression of several GABA_A_ receptor subunits in periventricular endothelial cells with GABA_A_ receptor β3 subunit (Gabrb3) showing enriched expression, *in vitro* and *in vivo* (Supplementary information, Figure S1A, Figure 1D, 1E)^16^. To discover the functional significance of endothelial GABA_A_ receptors *in vivo*, we selectively deleted GABA_A_ receptor β3 subunit from endothelial cells to generate *Gabrb3* endothelial cell conditional knockout *Gabrb3^ΔTie2-Cre^* (also named *Gabrb3^ECKO^*) mice. While *Gabrb3^fl/fl^* endothelial cells robustly expressed GABRB3 (Figure 1F, Supplementary information, Figure S1B), endothelial cells of *Gabrb3^ECKO^* telencephalon did not express GABRB3 confirming its deletion (Figure 1G, Supplementary information, Figure S1C). Labeling with multiple markers of vessel components, isolectin B4 and CD31/PECAM-1 revealed reductions in vessel density and pattern formation in E13 *Gabrb3^ECKO^* telencephalon (Figure 1H-1J, Supplementary information, Figure S1D-S1I). The tangential stream of GABAergic neurons that migrate from basal to dorsal telencephalon, examined with GAD65/67 immunoreactivity, was reduced in *Gabrb3^ECKO^* telencephalon when compared to *Gabrb3^fl/fl^* telencephalon at E13 (Supplementary information, Figure S1J, S1K). Vascular reductions continued in E15 *Gabrb3^ECKO^* telencephalon (Supplementary information, Figure S2A-S2E). The rhombic vascular patterns in the ganglionic eminence (GE) that ensheath deep GABAergic neuronal populations in a tube-like form^16^ were well formed in E15 *Gabrb3^fl/fl^* telencephalon, but continued to be disrupted in *Gabrb3^ECKO^* telencephalon (Figure 1K), along with concurrent reduction in GAD65/67 immunoreactivity (Figure 1L-1N). In histological stainings, cortical lamination in E18 dorso-lateral *Gabrb3^ECKO^* telencephalon appeared normal (Figure 1O, 1P) but the medial telencephalon showed morphological defects (Figure 1P, 1R). While in *Gabrb3^fl/fl^* telencephalon, corpus callosum, hippocampal layer stratum oriens, triangular septal nucleus and ventral hippocampal commissure could be clearly visualized (Figure 1Q), these anatomical landmarks were perturbed in *Gabrb3^ECKO^* telencephalon (Figure 1R). Ventricular abnormalities (Figure 1S), reduced hippocampus (Figure 1S, 1T) and enlarged striatal compartments (Figure 1P) were frequently observed in *Gabrb3^ECKO^* telencephalon. Cortical vascular densities were reduced in E18 *Gabrb3^ECKO^* telencephalon when compared to *Gabrb3^fl/fl^* telencephalon (Figure 1U-1W, Supplementary information, Figure S2F, S2G). Significant changes in blood-brain barrier (BBB) properties were not observed in *Gabrb3^ECKO^* versus *Gabrb3^fl/fl^* telencephalon. Subtle changes in tight junction protein, claudin 5 expression (Supplementary information, Figure S2H, S2I) as well as dilated and abnormally stretched vessels were noticed by immunoglobulin G (IgG) staining in *Gabrb3^ECKO^* telencephalon, when compared to *Gabrb3^fl/fl^* telencephalon, but no IgG leakage was observed (Supplementary information, Figure S2J, S2K). *Gabrb3^ECKO^* mice were smaller in size than *Gabrb3^fl/fl^* mice at birth and during postnatal stages (Figure 1X, 1Y), but showed long-term survival into adulthood. This provided us with a unique opportunity for studying the consequences of developmental perturbations due to loss of endothelial *Gabrb3* in the mature brain.

### Postnatal consequences of selective loss of endothelial Gabrb3

**Figure 2:**
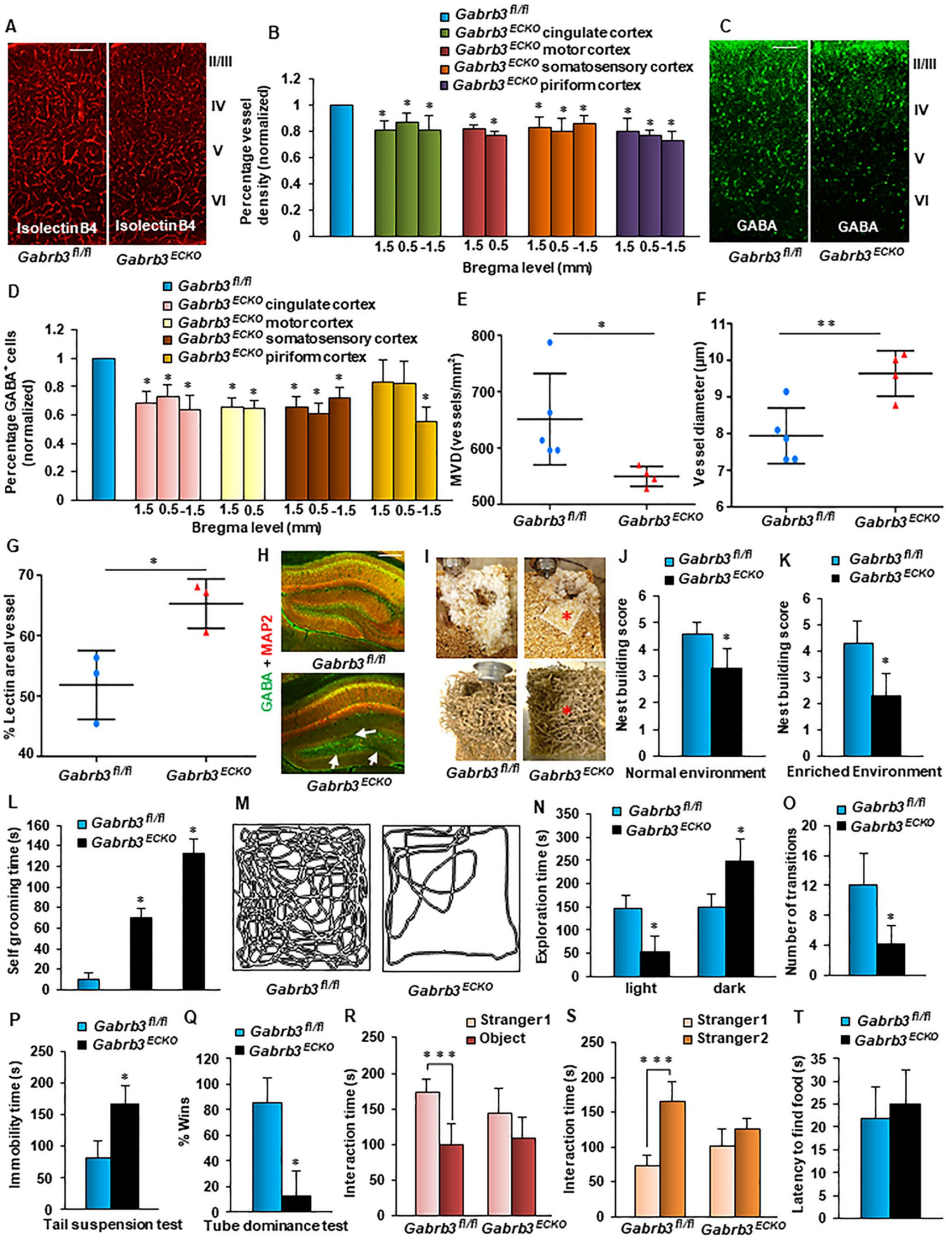
Vascular and GABA cell deficits in *Gabrb3^ECKO^* adult brain and concurrent behavioral deficits. **(A**, **B)** Isolectin B4-labeled vessels were significantly reduced in cingulate, motor, somatosensory and piriform cortex of *Gabrb3^ECKO^* mice at P90 when compared to *Gabrb3^fl/fl^* mice (at 1.5, 0.5 and −1.5 bregma levels). Somatosensory cortex was depicted in **A**. Vessel quantification was depicted in **B**; Data represent mean ± SD (*n* = 8, ^*^P < 0.05; Student's *t*-test). **(C**, **D)** A reduction in GABA^+^ cells was observed in *Gabrb3^ECKO^* cingulate, motor and somatosensory cortex at all bregma levels analyzed. In the *Gabrb3^ECKO^* piriform cortex, significant reduction of GABA^+^ cells was observed only in the −1.5 bregma level; Data represent mean ± SD (*n* = 8, ^*^P < 0.05, Student's *t*-test). **(E)** Similar reduction in density of CD31^+^ microvessels was observed in the *Gabrb3^ECKO^* cingulate cortex; Data represent mean ± SD (^*^P < 0.05; Student's *t*-test). **(F)** Vessel diameters were significantly increased in *Gabrb3^ECKO^* cingulate cortex; Data represent mean ± SD (^*^P < 0.05, Student's *t*-test). **(G)** The average lectin^+^ area per perfused vessel was also increased in *Gabrb3^ECKO^* cortex when compared to *Gabrb3^fl/fl^* cortex; Data represent mean ± SD (^*^P < 0.05, Student's *t*-test). **(H)** GABA immunohistochemistry showed a reduction in GABAergic neurons in *Gabrb3^ECKO^* hippocampus (white arrows) when compared to *Gabrb3^fl/fl^* hippocampus. **(I-K)** To test for home cage social behavior, *Gabrb3^ECKO^* and *Gabrb3^fl/fl^* mice were housed individually in cages containing wood chip bedding and two nestlets (upper panels, **I**) or shredded paper (lower panels, **I**). After 1 h (with nestlet) and 24 h (with shredded paper), untorn nestlet and constitution of built nest were assessed, according to a five-point scale. *Gabrb3^ECKO^* mice failed to build proper nests like *Gabrb3^fl/fl^* mice as quantified by untorn nestlet or scattered paper (red asterisks, **I**) and nest building score **(J**, **K)**; Data represent mean ± SD (*n* = 15, ^*^P < 0.05, Student's *t*-test). **(L)** *Gabrb3^ECKO^* mice showed moderate to extensive grooming when compared to *Gabrb3^fl/fl^* mice; Data represent mean ± SD (*n* = 14, ^*^P < 0.05, Student's *t*-test). **(M)** In a light-dark box test, the movement trace showed that *Gabrb3^ECKO^* mice moved far less in the light side when compared to *Gabrb3^fl/fl^* mice. **(N)** Quantification of exploration time showed that *Gabrb3^ECKO^* mice spent less time in the light side and more time in the dark side of the box when compared to *Gabrb3^fl/fl^* mice; Data represent mean ± SD (*n* = 15, ^*^P < 0.05, Student's *t*-test). **(O)** *Gabrb3^ECKO^* mice made fewer transitions into the light side when compared to *Gabrb3^fl/fl^* mice; Data represent mean ± SD (*n* = 15, ^*^P < 0.05, Student's *t*-test). **(P)** *Gabrb3^ECKO^* mice showed longer periods of immobility in a tail suspension test; Data represent mean ± SD (*n* = 12, ^*^P < 0.05, Student's *t*-test). **(Q)** *Gabrb3^ECKO^* mice had fewer wins in a tube dominance test when compared to *Gabrb3^fl/fl^* mice; Data represent mean ± SD (*n* = 16, ^*^P < 0.05, Student's *t*-test). **(R)** In a social interaction test *Gabrb3^ECKO^* mice showed no significant difference in time spent between stranger mouse and object unlike floxed littermates; Data represent mean ± SD (*n* = 12, ^*^P < 0.05, Student's *t*-test). **(S)** In the social novelty phase, while *Gabrb3^fl/fl^* mice showed a significant preference for novel stranger 2 over the now familiar stranger 1, *Gabrb3^ECKO^* mice showed no obvious preference; Data represent mean ± SD (*n* = 12, ^*^P < 0.05; Student's *t*-test). **(T)** No olfaction defects in *Gabrb3^ECKO^* mice as seen in a buried food test; Data represent mean ± SD (*n* = 14). Scale bars: **A**, 100 μm; (applies to **C**, **H**).

The vascular and GABA cell deficit observed in the *Gabrb3^ECKO^* embryonic brain (Figure 1) was also reflected in *Gabrb3^ECKO^* adult brain (P90) (Figure 2A-2D). Significantly affected regions in *Gabrb3^ECKO^* adult brain were the cingulate cortex, motor cortex and somatosensory cortex, in which concurrent reductions in isolectin B4^+^ vessels and GABA^+^ interneurons were observed (Figure 2A-2D). In the piriform cortex of *Gabrb3^ECKO^* mice, vascular reduction was observed at the three bregma levels analyzed (Figure 2B), but GABAergic neurons were reduced significantly only at −1.5 bregma level (Figure 2D). We next used a combination of retro-orbital lectin perfusion and CD31 immunohistochemistry (IHC) techniques, and focused on the P90 cingulate cortex for further analysis of vasculature. Microvessel densities were significantly reduced in *Gabrb3^ECKO^* cortex in comparison with *Gabrb3^fl/fl^* cortex (Figure 2E). Additionally, vessel diameters were markedly increased in *Gabrb3^ECKO^* cortex indicative of morphological alterations when compared to controls (Figure 2F). The average lectin^+^ area per vessel was also increased in *Gabrb3^ECKO^* cortex (Figure 2G). Larger vessel diameters likely correlated with the increased perfusion and are indicative of functional changes in *Gabrb3^ECKO^* vessels. Enlarged vessels continued to be detected by IgG staining in *Gabrb3^ECKO^* cortex (Supplementary information, Figure S2L, S2M). The hippocampus of *Gabrb3^ECKO^* mice at P90 also showed a deficit in GABAergic neurons when compared to *Gabrb3^fl/fl^* mice (Figure 2H). The decreased number of interneurons in *Gabrb3^ECKO^* cortex was not due to apoptosis as confirmed by anti-active caspase-3 IHC (Supplementary information, Figure S2N, S2O).

We next questioned whether the vascular abnormalities and reduction of GABAergic neurons in *Gabrb3^ECKO^* cerebral cortex contributed to altered behavior. As a first indication of pathological behavior, 15% of the *Gabrb3^ECKO^* mice showed seizure-like symptoms from P14 onward. Common characteristics that overlap across many neuropsychiatric disease categories are impaired reciprocal social interactions, communication deficits and heightened anxiety. Therefore we performed behavioral tests to screen for stress, anxiety and sociability in *Gabrb3^ECKO^* mice. Mice are expert and flexible nest builders, so *Gabrb3^ECKO^* and *Gabrb3^fl/fl^* mice were housed individually in cages containing wood chip bedding and two nestlets (pressed cotton) or more naturalistic material like shredded paper strips. *Gabrb3^ECKO^* mice showed poor nest building behavior in both normal and enriched environments (Figure 2I-2K) as well as moderate to severe grooming (Figure 2L) indicative of impaired home cage social behavior and increased stress/anxiety^22^. Anxiety was also assessed with the classic light-dark avoidance test, which triggers a struggle between the desires to explore an unknown area versus dislike of a brightly lit open space^22^. *Gabrb3^fl/fl^* mice made several entries into the brightened space and spent equivalent times between the light and dark sides of the open field (Figure 2M-2O). On the other hand, *Gabrb3^ECKO^* mice showed an aversion to brightly lit open space and preferred the dark area (Figure 2M-2O).

We next performed the tail suspension test, a screening test for depression in which normal mice will struggle to face upward and climb to a solid surface. When the animal stops struggling and hangs immobile, it is considered to have 'given up'. *Gabrb3^ECKO^* mice showed longer periods of immobility than wild-type (WT) littermates in the tail suspension test that is characteristic of a depressive-like state (Figure 2P). We used the tube dominance test to assess cognition in *Gabrb3^ECKO^* mice, in particular social dominance through measurement of aggression. Both *Gabrb3^fl/fl^* and *Gabrb3^ECKO^* mice were released into opposite ends of a tube and evaluated for the winner who forced its opponent out of the tube. *Gabrb3^ECKO^* mice showed fewer wins in a tube dominance test in comparison to their WT littermates (Figure 2Q). To test for social communication, a three-chambered social approach task (Supplementary information, Figure S3) was used in which we scored time spent in a side chamber with a novel mouse versus time spent in a side chamber with a non-social novel object as a measure of sociability. While *Gabrb3^fl/fl^* mice showed preference for a stranger mouse over an inanimate object, *Gabrb3^ECKO^* mice showed no preference for the stranger mouse and spent approximately similar time investigating stranger and object signifying impaired sociability (Figure 2R). In the social novelty phase, when a new stranger mouse was introduced into the previously empty cylinder, *Gabrb3^fl/fl^* mice showed a marked preference for stranger 2 versus the now familiar stranger 1, while *Gabrb3^ECKO^* mice did not show a significant preference for stranger 2 versus stranger 1 indicative of decreased social motivation, memory and novelty exploration (Figure 2S). We confirmed that the abnormal social behavior was not due to an olfaction defect since *Gabrb3^ECKO^* mice performed slightly better than *Gabrb3^fl/fl^* mice in the buried food olfaction test (Figure 2T). *Gabrb3^ECKO^* mice did not show gender-specific differences in behavior in all of these behavioral assays (Figure 2I-2S). These results provided novel evidence that prenatal loss of a single GABA_A_ receptor subunit from endothelial cells is sufficient to modulate postnatal behavior. It therefore became critical to gain mechanistic insights into endothelial GABA_A_ receptor actions during embryonic brain development.

### Mechanistic insights into endothelial GABA_A_ receptor function and dysfunction

**Figure 3:**
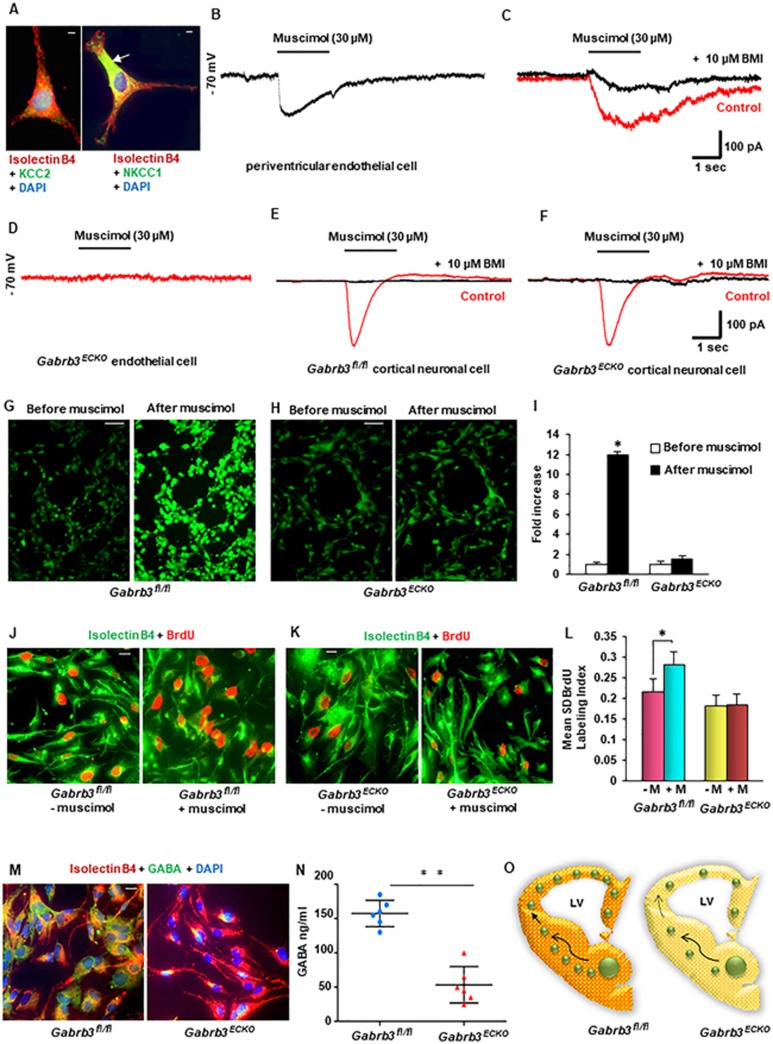
Mechanisms underlying endothelial *Gabrb3*'s actions. **(A)** Co-labeled image of isolectin B4, KCC2/NKCC1 and DAPI in periventricular endothelial cells. **(B)** The endothelial GABA_A_ receptor on periventricular endothelial cells is functional. Focal application of muscimol (30 μM) evoked an inward current consistently in whole-cell voltage-clamp recording of periventricular endothelial cells held at −70 mV (92.5 ± 16.3 pA, *n* = 8). **(C)** Traces showed an inward current of 100 pA induced by muscimol (30 μM) that was blocked by BMI (10 μM). **(D)** Muscimol application (30 μM) produced no current response in *Gabrb3^ECKO^* periventricular endothelial cells. **(E**, **F)** Traces showed inward currents of 100 pA induced by muscimol (30 μM) and blocked by BMI (10 μM) in *Gabrb3^fl/fl^* **(E)** and *Gabrb3^ECKO^* **(F)** cortical neuronal cells. **(G**, **H)** Increase of intracellular calcium upon muscimol treatment (30 μM) was significantly retarded in *Gabrb3^ECKO^* periventricular endothelial cells **(H)** when compared to the control. **(I)** Calcium imaging data were quantified by normalizing the values after muscimol application to that before muscimol application; Data represent mean ± SD (*n* = 7, ^*^P < 0.05, Student's *t*-test). **(J**-**L)** With or without muscimol application, *Gabrb3^fl/fl^* and *Gabrb3^ECKO^* periventricular endothelial cells were exposed to BrdU (1 mM BrdU per ml medium) for 1 h followed by Isolectin B4/BrdU double labeling. Muscimol application significantly increased cell proliferation in *Gabrb3^fl/fl^* periventricular endothelial cells, but *Gabrb3^ECKO^* periventricular endothelial cells showed no change. BrdU-labeling indices were quantified in **L**; Data represent mean ± SD (*n* = 7, ^*^P < 0.05; Student's *t*-test; 'M': muscimol). **(M)** Co-labeling with isolectin B4 and GABA antibodies showed that GABA expression was significantly downregulated in *Gabrb3^ECKO^* periventricular endothelial cells when compared to *Gabrb3^fl/fl^* endothelial cells. **(N)** As a result, GABA secretion from E15 *Gabrb3^ECKO^* periventricular endothelial cells measured by ELISA was significantly decreased when compared to *Gabrb3^fl/fl^* endothelial cells; Data represent mean ± SD (*n* = 6, ^*^P < 0.05, Student's *t*-test). **(O)** A diagrammatic illustration of how endothelial cell-secreted GABA influences critical events during brain development. Wild-type embryonic telencephalon with normal periventricular vascular network (red lattice pattern) and normal endothelial GABA signaling pathway (orange yellow hue) promotes tangential GABAergic neuronal migration (green circles) from the ventral telencephalon where they originate (big green circle). In *Gabrb3^ECKO^* telencephalon, there is a partial loss of endothelial GABA secretion (light yellowish hue). This affects periventricular angiogenesis (dotted red pattern) and GABAergic neuronal tangential migration with reduction in GABAergic neurons in the developing neocortex. Scale bars: **A**, 15 μm; **G**, 100 μm; (applies to **H**), **J**, 50 μm; (applies to **K**, **M**).

We first questioned whether embryonic forebrain endothelial cells possess functional GABA_A_ receptors. WT periventricular endothelial cells showed higher expression of the cation-chloride cotransporter — NKCC1 versus KCC2 (Figure 3A), similar to embryonic neurons. We prepared pure cultures of periventricular endothelial cells from E15 CD1 embryonic telencephalon and tested if endothelial GABA_A_ receptors were able to respond to the GABA_A_ receptor agonist muscimol. We found that application of muscimol in whole-cell patch-clamp recording of periventricular endothelial cells at a holding potential of −70 mV, resulted in an inward current (Figure 3B). The muscimol induced inward current was blocked by application of the GABA_A_ receptor antagonist bicuculline methiodide (BMI) (Figure 3C). This pharmacological profile provided evidence that periventricular endothelial cells have functional GABA_A_ receptors that account for the GABA responses. Muscimol application induced no current response in *Gabrb3^ECKO^* periventricular endothelial cells (Figure 3D). Muscimol application induced inward currents in *Gabrb3^fl/fl^* and *Gabrb3^ECKO^* cortical neuronal cells that were blocked by BMI (Figure 3E, 3F). These results ascertained that there was no change in the GABA_A_ receptor response in cortical neuronal cells and emphasized the specific loss of GABA_A_ receptor function in endothelial cells. Furthermore, muscimol application produced an increase of intracellular calcium in *Gabrb3^fl/fl^* periventricular endothelial cells in calcium imaging assays, but no marked increase in intracellular calcium in *Gabrb3^ECKO^* periventricular endothelial cells (Figure 3G-3I). Calcium transients have been shown to tightly regulate proliferation in many cell types, including cells in the neocortex^23,24^. Ca^2+^ influx can influence advancement of progenitor cells through the cells cycle. Transitions of cells from G1 to S phase and progression through M phase have been reported to be highly calcium dependent^25,26^. Since GABA_A_ receptor activation in *Gabrb3^fl/fl^* periventricular endothelial cells leads to an influx of Ca^2+^ that is likely to influence cell proliferation, we incubated *Gabrb3^fl/fl^* and *Gabrb3^ECKO^* periventricular endothelial cells isolated from E15 telencephalon in the presence of muscimol and the mitotic marker 5-bromo-2′-deoxyuridine (BrdU) to examine the impact on proliferation of these cells. Muscimol application significantly increased proliferation in *Gabrb3^fl/fl^* endothelial cells (Figure 3J, 3L). However, there was no discernible difference in *Gabrb3^ECKO^* endothelial cell proliferation before or after muscimol application (Figure 3K, 3L). Together, these results indicate that GABA_A_ receptors in *Gabrb3^ECKO^* periventricular endothelial cells are dysfunctional and elucidate how activation of endothelial GABA_A_ receptors modulates endothelial cell proliferation and angiogenesis.

Another interesting finding is that endothelial-specific deletion of *Gabrb3* significantly decreased GABA expression in embryonic periventricular endothelial cells (Figure 3M, Supplementary information, Figure S4A, S4B). We observed robust GABA expression by IHC in control periventricular endothelial cells and this is illustrated at both low and high magnifications (Figure 3M, Supplementary information, Figure S4A). All *Gabrb3^ECKO^* endothelial cells, in sharp contrast showed a marked reduction in GABA expression (Figure 3M, Supplementary information, Figure S4B). Variability in GABA expression with respect to fold decrease was observed in the *Gabrb3^ECKO^* endothelial cell population and this was quantified (Supplementary information, Figure S4C). Next, we measured secreted GABA by ELISA from *Gabrb3^fl/fl^* and *Gabrb3^ECKO^* periventricular endothelial cells. As expected, there was a significant reduction in GABA secretion upon *Gabrb3* knockout (Figure 3N). These data lay the foundation for a novel positive feedback signaling pathway in endothelial cells that functions via GABA_A_ receptor-mediated GABA release. Since pial endothelial cells do not express GABRB3^16^, they are unaffected by the deletion, and GABA secretion from these cells is unaffected (Supplementary information, Figure S4D). Thus the *Gabrb3^ECKO^* mouse is a model of partial loss of endothelial cell-secreted GABA in the embryonic telencephalon. Together these observations suggested that loss of functional endothelial GABA_A_ receptors and partial loss of endothelial GABA can impair telencephalic angiogenesis and angiogenesis-guided GABAergic neuronal migration *in vivo* (Figure 3O) as observed in Figure 1. The consequences persist in the adult brain, reflecting as reduced vascular densities and reduction of cortical interneurons and manifest as multifaceted behavioral deficits common in many overlapping psychiatric disease symptoms (Figure 2).

### The importance of endothelial cell-derived GABA for telencephalic development

**Figure 4:**
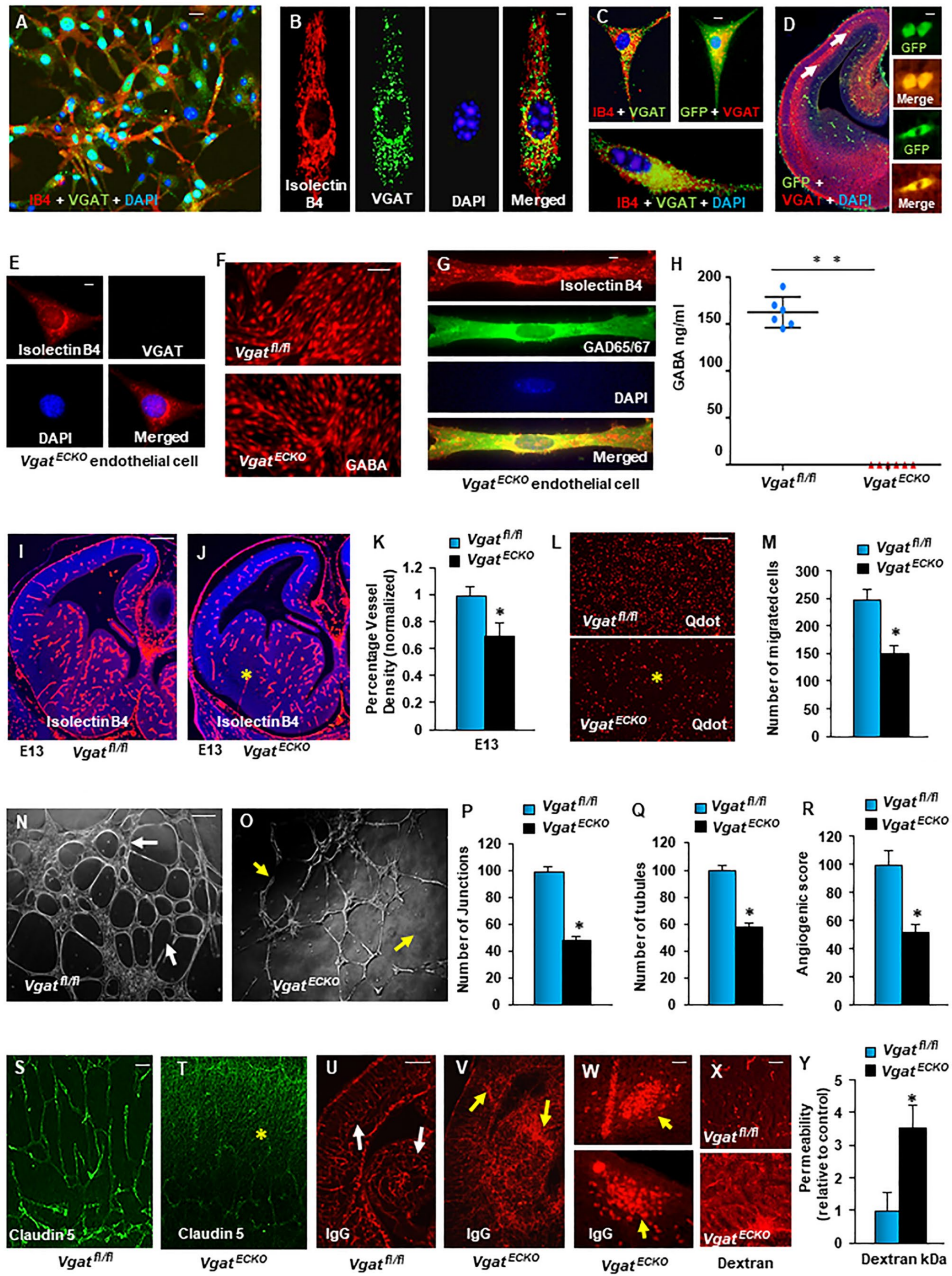
Abolishing endothelial GABA release and its effect on telencephalic angiogenesis **(A)** A low-magnification co-labeled image of Isolectin B4, VGAT and DAPI labeling of periventricular endothelial cells (pv ecs). **(B)** High-magnification image of Isolectin B4, VGAT and DAPI labeling of a pv ec (60×). **(C)** Different morphologies of Isolectin B4 (IB4) and Tie2-GFP-labeled ecs expressing VGAT (60×). **(D)** Low- and high-magnification images showing specifically *in vivo* expression of VGAT in endothelial cells of E13 Tie 2-GFP telencephalon. White arrows point to cells that were magnified. **(E)** No VGAT expression was detected in *Vgat^ECKO^* pv ecs (60×). **(F**, **G)** Low- and high-magnification images showing that expression of GABA **(F)** and GAD65/67 **(G)** was not affected in *Vgat^ECKO^* pv ecs. **(H)** Successful elimination of GABA secretion from embryonic *Vgat^ECKO^* pv ecs measured by ELISA; Data represent mean ± SD (*n* = 6, ^*^P < 0.05, Student's *t*-test). **(I**-**K)** Isolectin B4 labeling revealed a significant reduction in vessels in E13 *Vgat^ECKO^* telencephalon (yellow asterisk, **J**) when compared to *Vgat^fl/fl^* telencephalon **(I)**. **(K)** Quantification of vessel densities; Data represent mean ± SD (*n* = 6, ^*^P < 0.05, Student's *t*-test). **(L)** The migratory behavior of Qdot-labeled *Vgat^ECKO^* pv ecs was decreased (yellow asterisk) compared to *Vgat^fl/fl^* pv ecs. Representative images from the transwell migration assay are shown. **(M)** Quantification of the number of migrated cells per field from each group (*n* = 8, ^*^P < 0.05, mean ± SD. Student's *t*-test). **(N)** *Vgat^fl/fl^* pv ecs showed robust tube formation in an angiogenesis assay on matrigel (white arrows) reflecting their high angiogenic potential. **(O)** *Vgat^ECKO^* pv ecs failed to form robust tubes (yellow arrows), signifying impaired angiogenesis. **(P**-**R)** Quantification of number of junctions and tubules analyzed by Wimasis and quantification of the angiogenesis score^28^; Data represent mean ± SD (*n* = 10, ^*^P < 0.05, Student's *t*-test). **(S**, **T)** Claudin 5 expression was decreased in E16 *Vgat^ECKO^* dorsal telencephalon **(T)** when compared to *Vgat^fl/fl^* **(S)** telencephalon, illustrating loss of tight junctions (*n* = 10). **(U**, **V)** Images of IgG staining from E17 *Vgat^fl/fl^* and *Vgat^ECKO^* dorsal telencephalon. While IgG was localized to *Vgat^fl/fl^* vessels (white arrows, **U**), IgG leakage was observed from *Vgat^ECKO^* vessels in dorsal and medial telencephalon (yellow arrows, **V**). **(W)** High-magnification images of IgG leakage (yellow arrows) from *Vgat^ECKO^* vessels in the dorsal telencephalon. **(X**, **Y)** E18 *Vgat^ECKO^* and littermate controls were given a trans-cardiac perfusion of biotinylated dextran. *Vgat^ECKO^* tissue sections stained with streptavidin-Alexa 594 showed increased fluorescence **(X)** which was quantified and permeability relative to control was graphed (**Y**; *n* = 10, ^*^P < 0.05, mean ± SD, Student's *t*-test). Scale bars: **A**, 50 μm (applies to **S**, **T**, **W**, **X**), **B**, 15 μm (applies to **C**, **E**, **G**), **D**, 100 μm (high-magnification inset 30 μm); **F**, 75 μm, **I**, 100 μm (applies to **I**, **J**, **L**, **N**, **O**, **U**, **V**).

Since partial loss of endothelial GABA during embryonic brain development resulted in long-term repercussions, we were curious as to the effect of complete loss of endothelial GABA release *in vivo* on brain development and postnatal behavior. We had detected both GAD1 and GAD2 expression in CNS endothelial cells^16^. Therefore, conditional deletion of GAD1 alone from endothelial cells will not be sufficient to deplete endothelial GABA due to presence of GAD2 and *vice versa*. We thus examined the expression of GATs, GABA transporters that can serve to store and release GABA. No GATs were present, but we found that *slc32a1* (also named *Vgat*, vesicular GABA transporter) mRNA was highly enriched in periventricular endothelial cells^16^. We did not find glycine receptors, glycine, β-alanine or taurine expression in periventricular endothelial cells, suggesting that a GABA pathway is exclusively active in this cell type during early embryonic development. This suggested that GABA release from periventricular endothelial cells may be vesicular and deletion of endothelial *Vgat* is likely to have profound influence on adjusting the balance of GABA release and local GABA concentrations. Therefore, VGAT expression in both WT and Tie2-GFP telencephalic endothelial cells was first confirmed by IHC and found to be robustly expressed both *in vitro* (Figure 4A-4C) and *in vivo* (Figure 4D). Next, we used *Tie2-cre* mice and *Vgat floxed* mice to selectively delete *Vgat* from endothelial cells and generate *Vgat* endothelial cell conditional knockout *Vgat^ΔTie2-cre^* (also named *Vgat^ECKO^*) mice. VGAT expression was not detected specifically in *Vgat^ECKO^* endothelial cells (Figure 4E, Supplementary information, Figure S5A, S5B). No marked change or variability in GABA or GAD65/67 expression was observed in *Vgat^ECKO^* periventricular endothelial cells when compared to controls (Figure 4F, 4G). Then, we determined whether endothelial GABA secretion was affected in *Vgat^ECKO^* embryonic telencephalon by isolating *Vgat^ECKO^* periventricular endothelial cells and testing for GABA secretion by ELISA. We found that deleting *Vgat* from endothelial cells successfully abolished GABA secretion during embryonic stages from periventricular endothelial cells (Figure 4H). These experiments confirmed that VGAT is the primary GABA transporter in periventricular endothelial cells and that GABA release by these endothelial cells is executed through a vesicular mechanism. Telencephalic angiogenesis was more markedly affected in *Vgat^ECKO^* telencephalon when compared to *Gabrb3^ECKO^* telencephalon. Vascular densities were significantly reduced in E13 *Vgat^ECKO^* telencephalon (Fig 4I-4K). Reduction in vessel density and loss of normal periventricular vessel plexus formation continued in E15 *Vgat^ECKO^* telencephalon (Supplementary information, Figure S5C-S5G). Cortical vessel densities in E18 *Vgat^ECKO^* telencephalon were also significantly decreased (Supplementary information, Figure S5H-S5J).

Next, we performed a transwell migration assay to examine migration of *Vgat^ECKO^* endothelial cells labeled with Qdot nanocrystals versus controls. *Vgat^ECKO^* endothelial cells showed a significant reduction in migration when compared to *Vgat^fl/fl^* endothelial cells (Figure 4L, 4M). Tube formation assays^27^ showed that the angiogenic potential of *Vgat^ECKO^* periventricular endothelial cells was significantly affected (Figure 4N-4R). The tube network was quantified to measure the two parameters: number of junctions and number of tubules, both of which showed a significant reduction in *Vgat^ECKO^* endothelial cells when compared to controls (Figure 4P, 4Q). *Vgat^ECKO^* endothelial cells formed fewer polygons or 'honey comb' structures, which indicated the lack of organizing into complex structures. Furthermore, we quantified the angiogenic score using published methodology^28^ by taking into account the number of sprouting cells, connected cells and polygons. The angiogenic score of *Vgat^ECKO^* endothelial cells was significantly reduced versus *Vgat^fl/fl^* endothelial cells (Figure 4R).

Since vascular endothelial cells that advance in the telencephalon form a functional BBB during embryogenesis^29^, and extrinsic addition of GABA (5 μM) was able to increase expression of claudin 5 in WT periventricular endothelial cells (Supplementary information, Figure S5K, S5L), we tested the barrier properties in *Vgat^fl/fl^* and *Vgat^ECKO^* dorsal telencephalon in detail. Claudin 5 showed robust expression in E16 *Vgat^fl/fl^* cortical blood vessels (Figure 4S) but *Vgat^ECKO^* vessels showed a loss of tight junctions and reduced claudin 5 expression (Figure 4T). Another tight junction protein, ZO-1, was also reduced in *Vgat^ECKO^* endothelial cells versus control (Supplementary information, Figure S5M, S5N). We next checked for leakage of IgG using IHC, since IgGs are restricted to the insides of vessels. Contrary to controls (Figure 4U), IgG leakage and extravascular IgG staining was observed in the dorsal and medial telencephalon (developing hippocampus) of E17 *Vgat^ECKO^* mice (Figure 4V, 4W). To better determine barrier properties, E18 *Vgat^ECKO^* mice and littermate controls were given a trans-cardiac perfusion of biotinylated dextran. *Vgat^ECKO^* mice showed an increase in tracer staining in the dorsal telencephalon indicative of increased vascular permeability (Figure 4X, 4Y). Together, these results suggested an impairment of the BBB in *Vgat^ECKO^* mice. It illustrates the importance of endothelial cell-derived GABA for angiogenesis and formation of barrier properties in the embryonic telencephalon.

### Endothelial GABA promotes long-distance GABAergic neuronal migration

**Figure 5:**
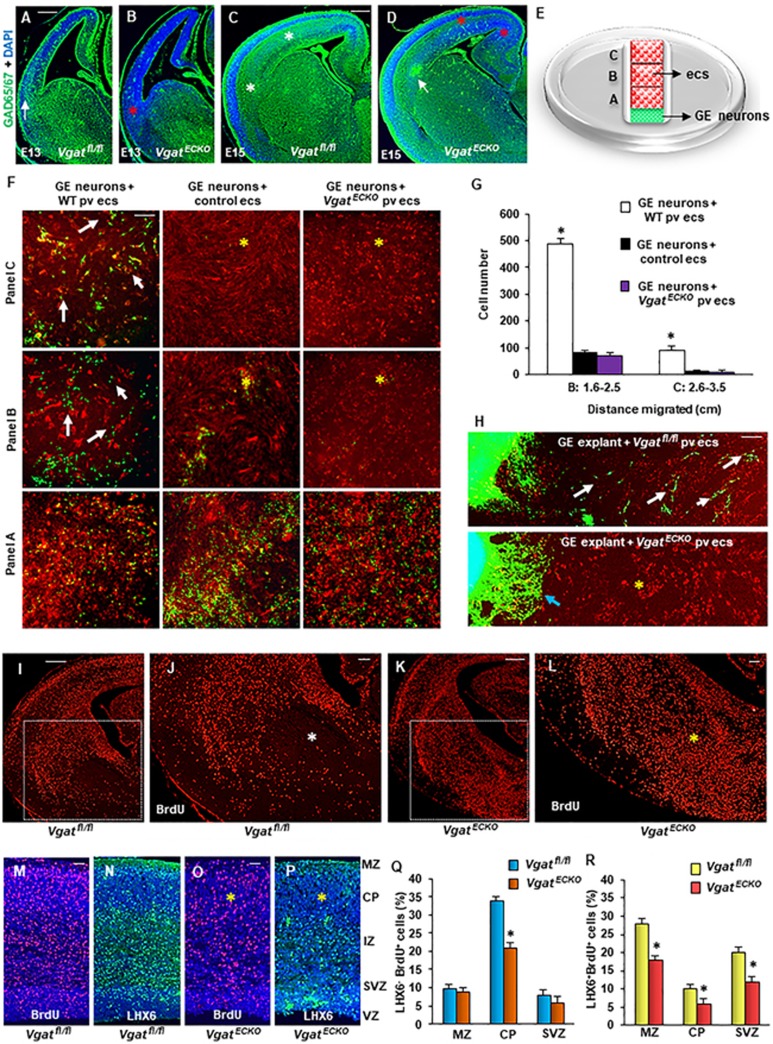
Endothelial cell-derived GABA is essential for long-distance GABA neuronal migration. **(A**-**D)** GAD65/67 immunoreactivity showed decreased stream of GABA neurons in E13 and E15 *Vgat^ECKO^* telencephalon (red asterisks **B**, **D**) when compared to *Vgat^fl/fl^* telencephalon (white arrow **A**; asterisks **C**). White arrow in **D** shows unusual GAD65/67+ve cell clusters in *Vgat^ECKO^* telencephalon. **(E)** Experiment schematic: WT periventricular endothelial cells (pv ecs), WT control ecs or *Vgat^ECKO^* pv ecs (that do not secrete GABA) were seeded in a specific track spanning a 35 mm culture dish (red dotted boxes). GE neurons from GAD65-GFP telencephalon were plated at one end of the track (green box). Neuronal migration was analyzed in three panels **A**-**C**. **(F)** Robust long-distance migration of GE neurons on WT pv ecs (white arrows) when compared to WT control ecs or *Vgat^ECKO^* pv ecs (yellow asterisks). **(G)** Quantification of cell migration in **(F)**; Data represent mean ± SD (*n* = 9, ^*^P < 0.05, Student's *t*-test). **(H)** Similar observations were noticed when GE explants were cultured on WT pv ecs or *Vgat^ECKO^* pv ecs. White arrows point to robust neuronal migration, blue arrow points to stalled cells and yellow asterisk reveals no migration. **(I**-**L)** Telencephalic coronal sections of E17 *Vgat^fl/fl^* **(I)** and *Vgat^ECKO^* **(K)** embryos that received a single BrdU injection at E13, showing immunohistochemistry results with anti-BrdU antibody. Insets in **(I)** and **(K)** are magnified in **(J)** and **(L)**. Several stalled BrdU^+^ cells were observed in *Vgat^ECKO^* ventral telencephalon (yellow asterisk, **L**) when compared to *Vgat^fl/fl^* ventral telencephalon (white asterisk, **J**). **(M**-**P)** Coronal sections through the dorsal telencephalon of E17 *Vgat^fl/fl^* **(M**, **N)** and *Vgat^ECKO^* **(O**, **P)** embryos that were injected with BrdU at E13, showing immunohistochemistry results for BrdU **(M**, **O)** and LHX6 **(N**, **P)**. **(Q**, **R)** Quantification of the distribution of E13 LHX6^−^ BrdU^+^ cells **(Q)** and LHX6^+^ BrdU^+^ cells **(R)** in *Vgat^fl/fl^* and *Vgat^ECKO^* E17 dorsal telencephalon; Data represent mean ± SD (*n* = 10, ^*^P < 0.05, Student's *t*-test). Scale bars: **A**, 100 μm (applies to **B**-**D**, **F**, **H**, **I**, **K**), **J**, 50 μm (applies to **L**, **M**-**P**).

By turning off endothelial GABA secretion during embryonic brain development, we were able to evaluate the significance of endothelial GABA for key events during brain development — neurogenesis and neuronal migration. No marked changes were seen in neuroepithelial cell proliferation in *Vgat^ECKO^* telencephalon as observed by the 2.0 h BrdU labeling index at early (E13) and late (E17) embryonic stages (Supplementary information, Figure S6). Since periventricular blood vessels have been shown to influence ventral telencephalic progenitors^30^, cell proliferation was further analyzed in the ventral telencephalon of E15 *Vgat^ECKO^* mice by examining interkinetic nuclear migration with phosphohistone 3 (PHH3), a specific marker for cells undergoing mitosis. There were no differences in the number of PHH3^+^ cells at the ventricular zone (VZ) of *Vgat^fl/fl^* versus *Vgat^ECKO^* ventral telencephalon. However, abnormal PHH3^+^ profiles were observed in the extra-VZ surface of *Vgat^ECKO^* ventral telencephalon, along with a small but significant increase in the number of PHH3^+^ cells (Supplementary information, Figure S7A-S7E). Ki67 labeling (a marker for all phases of the cell cycle: S, G2, M and G1) was increased at the extra-VZ surface of *Vgat^ECKO^* ventral telencephalon versus *Vgat^fl/fl^* ventral telencephalon (Supplementary information, Figure S7F, S7G). Some microtubule-associated protein 2 (MAP2) immunoreactive postmitotic neurons were observed within the neuroepithelium in the GE in the *Vgat^ECKO^* embryos, revealing evidence of impaired differentiation and perturbed neuronal migration (Supplementary information, Figure S7H, S7I). The GABAergic neurons in the *Vgat^ECKO^* telencephalon were significantly affected (Figure 5), while tyrosine hydroxylase^+^ neurons were not (Supplementary information, Figure S7J, S7K). The GABAergic neuronal tangential migratory profile, examined with GAD65/67 immunoreactivity was significantly reduced in E13 and E15 *Vgat^ECKO^* dorsal telencephalon when compared to *Vgat^fl/fl^* telencephalon (Figure 5A-5D). Since *Vgat^ECKO^* endothelial cells did not secrete GABA (Figure 4H), they were valuable for testing whether it was specifically endothelial cell-derived GABA that acted as a chemoattractant and provided directional cues to migrating neurons. Our experimental strategy was to seed either E15 WT periventricular endothelial cells, control endothelial cells (from WT midbrain and hindbrain combined) or *Vgat^ECKO^* periventricular endothelial cells (that do not secrete GABA) in a specific track spanning a 35 mm culture dish. WT GE-derived neurons from E15 GAD65-GFP telencephalon were plated at one end of the track (Figure 5E). GE neurons migrated robustly on a bed of WT periventricular endothelial cells from one end of the dish to the other (Figure 5F, 5G). The control (Figure 5F, 5G) confirmed that GE neuronal migration was specifically affected by periventricular endothelial cells. Most importantly, GE neurons failed to migrate long distance on *Vgat^ECKO^* periventricular endothelial cells that cannot secrete GABA (Figure 5F, 5G). Similar results were obtained when GE explants were cultured on *Vgat^fl/fl^* or *Vgat^ECKO^* periventricular endothelial cells (Figure 5H). Extrinsic addition of GABA (5 μM) was able to rescue GE neuronal migration on *Vgat^ECKO^* periventricular endothelial cells (Supplementary information, Figure S8) indicating the importance of the endothelial GABA source for neuronal migration. To test whether loss of endothelial GABA has consequences for GABA neuronal migration routes and distribution *in vivo*, we performed a BrdU birthdating study. We followed a single cohort of GE-derived interneurons by labeling cells born at E13 with a single BrdU pulse and analyzed their distribution in the E17 cortex. BrdU IHC by both fluorescence and DAB methods revealed several stalled BrdU^+^ cells in *Vgat^ECKO^* ventral telencephalon when compared to *Vgat^fl/fl^* telencephalon at both rostral and caudal levels indicative of abnormal GABAergic neuronal migration (Figure 5I-5L, Supplementary information, Figure S9). Double IHC for BrdU and LHX6 (a marker for migrating interneurons) highlighted layer-specific alterations in laminar targeting of GABAergic interneurons in *Vgat^ECKO^* developing neocortex (Figure 5M-5R). While LHX^−^ BrdU^+^ cells were significantly reduced only in the cortical plate (CP) suggesting that laminar positioning of cortical projection neurons may also be affected in the *Vgat^ECKO^* telencephalon (Figure 5Q), LHX6^+^ BrdU^+^ cells were significantly decreased in the marginal zone, CP and subventricular zone (SVZ) of *Vgat^ECKO^* telencephalon indicative of perturbed GABAergic neuronal tangential migration and final distribution (Figure 5R). Together, these results signify that endothelial cell-secreted GABA is essential for long-distance GABAergic neuronal migration in the embryonic telencephalon.

### Disturbances in radial neuronal migration in the absence of endothelial GABA

Earlier studies have shown that intermediate progenitors, the precursors of projection neurons in the developing neocortex are very closely associated with periventricular blood vessels and are influenced by vascular cues^31^. PHH3 labeling showed an increase in mitotic cells in the SVZ of E15 *Vgat^ECKO^* telencephalon, while no differences were observed in the ventricular border (Supplementary information, Figure S10A-S10D). The distribution of cells expressing the transcription factor Tbr2 that selectively marks intermediate progenitors, precursors of cortical projection neurons was next examined in the dorsal telencephalon. Tbr2-positive cells were predominant in the VZ/SVZ of *Vgat^fl/fl^* telencephalon, but ectopic increases in Tbr2-positive cells were observed in the intermediate zone (IZ) and CP of *Vgat^ECKO^* telencephalon (Supplementary information, Figure S10E-S10G). Spatial patterns of neuronal differentiation were examined by expression of MAP2 and cortical neuron population marker Tbr1, which marks CP and layer VI in WT embryos. MAP2-positive postmitotic neurons were detected within the SVZ in E15 *Vgat^ECKO^* telencephalon, revealing evidence of perturbed neuronal migration (Supplementary information, Figure S10H). Tbr1 immunoreactivity revealed a continuous uniform band of Tbr1-positive cells in the CP of E18 *Vgat^fl/fl^* embryos. On the other hand, abnormally positioned Tbr1-positive cells were detected trailing in the IZ of *Vgat^ECKO^* embryos (Supplementary information, Figure S10I, S10J). Together, these results indicate perturbations of radial neuronal migration in the absence of endothelial cell-secreted GABA during development.

### Gene expression profiling of *Vgat^ECKO^* telencephalon predicts its postnatal phenotype

**Figure 6:**
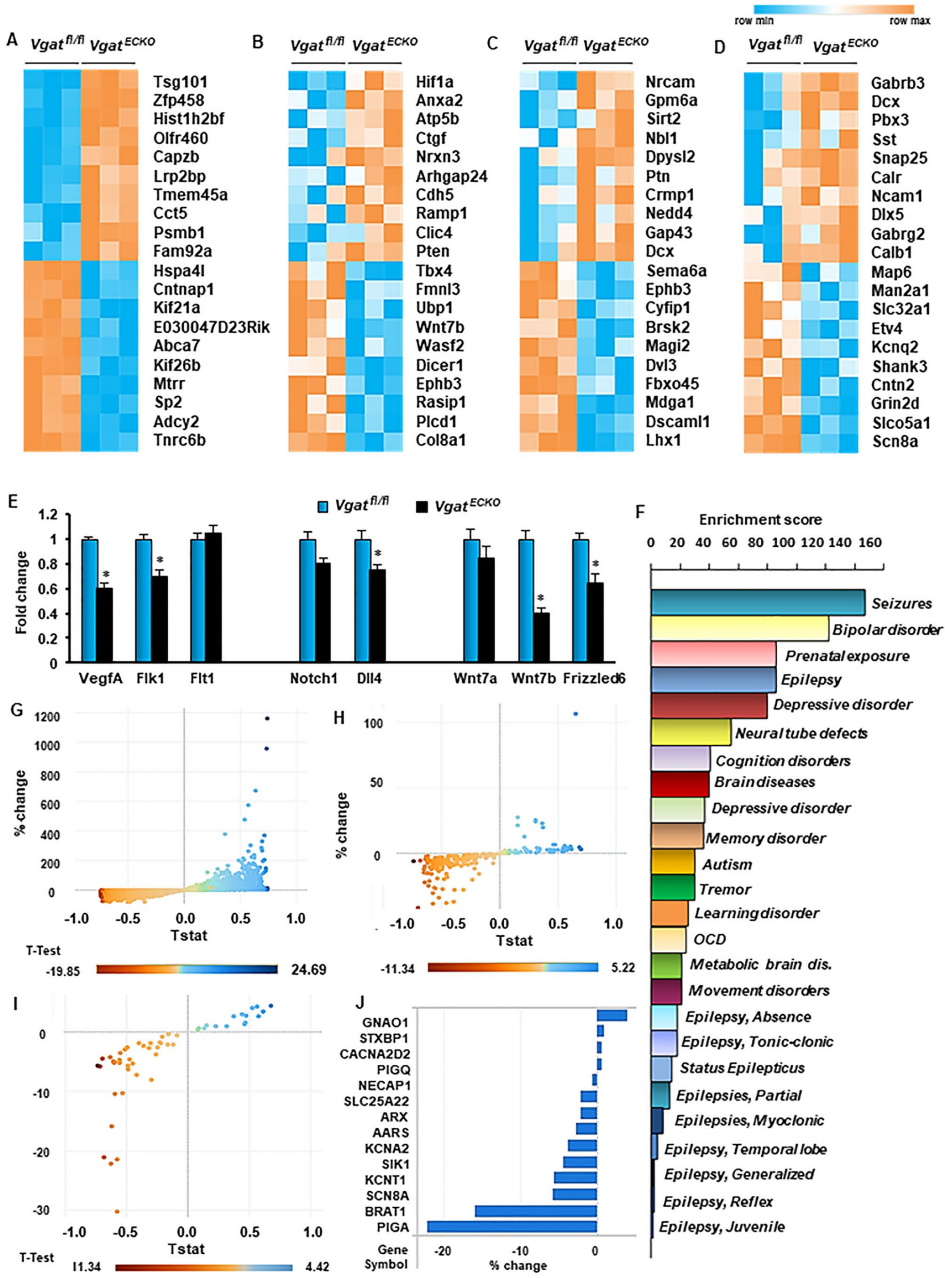
Embryonic telencephalic gene expression changes due to loss of endothelial GABA and consequent postnatal phenotype. **(A)** Heat map showing overall top 20 differentially expressed genes in *Vgat^ECKO^* versus *Vgat^fl/fl^* telencephalon (*n* = 3). **(B**-**D)** Heat maps were further classified to show top 20 differentially expressed genes in *Vgat^ECKO^* versus *Vgat^fl/fl^* telencephalon in three different categories: angiogenesis **(B)**, neurogenesis **(C)** and GABA neuronal development **(D)**. **(E)** Validation of altered expression of angiogenesis pathway genes in E15 *Vgat^fl/fl^* and *Vgat^ECKO^* periventricular endothelial cells by quantitative real-time PCR. **(F)** A classification of genes expressed in *Vgat^ECKO^* telencephalon using TPH1 CTD analysis shows enrichment in several neurological and psychiatric disease categories. Seizures and several different kinds of epilepsies were enriched in the list. **(G**-**I)** The scatter plots display values for each gene with signal present in tissue specimens. The percentage change in expression in *Vgat^ECKO^* samples compared to the WT and the Tstat associated with the comparison are indicated on the axes for all genes combined **(G)**, McTague only genes **(H)** and CDT genes associated with seizure conditions by marker/mechanism, marker/mechanism/therapeutic and therapeutic direct evidence **(I)**. The color of each mark indicates the *t*-test result for the comparison. **(J)** Graphical illustration of genes with percentage change in expression in *Vgat^ECKO^* telencephalon with respect to early infantile epileptic encephalopathy (isolated from^34^).

Our previous studies have implicated the importance of periventricular endothelial cells for a wide range of neuropsychiatric diseases^16^, therefore we questioned the significance of loss of the endothelial GABA source for global gene expression in the embryonic forebrain. We extracted RNA from *Vgat^fl/fl^* and *Vgat^ECKO^* whole telencephalon (E18), respectively; performed microarray hybridization on Mouse Gene 2.0 ST arrays (Affymetrix) and subsequent differential expression analysis. Two hundred and eighty six genes were differentially expressed (fold change cut off ≥ ± 50%) in *Vgat^ECKO^* versus *Vgat^fl/fl^* telencephalon of which the top 20 genes rated by significance have been depicted as a heat map (Figure 6A). Genes were further classified into three categories that are essential for embryonic forebrain development: angiogenesis (Figure 6B), neurogenesis (Figure 6C) and GABAergic neuronal development (Figure 6D). Top 20 differentially expressed genes in each category are shown. The gene expression profile revealed that loss of endothelial GABA has far reaching consequences for critical events during brain development and can modulate signaling events at the level of extracellular receptors, ion channels, transporters, intracellular signaling molecules as well as transcription factors (Figure 6A-6D). For instance, critical regulators of vascular morphogenesis and structure formation (*Wasf2*, *Rasip1*, *Fmnl3* and *Tbx4*) were downregulated in *Vgat^ECKO^* telencephalon (Figure 6B). Genes involved in cell proliferation, cell adhesion and cytoskeletal organization pathways were altered in *Vgat^ECKO^* telencephalon (either significantly upregulated or downregulated) when compared to controls (Figure 6A-6D). Since *Vgat* (*Slc32a1*) had been deleted from endothelial cells, the heat map showed decreased expression of *Slc32a1* in *Vgat^ECKO^* whole telencephalon as expected (Figure 6D). *Gabrb3*, on the other hand, was significantly upregulated in *Vgat^ECKO^* telencephalon (Figure 6D). Another interesting gene, *Shank3*, that is widely linked with ASD^32,33^ was significantly downregulated in *Vgat^ECKO^* telencephalon (Figure 6D).

What may be the signaling mechanisms perturbed by the loss of *Vgat* specifically in periventricular endothelial cells that contribute to the impaired telencephalic angiogenesis (Figure 4)? How does endothelial GABA interact with other signaling systems previously shown to regulate angiogenesis? To address these questions, we looked at three signaling pathways — VEGF signaling, Delta-Notch signaling and Wnt signaling, that are implicated in regulating diverse aspects of CNS angiogenesis. We examined expression of ligand/receptor combinations: vascular endothelial growth factor (isoform A; *Vegfa*) and its receptors *Flk1* and *Flt1*, *Dll4* and its receptor *Notch1*, Wnt signaling ligands (*Wnt7a* and *Wnt7b*) and receptor *Frizzled6*, specifically in periventricular endothelial cells, isolated from E15 *Vgat^fl/fl^* and *Vgat^ECKO^* telencephalons. There was a significant (*P* < 0.05) decrease in *Vegfa*, *Flk1*, *Dll4*, *Wnt7b* and *Frizzled6* mRNA (but not *Flt1, Notch1* and *Wnt7a* mRNA) in *Vgat^ECKO^* endothelial cells (Figure 6E). Since loss of endothelial *Vgat* altered the expression of molecules critical for telencephalic angiogenesis, our results suggest that other important angiogenesis signaling pathways may be either under direct control of or actively interacting with the endothelial GABA signaling pathway.

We next questioned whether the gene expression profile of *Vgat^ECKO^* embryonic telencephalon could be used to predict the postnatal phenotype of *Vgat^ECKO^* mice using the Comparative Toxicogenomic Database. When genes were classified according to disease categories, the genes expressed in *Vgat^ECKO^* telencephalon showed enrichment in neuropsychiatric disease categories like seizures, epilepsy, depression and autism (Figure 6F; Supplementary information, Figure S11). Several different types of epilepsies appeared in the list (Figure 6F). Epilepsy-related genes that were altered in *Vgat^ECKO^* telencephalon were isolated and grouped into specific categories, from the CTD database and from an elegant study^34^ describing the genetic landscape of early postnatal (infancy and childhood) epilepsies (Supplementary information, Table S1). Childhood epilepsies comprise many age-related epilepsy syndromes characterized by specific seizure types and neurological features and are a heterogeneous group of devastating disorders that cause developmental delays or regression^34^. Scatter plots depict the changes in epilepsy-related gene expression in *Vgat^ECKO^* telencephalon when compared to controls for all genes combined (Figure 6G), McTague only genes (Figure 6H) and genes isolated from the CTD database (Figure 6I). Specific genes that showed changes in expression in *Vgat^ECKO^* telencephalon, with respect to different groups of childhood epilepsies (isolated from^34^) have also been graphically illustrated (Figure 6J, Supplementary information, Figures S12, S13).

### Postnatal phenotype of *Vgat^ECKO^* mice

**Figure 7:**
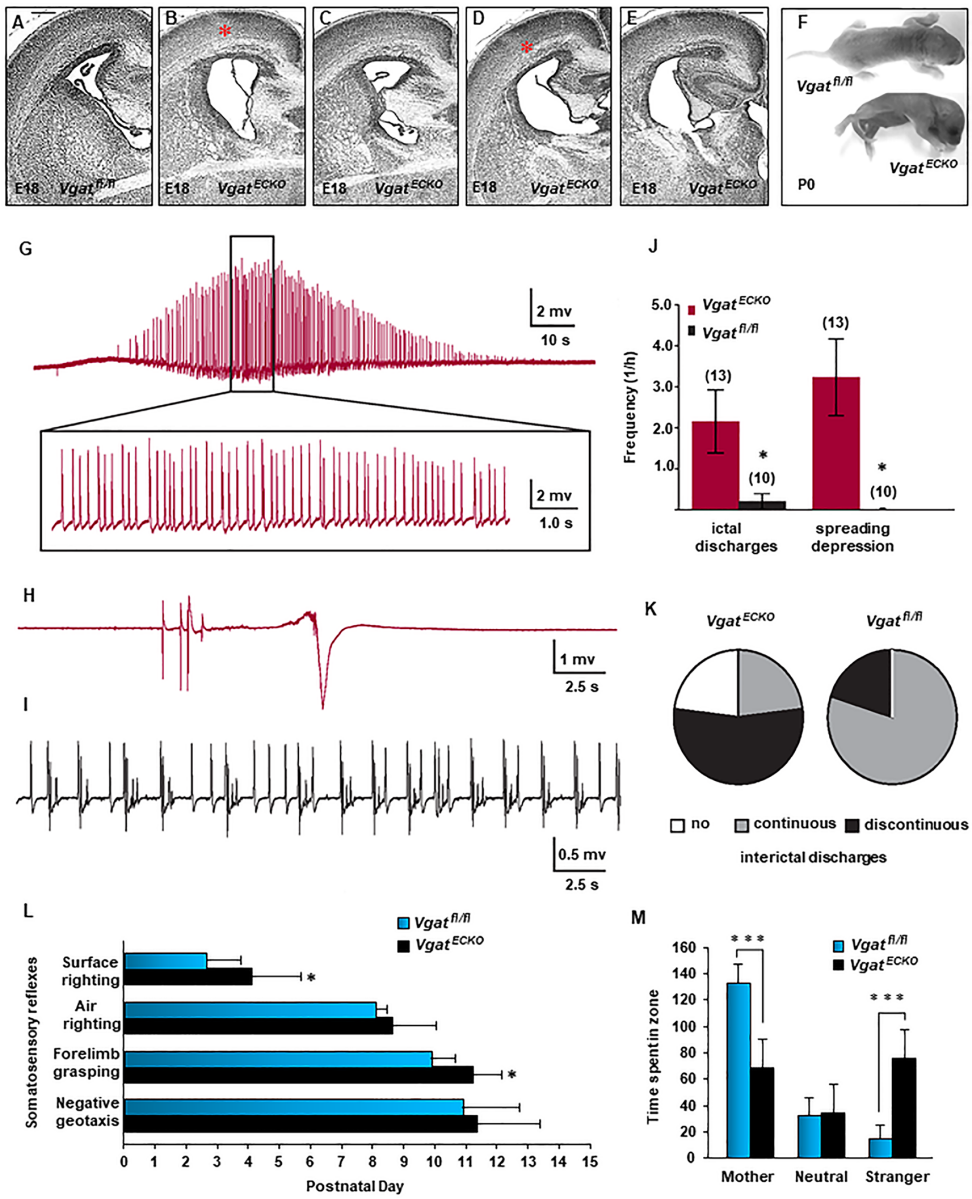
Postnatal phenotype of *Vgat^ECKO^* mice. **(A**-**E)** H&E staining revealed abnormal cellularity in *Vgat^ECKO^* cortical plate along the rostro caudal axis (red asterisks, **B**, **D**) when compared to *Vgat^fl/fl^* cortex **(A)**. Dilated and abnormal ventricles were observed in *Vgat^ECKO^* ventral telencephalon **(B**-**E)**. **(F)** *Vgat^ECKO^* pups were smaller in size at birth when compared to *Vgat^fl/fl^* pups. **(G)** Ictal activity in *Vgat^ECKO^* hippocampus (expanded with inset). **(H)** Spreading depression and preceding discontinuous interictal activity in *Vgat^ECKO^* hippocampus. **(I)** Continuous interictal activity in *Vgat^fl/fl^* hippocampus. **(J)** *Vgat^ECKO^* slices (*n* = 13) displayed ictal-type discharges significantly more frequently when compared to *Vgat^fl/fl^* slices (*n* = 10). *Vgat^ECKO^* slices (*n* = 13) exclusively showed repetitive spreading depression while control slices (*n* = 10) showed none (frequencies given as mean ± SEM, ^*^P < 0.05, Fisher's exact test). **(K)** Pie chart depicting proportions of slices displaying interictal discharges in *Vgat^ECKO^* and *Vgat^fl/fl^* slices (^*^P < 0.05, *χ*^2^-test). **(L)** Somatosensory reflexes — surface righting and forelimb grasping were significantly affected in *Vgat^ECKO^* mice; Data represent mean ± SD (*n* = 9, ^*^P < 0.05, Student's *t*-test). **(M)** *Vgat^ECKO^* mice showed significantly lower preference to maternal scent when compared to controls and instead spent longer time in the stranger's zone; Data represent mean ± SD (*n* = 9, ^*^P < 0.05, Student's *t*-test). Scale bars: **A**, 100 μm (applies to **B**-**E**).

No marked perturbation of cortical cytoarchitecture (e.g., heterotopias, dysplasia) was observed in *Vgat^ECKO^* telencephalon at late embryonic stage E18 (Figure 7A-7E). However, enlarged lateral ventricles were routinely seen (Figure 7B-7E), and abnormal cellularity in the CP was observed along the rostrocaudal axis (Figure 7B, 7D). Interestingly, the postnatal phenotype of *Vgat^ECKO^* mice turned out similar to the predictions offered by the prenatal gene expression signature. *Vgat^ECKO^* mice were smaller in size at birth than their floxed littermates (Figure 7F). *Vgat^ECKO^* mice started to show seizure-like activity between P7 and P14. Alterations in postnatal behavior were characterized by periods of quiescence, interrupted by tremors and a reduction in voluntary movement (Supplementary information, Movie S1). *Vgat^ECKO^* mice were fragile and died between P20 and P35. Since *Vgat^ECKO^* mice were unable to survive the surgical procedure for *in vivo* EEG recordings, we performed field potential recordings to measure the degree of hyperexcitability in hippocampal slices. *Vgat^ECKO^* slices displayed ictal-type discharges (Figure 7G, 7J), discontinuous interictal activity and spreading depression (SD), (Figure 7H, 7J) when compared to floxed controls that showed continuous interictal activity and no SD (Figure 7I, 7J). Control slices displayed ictal-type discharges with a significantly lower frequency than *Vgat^ECKO^* slices (Figure 7J). Furthermore, *Vgat^ECKO^* slices exclusively showed repetitive SDs (61.5% of slices), while control slices showed none (Figure 7J). *Vgat^ECKO^* slices showed no interictal discharges in 23% of the preparations, continuous interictal activity in another 23% and discontinuous interictal activity in 54% of the preparations (Figure 7K). On the other hand, controls showed discontinuous interictal activity in 20% of the preparations, and in 80% only continuous interictal activity (Figure 7K). The ictal type activity in *Vgat^ECKO^* slices is indicative of higher network excitability and epileptogenicity. Higher SD susceptibility has also been linked to higher epileptogenicity^35^. Furthermore, SD has been reported to trigger ictal-like activity *in vitro* (in rat brain slices)^36^, or enhance evoked activity^37^. Importantly, SD has also been shown to enhance excitability in human epileptic neocortex *in vitro*^38^. In conclusion, increased SD susceptibility is also a sign of increased excitability.

Developmental milestones of *Vgat^ECKO^* mice were significantly affected. Beginning on postnatal day 1 (PND 1), the mice were examined daily for acquisition of somatosensory reflexes and neurodevelopmental markers: surface righting, air righting, grasping and negative geotaxis. *Vgat^ECKO^* mice showed a deficit in surface righting and forelimb grasping (Figure 7L). Maternal scent preference, a test for social communication^39^ was conducted on PND 14 pups. While *Vgat^fl/fl^* mice spent significantly more time in the mother's bedding, indicative of social recognition of maternal scent when compared to the scent of a stranger female, *Vgat^ECKO^* mice showed significantly lower preference for maternal scent by comparison to the stranger's scent (Figure 7M). *Vgat^ECKO^* mice did not show signs of impaired movement capability in the maternal scent test. These data indicate that developmental milestones and social recognition are impaired in *Vgat^ECKO^* mice. Collectively, these results suggest that *Vgat^ECKO^* mice can serve as a model for infantile/childhood epilepsy or ASD.

### Marked vascular and interneuron deficits in the *Vgat^ECKO^* cerebral cortex

**Figure 8:**
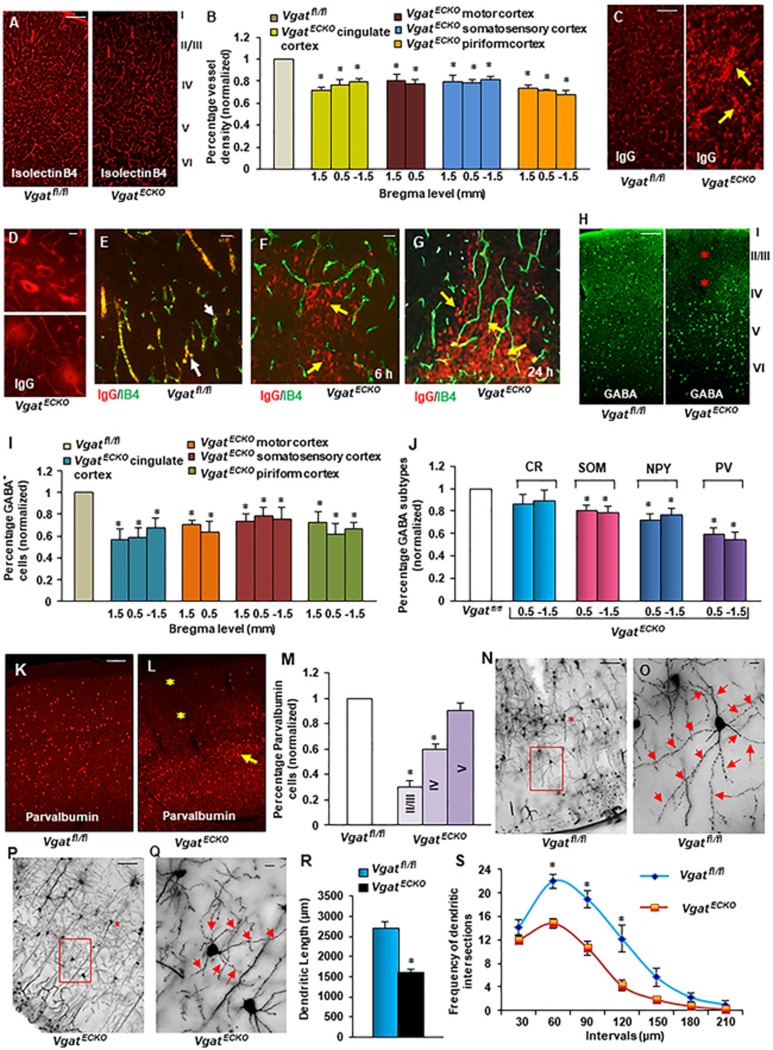
Postnatal consequences of loss of endothelial cell-derived GABA. **(A**, **B)** Significantly affected regions in *Vgat^ECKO^* brain at P30 were cingulate, motor, somatosensory and piriform cortex in which reductions in vessel density were observed. Images depict somatosensory cortex. *Vgat^ECKO^* data were normalized to *Vgat^fl/fl^* data; Data represent mean ± SD (*n* = 6, ^*^P < 0.05, Student's *t*-test). **(C**, **D)** Extravascular IgG staining was observed in P30 *Vgat^ECKO^* cerebral cortex (yellow arrows, **C**) and IgGs formed halos around microvessels **(D)**. **(E**-**G)** Co-labeling with isolectin B4 revealed that while IgGs are localized to vessels in *Vgat^fl/fl^* cortex **(E)**, IgG leakage and uptake by neurons were observed at various time points after status epilepticus (**F**, **G**; *n* = 8). **(H**, **I)** Concurrent reduction in GABA cells was observed in the cortical regions examined. The GABA cell distribution was very abnormal in *Vgat^ECKO^* with several cells clustered in layer IV-V and few to none in upper layers (red asterisks) indicative of cortical asynchrony. *Vgat^ECKO^* data normalized to *Vgat^fl/fl^* data **(I)**; Data represent mean ± SD (*n* = 6, ^*^P < 0.05, Student's *t*-test). **(J)** Numbers of calretinin^+^, somatostatin^+^, neuropeptide Y^+^ and parvalbumin^+^ subclasses in somatosensory cortex from P30 old mice. *Vgat^ECKO^* data normalized to *Vgat^fl/fl^* data **(J)**; Data represent mean ± SD (*n* = 10, ^*^P < 0.05, Student's *t*-test). **(K**, **L)** Parvalbumin immunoreactive cells in the *Vgat^ECKO^* somatosensory cortex **(L)** showed a similar abnormal profile as GABA immunoreactive cells **(H)**. Yellow asterisks in **(L)** point to significant reduction of parvalbumin^+^ cells in layers II/III and yellow arrow points to cells abnormally clustered in layer V. **(M)** Quantification of parvalbumin^+^ cells in *Vgat^fl/fl^* and *Vgat^ECKO^* somatosensory cortical layers; Data represent mean ± SD (*n* = 10, ^*^P < 0.05, Student's *t*-test). **(N**-**Q)** Representative images of the basket cells in the *Vgat^fl/fl^* **(N)** and *Vgat^ECKO^* **(P)** somatosensory cortex. Basket cells sampled (red boxes; **N**, **P**) were mainly located at the layer II-III close to neighboring pyramidal cells (red asterisks) of the somatosensory cortex. Higher magnification of the basket cell morphology was illustrated in **O** and **Q**. Compared to *Vgat^fl/fl^* cortex **(O)**, *Vgat^ECKO^* cortex showed a significant retraction of dendritic trees (red arrows in **Q**). **(R)** Comparison of dendritic length of *Vgat^fl/fl^* and *Vgat^ECKO^* basket cells. There was a 41% reduction of dendritic lengths of basket cells of the *Vgat^ECKO^* group when compared to the *Vgat^fl/fl^* group (*n* = 9, ^*^P < 0.05, ANOVA). **(S)** Comparison of frequency of dendritic intersections × 30-μm interval from the soma of basket cells between *Vgat^fl/fl^* and *Vgat^ECKO^* group. There was a significant reduction in the frequency of intersections at a distance of 60-120-μm from the soma of basket cells of the *Vgat^ECKO^* group (*n* = 9, ^*^P < 0.05, ANOVA and *post hoc* tests). Scale bars: **A**, 100 μm (applies to **C**, **H**, **K**, **L**, **N**, **P**), **E**, 50 μm (applies to **F**,**G**), **D**, 25 μm (applies to **O**, **Q**).

Cortical regions — cingulate, motor, somatosensory and piriform cortex in the *Vgat^ECKO^* brain at P30 were more significantly affected than in the *Gabrb3^ECKO^* brain, and reductions in vessel density were observed at all of the bregma levels examined (Figure 8A, 8B). To evaluate the putative damage of the BBB at P30, we looked for a leakage of serum IgGs using IHC. No IgG leakage was observed in *Vgat^fl/fl^* somatosensory cortex while on the other hand, extra-vascular IgGs were detected in *Vgat^ECKO^* somatosensory cortex (Figure 8C). IgGs formed halos with a concentration gradient around *Vgat^ECKO^* microvessels (Figure 8D). Next we performed a double fluorescence labeling for IgG and isolectin B4 on P30 *Vgat^fl/fl^* and *Vgat^ECKO^* sections. While immunohistochemical localization of IgG and isolectin B4 was observed in *Vgat^fl/fl^* vessels (Figure 8E), IgG leakage along with neuronal uptake was observed in *Vgat^ECKO^* somatosensory cortex, 6 or 24 h after limbic status epilepticus (Figure 8F, 8G) indicative of a rapid BBB impairment.

Concurrent reductions in GABAergic interneurons was observed in cingulate, motor, somatosensory and piriform cortex (Figure 8H-8I). Layer-specific loss of GABAergic interneurons along with abnormal GABAergic (Figure 8H) and glutamatergic neuronal distribution (Supplementary information, Figure S14A, S14B) was marked in *Vgat^ECKO^* cerebral cortex, which is indicative of an asynchronous cortical circuitry. Activation of caspase 3 was insignificant in *Vgat^ECKO^* cortex, further demonstrating that the GABA cell deficits were not due to GABAergic neuronal cell death (Supplementary information, Figure S14C-S14E). We also tested whether specific subtypes of GABAergic interneurons were affected in *Vgat^ECKO^* cerebral cortex. Our results showed that while the calretinin population was not affected, somatostatin^+^ and NPY^+^ neurons were reduced, but the most significantly affected population was the parvalbumin^+^ neurons (Figure 8J). This is particularly interesting since parvalbumin^+^ neurons account for ∼50% of the GABAergic interneurons in the rodent cortex^40^. The parvalbumin subpopulation seems to be markedly affected in epilepsy and ASDs, possibly because these cells are less proficient at inhibiting pyramidal cells. In *Vgat^ECKO^* mice, similar to the GABAergic interneuron profile (Figure 8H), parvalbumin^+^ neurons also showed abnormal distribution and layer specific reductions in somatosensory cortex. Cells appeared to be stuck and clustered in lower layers (layer V) in *Vgat^ECKO^* cortex, while layers II/III showed the most significant reduction (Figure 8K-8M).

Parvalbumin^+^ interneuron class comprises of basket cells that focus on targeting the somata and proximal dendrites of pyramidal neurons and interneurons, and this gives them the unique advantage to regulate the gain of the integrated synaptic response. Large basket cells are classic basket cells with extensive axonal arborizations that can inhibit neurons in lower and upper layers and in neighboring and distant columns^40^. Golgi impregnation and morphological analysis showed differences in axonal arborizations of large basket cells in layer II/III from *Vgat^ECKO^* somatosensory cortex when compared to *Vgat^fl/fl^* cortex (Figure 8N-8Q, Supplementary information, Figure S15A, S15B). While *Vgat^fl/fl^* basket cells had long horizontally and vertically projecting axon collaterals that arborized on and around somata of target neurons, *Vgat^ECKO^* basket cells had shorter-range axon collaterals that failed to do so. There was a significant reduction in total dendritic lengths of basket cells (41%) in *Vgat^ECKO^* somatosensory cortex (Figure 8Q, 8R). *Vgat^ECKO^* basket cells also showed a reduction in the number of dendritic segments (37%) as compared to controls. By comparing the dendritic length versus branch order, *Vgat^ECKO^* basket cells showed a significant reduction in dendritic length in the middle portion, the 4th and 5th branch orders of the dendritic tree (Supplementary information, Figure S15C). To further investigate dentritic morphology of SS1 basket cells, Sholl analysis was performed in order to characterize the morphological changes in reference to a series of concentric circles (spheres in 3D) around the soma of the basket cell. Frequency of (dendritic) intersections at a 30-μm interval from the soma of basket cells between *Vgat^fl/fl^* and *Vgat^ECKO^* groups was determined. The *Vgat^ECKO^* group exhibited a significant reduction in the frequency of intersections in the middle portion (60-120 μm) of the dendritic field (Figure 8S). Also, *Vgat^ECKO^* basket cells showed a reduction of dendritic lengths in the middle portion (90-120 μm) of the dendritic field (Supplementary information, Figure S15D). These results illustrate significant alterations in the dendritic morphology of basket cells of *Vgat^ECKO^* somatosensory cortex. It signifies the importance of endothelial cell-derived GABA for normal formation and synchronization of the cortical microcircuitry.

## Discussion

Radial glia, which are cortical neural stem cells, cortical progenitors and migrating GABA neurons have all been reported to express functional GABA_A_ and GABA_B_ receptors early in development that respond to ambient GABA levels in many different ways to trigger several important events during cerebral cortex development. GABA has been well established as the first excitatory transmitter to become functional in the embryonic brain, acting as an epigenetic factor to control processes like neural progenitor proliferation, neuronal migration, dendritic maturation and synaptogenesis, and is a key player in building the cortical network^8,9,10^. Specifically, during the tangential neuronal migration process, neocortical GABAergic interneurons progressively acquire responsiveness to GABA; the paracrine actions of GABA acting on several receptor subtypes, being the key motility promoting signal. The functional expression of GABA_A_ receptor subunits in tangentially migrating interneurons derived from the MGE has recently been characterized^41^. While early migrating interneurons express alpha 2 and alpha 3 subunits when they are at the corticostriate junction, they additionally upregulate alpha 1 and gamma 1-3 subunits as soon as they enter the developing cortex and the functional implications of this upregulation of multiple GABA_A_ receptor isoforms with higher affinity to GABA in the migration process is not known^41^. Within the cortex, GABA's complexity increases and it has been reported to play contrasting roles on migrating neurons by acting as a 'GO' signal in lower layers and as a 'STOP' signal in upper cortical layers^42^. These multiple actions of GABA exerted at different developmental stages all appear to be mediated through a paracrine, diffuse, non-synaptic mode of action. However, current views of neocortical development have depicted this source of GABA during corticogenesis to be exclusively neuronal. And given its multiple roles in cerebral cortex development, GABA has been epitomized as a deeply interesting and versatile molecule.

We believe that the cell-type specific source that secretes GABA in the embryonic forebrain is the key to its versatility. Neuronal GABA seems to be sufficient to some degree for telencephalic neurogenesis in the absence of endothelial GABA. A previous study has demonstrated that GABA promotes VZ cell division while inhibiting SVZ cell divisions^21^. Our study also shows differences in cell proliferation in the absence of endothelial GABA in the VZ versus SVZ. The increase in cells undergoing mitosis in the SVZ of *Vgat^ECKO^* telencephalon suggests that endothelial GABA functions to inhibit cell proliferation here, while neuronal GABA is sufficient for VZ cell proliferation. Also, while possible paracrine roles of neuronal GABA on angiogenesis cannot be overlooked, it was clearly unable to rescue the vascular defects in the absence of endothelial GABA. Our results show that endothelial GABA is indispensable for angiogenesis and GABAergic neuronal tangential migration in the embryonic telencephalon. Neuronal GABA cannot compensate for these unique roles of endothelial GABA. Endothelial GABA may be a requisite for fostering neurovascular interactions in the long term. For instance, if GABA coming from neurons can contribute to GABA_A_ receptor-mediated GABA release from endothelial cells, then it is possible that loss of this chemotactic signal during development due to stalled and abnormal neuronal migration as observed in *Vgat^ECKO^* telencephalon, may in turn have affected angiogenesis. Thus, the downstream cellular mechanism of endothelial GABA-mediated regulation is its key effect on neuronal migration that is highly concentration, time and location dependent.

Endothelial GABA closely influences key angiogenesis signaling pathways — VEGF, Delta-Notch and Wnt signaling, that play important roles in endothelial cell proliferation, migration, sprouting, vascular pattern formation and induction of BBB properties. The marked impairment of telencephalic angiogenesis that lead to BBB defects in *Vgat^ECKO^* mice may be due to direct links between endothelial GABA and BBB function, for instance, regulation of tight junction protein expression by GABA. Since our study has identified the relevant ligand/receptor pairs, it will facilitate future mechanistic studies linking endothelial GABA signaling with specific angiogenesis processes. The stalled GABAergic neuronal migration and accumulation in the ventral telencephalon due to absence of endothelial GABA also highlights the importance of endothelial versus neuronal GABA for subtype-specific contributions to the cerebral cortex. Parvalbumin^+^ neurons derive almost entirely from the MGE, whereas contributions from both MGE and CGE have been reported for somatostatin^+^, NPY^+^ and CR^+^ neurons^43^. The marked reduction of the parvalbumin^+^ population in *Vgat^ECKO^* mice highlights the important contribution of endothelial GABA for MGE-derived GABAergic neuronal migration. Interestingly, susceptibility of the parvalbumin population has been reported in neuropsychiatric diseases like schizophrenia, epilepsy and ASD^2,44^. Though GABAergic cell death was not significant in *Gabrb3^ECKO^* and *Vgat^ECKO^* brains, we cannot rule out possible contributions of reactive astrocytosis and microglial activation for some of the adult phenotypes.

**Figure 9:**
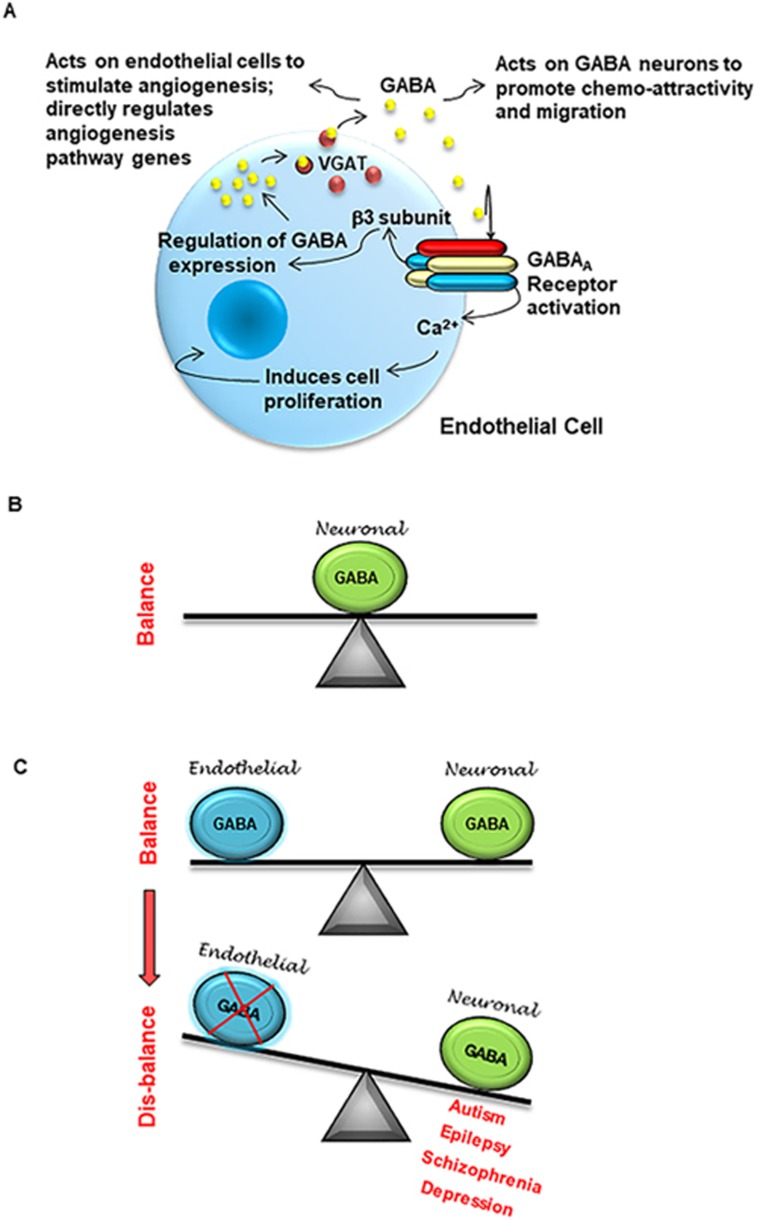
The significance of endothelial cell-derived GABA for brain development. **(A)** Summary schema depicting a novel positive feedback GABA signaling pathway in telencephalic endothelial cells. Endothelial GABA activates GABA_A_ receptors, triggering Ca^2+^ influx and endothelial cell proliferation. Endothelial GABA_A_ receptor β3 subunit can regulate GABA expression. VGAT is the primary mechanism for GABA release from telencephalic endothelial cells. Endothelial GABA release is essential for both angiogenesis and GABAergic neuronal migration in the embryonic telencephalon. **(B)** Current concepts depict the source of GABA in the embryonic telencephalon as neuronal. **(C)** Our studies show that the GABA balance in the embryonic telencephalon is maintained by both endothelial cells and neurons. Neuronal GABA cannot compensate for the loss of endothelial GABA. Tipping the balance to cause partial or complete loss of endothelial GABA can result in a spectrum of neuropsychiatric diseases such as autism, epilepsy, schizophrenia, anxiety and depression.

Since development of the periventricular vascular gradient precedes neuronal migration during embryonic development^11,16^, we think that the mechanism of GABA release from periventricular endothelial cells is constitutive at first, then facilitated by GABA itself. GABA-mediated activation of endothelial GABA_A_ receptors triggers an increase in intracellular Ca^2+^ that in turn induces endothelial cell proliferation. Endothelial GABA_A_ receptor beta 3 subunit is not only essential for GABA_A_ receptor functions, but also modulates GABA expression, resulting in lowered GABA levels in *Gabrb3^ECKO^* mice during embryonic development. This is of interest, since silencing GABA_A_ receptor subunits alters GABA expression and release^16^ and correlations between lowered GABA levels and altered GABA_A_ receptors have been reported in many neuropsychiatric disease scenarios^1,45,46,47,48,49^. In addition, our studies show that *Vgat* is the primary mechanism for GABA release from endothelial cells in the embryonic telencephalon, since loss of endothelial *Vgat* completely abolished endothelial GABA secretion. Our experiments thus point to a novel positive feedback of GABA release that is essential for telencephalic angiogenesis (Figure 9A). Since GABA itself promotes the developmental switch from excitatory during prenatal development to inhibitory at birth^50,51,52^, it would be interesting for future studies to dissect the specific roles of the endothelial GABA signaling pathway and novel mechanisms of action in the postnatal and adult brain.

Disturbances in vessel function, BBB and blood flow have repeatedly been observed in patients with epilepsy, ASDs, anxiety, depression and schizophrenia using old and new technologies^53,54,55,56,57^. However, these disturbances are usually linked to inflammation, changes in neural plasticity or seizure frequency. Our studies provide a direct cause for change in vessel function in psychiatric disorders that originates from intrinsic defects in vessels from the earliest developmental points. It illustrates the importance of a new endothelial GABA signaling pathway that molds neuronal development making lasting changes to cortical circuits and most importantly is sufficient to cause behavioral dysfunction. Our study also introduces the novel concept that variation in endothelial GABA levels during embryonic brain development can contribute to diversity in psychiatric disease symptoms.

We suggest re-consideration of current concepts that depict GABA signaling during brain development as predominantly neuronal (Figure 9B). The GABA balance in the embryonic telencephalon is maintained by both endothelial cells and neuronal cells (Figure 9C). Tipping the balance by either reducing or eliminating the endothelial GABA source can result in a spectrum of neuropsychiatric diseases such as autism, epilepsy, anxiety, depression or schizophrenia. These findings establish novel autonomous links between blood vessels and the origin of neuropsychiatric disease. Additionally, a role for modulation of vascular/endothelial GABA_A_ receptors emerges as a contributing factor for neuropsychiatric disease origin. Many tranquilizers, sedatives, anesthetics and anti-epileptic drugs used in obstetric medicine modulate GABA_A_ receptor-GABA function. Such treatments during pregnancy may cause problems in developing fetuses. Also, it is possible that altered GABA_A_ receptor expression, altered NKCC1/KCC2 expression and altered chloride concentrations in telencephalic endothelial cells may have direct consequences for angiogenesis and neuronal migration. A 'GABA therapy' might hold significant promise in some cases for the prenatal treatment or prevention of neuropsychiatric diseases. The clinical applications of angiogenesis today benefits millions of patients with cancer, blinding eye diseases, stroke and neurodegeneration^58^; in a similar way we expect this study to open new doors and accelerate innovative angiogenesis-mediated therapies for neuropsychiatric diseases.

## Materials and Methods

### Animals

Timed pregnant CD1 mice were purchased from Charles River laboratories, MA. Colonies of GAD65-GFP and Tie2-GFP mice were maintained in our institutional animal facility. *Tie2-cre* mice, *Gabrb3 floxed* (*Gabrb3^fl/fl^*) mice and *Vgat floxed* (*Vgat^fl/fl^*) mice were obtained from Jackson Labs. The *Tie2-cre* transgene is known for uniform expression of cre-recombinase in endothelial cells during embryogenesis and adulthood^59,60,61^. To selectively delete *Gabrb3* or *Vgat* in endothelial cells, *Tie2-cre* transgenic mice (males) were crossed to *Gabrb3^fl/fl^* mice (females) to generate *Tie2-cre; Gabrb3^fl^*^/+^ mice (males) or crossed to *Vgat^fl/fl^* mice (females) to generate *Tie2-cre; Vgat^fl/+^* mice (males). These were further crossed with *Gabrb3^fl/fl^* mice (females) or *Vgat^fl/fl^* mice (females) to obtain the *Gabrb3* and *Vgat* conditional knock-outs (*Tie2-cre; Gabrb3^fl/fl^* mice or *Tie2-cre; Vgat^fl/fl^* mice). The day of plug discovery was designated as embryonic day 0 (E0). Animal experiments were in full compliance with the NIH Guide for Care and Use of Laboratory Animals and were approved by the McLean Institutional Animal Care Committee.

### Histology, immunohistochemistry and microscopic analysis

Paraffin IHC and fixed slice IHC was performed on embryonic brains, while frozen section IHC was used on postnatal brains. Briefly, for paraffin IHC — E13, E15 and E17 brains were fixed in zinc fixative (BD Biosciences Pharmingen) for 24 h and processed for paraffin histology. Histological stainings with hematoxylin (Vector Laboratories) and eosin (Sigma) were performed on 8 μm coronal sections. Lectin histochemistry (with biotinylated isolectin B4, 1:50, Sigma) as well as IHC was performed on 20 μm sections. Primary antibodies used for IHC were as follows: anti-CD31/PECAM-1 (1:50, BD Biosciences Pharmingen), anti-GAD65/67 (1:50, Millipore), anti-GABRB3 (1:50; Sigma), anti-VGAT (1:100, Synaptic Systems), anti-PHH3 (1:200, Millipore), anti-MAP2 (1:50, Sigma), anti-TBR1 and TBR2 (1:100, Abcam), anti-Ki67 (1:30, Sigma) and anti-TH (1:200, Millipore) followed by secondary detection with AlexaFluor conjugates (Invitrogen). DAPI (Vector Laboratories) was used to label nuclei. For slice preparations and IHC, brains from E13 and E15 embryos were collected and fixed in 4% paraformaldehyde at 4°C. Vibratome slices (50 μm) were prepared and incubated in anti-biotinylated isolectin B4 (1:40, Sigma) with 1% TritonX-100 at 4 ^ο^C overnight. After six washes in phosphate buffered saline (PBS), slices were incubated with secondary antibody (Alexa 594 streptavidin conjugate) for 6 h at 4 ^ο^C, washed and mounted. For frozen section IHC, P30 and P90 brains were removed, fixed in 4% PFA for 24 h, cryo-protected in sucrose gradient, embedded into frozen blocks; sectioned at 40 μm on a cryostat and immunostained with anti-isolectin B4 (1:50, Sigma), anti-GABA (1:400, Sigma), anti-calretinin (1:200; Swant), anti-somatostatin (1:2 000; Bachem), anti-NPY (1:1 000, Millipore), anti-parvalbumin (1:200, Immunostar), anti-caspase (1:200, Millipore) and anti-VGLUT1 (1:200, Synaptic Systems) antibodies. Twenty sections from each brain were used for IHC and histology experiments. Uniform penetration of antibodies or stains throughout the section was ascertained and quality of the staining in each digital section was examined. Only those sections which showed uniform labeling were included in further analysis. All low- and high-magnification images were obtained from an FSX100 microscope (Olympus).

### Morphometry

A stereological point grid was superimposed on digital images of biotinylated isolectin-B4^+^ vessels using ImageJ software. The ratio between points falling on blood vessels and on brain tissue was calculated for each section, and average values were obtained for four specific cortical regions: cingulate, somatosensory and piriform (at bregma levels 1.5, 0.5 and −1.5), and motor (at bregma levels (1.5 and 0.5) using stereotaxic coordinates^62^.

### Cell counting

Profiles of GABA^+^ immunoreactive cells were counted in the four areas of cortex: cingulate, somatosensory and piriform (at bregma levels 1.5, 0.5 and −1.5), and motor (at bregma levels (1.5, 0.5) using stereotaxic coordinates^62^. Profiles of GABA subtype immunoreactive cells were counted in the somatosensory cortex (at bregma level 0.5). For each area, cells in the strip of cortex from the pial surface to the white-gray matter interface was counted using ImageJ software and plotted. Details of sample size evaluation are provided in 'statistical analysis' and sample sizes are provided in figure legends.

### Lectin perfusion and immunohistochemistry

Prior to killing and tissue harvest, animals were injected retroorbitally with 100 μl Fluorescein-labeled Lycopersicon Esculentum (Tomato) Lectin solution (Vector laboratories) over 2 min. About 5 min after completion of the lectin injection, whole mouse brains were harvested after intracardiac perfusion with 4% paraformaldehyde in PBS. Tissue was then fixed overnight in 4% paraformaldehyde/PBS, embedded in paraffin, and mounted on glass slides in 10-μm thick sections. Prior to IHC, tissue was deparaffinized and antigen retrieval was performed in a pH 9 solution (DAKO) at 96 °C. To prevent non-specific staining, sections were incubated with 5% normal donkey serum in PBS prior to incubation with an anti-CD31 mAb (1:20, Dianova), followed by secondary detection with a fluorescent antibody (Jackson ImmunoResearch). All image analyses were performed using ImageJ software. In brief, CD31-positive images were thresholded using an automatic ImageJ thresholding function, binary images generated, and CD31^+^ vessel ROIs (region of interests) generated. For MVD measurements, this number was correlated with the analyzed tissue area to compute micro vessel density. Vessel diameter was analyzed by selectively measuring the Feret's diameter in only elongated vessels. For lectin measurements^63^, the CD31 ROIs were overlayed over binary images from the lectin channel. The area percentage of the lectin positive area in each vessel ROI was measured and the average lectin area per vessel was plotted using the prism software.

### Isolation and primary culture of endothelial cells

Embryonic brains were dissected under a stereo microscope and the telencephalon was removed. Pial membranes were peeled out and pooled (pial endothelial cells). The remaining telencephalon without pial membranes was pooled as well (periventricular endothelial cells). Mesencephalon and metencephalon were combined to prepare control endothelial cells. Purity of endothelial cell cultures was established with endothelial cell markers and determined to be 100%^11,27^. Isolation and culture of endothelial cells of the three sets were performed according to published methodology^11,27^.

### Endothelial cell stainings, transwell assay, long-distance cell migration and chemoattraction assays

Periventricular endothelial cells were prepared from CD1 (wild type), Tie2-GFP, *Gabrb3^fl/fl^*, *Gabrb3^ECKO^*, *Vgat^fl/fl^* and *Vgat^ECKO^* embryos. Endothelial cells were labeled with the following primary antibodies: anti-biotinylated isolectin B4 (1:40, Sigma), anti-GABA (1:400, Sigma), anti-GABRB3 (1:200; Sigma), anti-VGAT (1:200, Millipore), anti-KCC2 (1:400, Sigma), anti-NKCC1 (1:400, Millipore), anti-GAD65/67 (1:400, Millipore) followed by secondary detection with AlexaFluor conjugates (Invitrogen). DAPI (Invitrogen) was used to label nuclei. Images were taken on an FSX100 microscope (Olympus). One million cells were examined for each IHC condition.

**Transwell assay** Endothelial cell migration was evaluated in 24-well transwell chambers (8-μm pore size; Corning, Lowell, MA, USA). The cells were labeled with Qdot nanocrystals (Thermo Fisher Scientific) and seeded into the upper chamber at 5 × 10^5^ cells/well. The bottom chamber was filled with serum-supplemented endothelial cell culture medium to serve as a chemoattractant. Cells were incubated at 37 °C for 24 h in a humidified incubator containing 5% CO_2_. Cells that migrated through the membrane were fixed, imaged and the number of cells from five different fields (upper, lower, right, left and center) of view was quantified to get an average sum of cells that migrated through the membrane and quantified.

**Long-distance cell migration assay** In preparation for cell migration assays, square culture inserts (ibidi GmbH) were placed along the entire diameter of a 35 mm dish to create a long track. Cultures of endothelial cells were plated throughout the track while E15 GE-derived GAD65-GFP^+^ neurons were plated at one end of the track. Endothelial cells were labeled with cell trace marker (CellLight Plasma Membrane-RFP, BacMam 2.0, Invitrogen) to visualize endothelial cell morphologies during subsequent imaging. The co-culture was maintained for 24 h in FCS-DMEM (Invitrogen). The migration of neurons on endothelial cells from one end of the dish to the other spanning a distance of 3.5 cm was imaged and quantified.

**Chemoattraction assay** To prepare explants for chemoattraction assays, the GE region was micro-dissected from GAD65-GFP^+^ve brains and further trimmed into blocks of equal size, respectively. Individual explants were plated on *Vgat^fl/fl^* or *Vgat^ECKO^* periventricular endothelial cell cultures labeled with CellLight Plasma Membrane-RFP (Invitrogen). The co-culture was maintained for 24 h in FCS-DMEM (Invitrogen). The chemoattractive responses of GFP^+^ve cells from the explant toward endothelial cells in each experiment were imaged and analyzed.

### BBB assessment

Immunofluorescence for claudin 5 and IgG were performed on 40 μm cryo sections with anti-claudin 5 (1:200, Thermo Fisher), anti-mouse IgG antibody (1:200, Molecular Probes) and anti-ZO1 (1:400, Thermo Fisher), respectively. For double-fluorescence labeling for IgG and isolectin B4, cryo sections (10 μm) were first incubated with anti-biotinylated isolectin B4 antibody (1:100, Sigma) at room temperature for 1 h. Subsequently, they were incubated with secondary antibody, streptavidin-conjugated Alexa 488 at room temperature for 30 min. After being washed in PBS, they were then incubated with goat anti-mouse IgG antibody coupled to Alexa Fluor 594 (Molecular Probes) at a dilution of 1:200 for 30 min. Finally, they were examined in an FSX100 microscope. Trans-cardiac perfusion of E18 embryos was performed with 3 kDa biotinylated dextran (0.15 mg/ml, Invitrogen), tissue sections were stained with streptavidin Alexa 594 and fluorescence in tissue sections was quantified by ImageJ software. Perfusions and analysis was done blinded to genotype.

### *In vivo* bromodeoxyuridine labeling

A single BrdU injection (50 μg/g) was administered to pregnant dams carrying E13 mice. Embryonic brains were removed at E17, immersed in zinc fixative (BD Biosciences Pharmingen) for 24 h, and processed for paraffin wax histology. BrdU IHC (fluorescence and DAB) was performed on coronal, 8 μm sections. Double labeling was performed with a mouse monoclonal antibody to BrdU (1:75, BD Biosciences Pharmingen) and a rabbit polyclonal antibody to LHX6 (1:100, Sigma).

### Behavioral experiments

Mice were housed in our animal facility with a 12 h light cycle with *ad libitum* access to food and water. Offspring stayed with their mothers until weaning at PND 21 after which males and females were separated. Before all behavioral testing, mice were acclimated to the testing room for 1 h. Behavioral assays were performed according to established protocols referenced here: nest building with nestlets^64^, nest building with shredded paper^65^, self-grooming^66^, light-dark box^22^, tail suspension test^67^, tube dominance test^68^, three chamber social interaction test^69^ and buried food test^70^. Both males and females were used for all behavioral assays. Developmental milestones and social recognition tests were conducted with several cohorts of pre-weanling pups. Experimenters scoring behaviors were blinded to the genotypes. Sample sizes for each assay are noted in figure legends.

### Electrophysiological recordings, calcium imaging and BrdU assay

Cultured mouse E15 periventricular endothelial cells and cortical neuronal cells plated on 10 mm glass cover slips were placed inside a 35 mm culture dish with cover glass bottom (WPI FD35 FluoroDish) and continuously perfused with an extracellular solution at a rate of 2 ml/min. The extracellular solution contained 150 mM NaCl, 2.5 mM KCl, 2 mM CaCl_2_, 10 mM HEPES, 10 mM glucose, pH 7.3. Membrane currents were recorded by whole-cell configuration of the patch clamp technique using a List EPC7 amplifier (Medical System), at room temperature and at a holding potential of −70 mV. The intracellular solution used contained 140 mM potassium gluconate, 10 mM NaCl, 2 mM MgCl_2_, 10 mM HEPES, 1 mM EGTA, 4 mM Mg-ATP, 0.3 mM Na-GTP, pH 7.3. With this set of recording solutions, the chloride reversal potential (E-Cl^−^) was −61.8 mV at 24 °C. Data were filtered digitally at 2 KHz and acquired at 5 KHz by an Axon Instrument digitizer (Digidata 1322B) with pClamp 9 software using a Dell computer. Muscimol (Sigma) was applied with a puffer pipette (∼1 μm pore diameter) close to the cell (∼20 μm) by pressure ejection with a Picospritzer (General Valve). BMI (Sigma) was applied by bath perfusion. Drugs were kept at −20° as concentrated stock solutions and diluted on the day of the experiment.

For Ca^2+^ assays, E15 periventricular endothelial cells (1 million cells per assay) were incubated with Ca^2+^ indicator dye FluoForte AM according to manufacturer's instructions (Enzo Life Sciences), loaded into the chamber of an FSX100 microscope and imaged continuously before and after muscimol application. Fluorescence micrographs were digitalized and results were expressed as change in fluorescence over baseline fluorescence.

To test for cell proliferation, E15 periventricular endothelial cells (1 million cells per experiment) were incubated in the presence of the mitotic marker 5-bromo-2′-deoxyuridine (0.05% BrdU) for 1 h with or without muscimol to examine the impact on proliferation of these cells and processed for BrdU IHC.

### Hippocampal slice recordings

Procedures for recordings were as described earlier^71^. In brief, mice were anesthetized with diethylether, and decapitated. The brain was gently removed and shortly immersed in ice-cold dissection solution (containing, in mM: 125 NaCl, 26 NaHCO_3_, 3 KCl, 1.25 NaH_2_PO_4_, 0.2 CaCl_2_, 5 MgCl_2_ and 13 glucose). All solutions were equilibrated with 5% CO_2_/95% O_2_ to yield a pH of 7.4. Horizontal slices of ventral hippocampus (400 μm) were cut on a vibratome (Leica) and stored in a chamber filled with artificial cerebrospinal fluid (ACSF) containing (in mM) 125 NaCl, 26 NaHCO_3_, 3 KCl, 1.25, NaH_2_PO_4_, 2.5 CaCl_2_, 1.3 MgCl_2_ and 13 glucose. Then, slices were gradually brought to room temperature and allowed to recover for at least 60 min before recording. Generally, 2-4 slices per animal were used. For electrophysiological recordings, slices were transferred to an interface recording chamber perfused with ACSF (at 32 °C). Field potential recordings were obtained from the stratum radiatum of CA1 subfield using glass micropipettes (1 MΩ) filled with ACSF (DC recording, filtered at 2 kHz using EXT 10C amplifier, npi, Tamm, Germany). Epileptiform activity was induced by replacing ACSF with a bath solution containing 6 mM KCl and 0 mM MgCl_2_ (the remainder was composed as above).

### Gene expression profile analysis

RNA was extracted from E18 *Vgat^fl/fl^* and *Vgat^ECKO^* telencephalon using the PicoPure RNA Isolation Kit (Arcturus). RNA quality was determined and microarray hybridization was performed on Mouse Gene ST-2.0 gene chips (Affymetrix) at the Dana Farber Cancer Institute, Molecular Biology Core Facilities, Boston, MA, USA. For group comparison heat maps, expression levels were normalized with the SCAN method. Heat map visualization was conducted using Morpheus (Broad Institute, Boston, MA, USA) and ranked by *t*-test statistics. For gene network and CDT analysis, expression levels were normalized with the RMS method. The AltAnalyze pipeline was used to perform the Go-Elite analysis with 1.5-fold expression and 0.1 Fischer exact test as threshold parameters. CDT visualization was composed using Tableau 9.0 (Tableau, Seattle, WA, USA). MTRR CTD analysis and TPH1 CTD analysis were performed to classify genes according to disease categories. The analysis was performed according to established methodology: AltAnalyze^72^, SCAN^73^, RMS^74^ and CDT^75^.

### Real-time PCR

RT was performed by using transcriptor first-strand cDNA Synthesis Kit (Roche Diagnostic). PCR reactions were run on an ABI Prism 7500 (Applied Biosystems) sequence detection system platform. Taqman primers with 6-carboxyfluorescein probe for *VegfA*, *Flk1*, *Flt1*, *Notch1*, *Dll4*, *Wnt7a*, *Wnt7b* and *Frizzled6* were obtained from Applied Biosystems. The house keeping gene β2 microglobulin was used as a control. The relative gene expression among different samples and subsequent fold increase in periventricular versus pial endothelial cells was determined according to published methodology^76^.

### ELISA

Periventricular endothelial cells were prepared and seeded in 12 well culture plates at 0.1 × 10^6^ cells/well. Supernatants from endothelial cell cultures were collected after 96 h and stored at −80 °C for ELISA. GABA concentrations were quantitatively determined by competitive ELISA according to manufacturer's protocol (GABA Research ELISA Kits, Labor Diagnostica Nord, Germany), and absorbance was measured using a multiplate microplate fluorescence reader (Molecular Devices, CA, USA) at 450 nm.

### Golgi impregnation and morphological analysis

*Vgat^fl/fl^* and *Vgat^ECKO^* brains (P25) were shipped to Neurodigitech for Golgi impregnation. Serial coronal sections (120-μm thickness) were prepared that covered the anterior-to-posterior axis of the cerebral cortex. The somatosensory cortex was analyzed using stereology-based software (NeuroLucida, v10, Microbrightfield, VT), installed on a Dell PC workstation that controlled a Zeiss Axioplan 2 image microscope with an Optronics MicroFire CCD camera (1 600 × 1 200) with motorized X, Y and Z-focus for high-resolution image acquisition and digital quantitation.

### Statistical analysis

For each experiment, we used samples collected from either 1 or 2 embryos of the same genotype or postnatal mice from a given litter. We used 4-10 litters of mice for each prenatal experiment and 3-10 litters of mice for each postnatal experiment. Thus, we used data from 8 to 10 individuals (*n* = 8 or 10) per prenatal condition and data from 6 to 10 individuals (*n* = 6, 8 or 10) per postnatal condition. For behavioral experiments, 8-16 litters of mice were used. Statistical significance of differences between groups was analyzed by either two-tailed Student's *t*-test (Prism; GraphPad software) or ANOVA and *post hoc* tests and has been noted in individual figure legends. Significance was reported at *P* < 0.05.

## Author Contributions

AV conceived and designed the study; SL and PKT performed dissections, culture experiments, migration assays, histology, immunostainings, imaging, behavioral assays and analysis; SJ performed genotyping and cell culture; TK and RK contributed the hippocampal slice recording data and results; SS performed cryo sectioning of adult brains and immunohistochemistry; JSK conducted gene expression proﬁle analysis; JK and RKJ contributed the lectin perfusion and immunohistochemistry data and results; CD performed electrophysiological recordings in endothelial and neuronal cells; AE conducted ELISA; GS provided the GAD65-GFP line; SS, JK, RKJ and RK provided comments on the manuscript; AV supervised and coordinated all aspects of the project, analyzed data, prepared ﬁgures and wrote the manuscript.

## Competing Financial Interests

The authors declare no competing financial interests.

## Supplementary Material

Supplementary information, Figure S1 (A) Schematic representation of expression of several GABA_A_ receptor subunits in periventricular endothelial cells (PV ECS) that we have reported previously (Reference 16).

Supplementary information, Figure S2 (A-E) Labeling with isolectin B4 (A, B) and CD31/PECAM-1 (C, D) revealed marked reduction (yellow asterisks) in E15 *Gabrb3^ECKO^* telencephalon (B, D) when compared to *Gabrb3^fl/fl^* telencephalon (A, C).

Supplementary information, Figure S3 Experimental paradigm for social interaction test: (A-C) To test for social communication, a three-chambered social approach task (Sociability Cage w/2 Stranger Enclosure, Sterling Co.) was used.

Supplementary information, Figure S4 (A, B) High magnification images showing co-labeling with isolectin B4 and GABA antibodies.

Supplementary information, Figure S5 (A, B) Single images of VGAT immunoreactivity (green) in neurons/endothelial cells and merged images of VGAT (green) and isolectin B4 (red) in E13 *Vgat^fl/fl^* (A) and *Vgat^ECKO^* (B) neocortex.

Supplementary information, Figure S6 No marked change in neuroepithelial cell proliferation (S phase of the cell cycle) in *Vgat^ECKO^* telencephalon at E13 and E17.

Supplementary information, Figure S7 (A-E) Phosphorylated histone 3 (PHH3), a specific marker for cells undergoing mitosis was used to identify dividing progenitors in E15 *Vgat^fl/fl^* (A) and *Vgat^ECKO^* (B and C) ventral telencephalon.

Supplementary information, Figure S8 (A) Experiment schematic: E15 *Vgat^fl/fl^* periventricular endothelial cells (pv ecs) or *Vgat^ECKO^* pv ecs (that do not secrete GABA) were seeded in a specific track spanning a 35 mm culture dish (red dotted long box).

Supplementary information, Figure S9 (A-F) Telencephalic coronal sections of *Vgat^fl/fl^* (A, C, E) and *Vgat^ECKO^* (B, D, F) E17 embryos that received a single BrdU injection at E13, showing DAB immunohistochemistry with anti-BrdU antibody (n=10).

Supplementary information, Figure S10 (A, B) PHH3 profiles showed differences in E15 *Vgat^fl/fl^* (A) versus *Vgat^ECKO^* (B) dorsal telencephalon.

Supplementary information, Figure S11 Gene expression profiling of *Vgat^ECKO^* telencephalon predicts its postnatal phenotype.

Supplementary information, Figure S12 Epilepsy-related gene expression in *Vgat^ECKO^* telencephalon with links to childhood epilepsies.

Supplementary information, Figure S13 Graphical representation of genes that showed percentage change in *Vgat^ECKO^* telencephalon, compared to control, with respect to different categories of childhood epilepsies (isolated from McTague *et al*., 2015), continued from Figure S12.

Supplementary information, Figure S14 (A, B) A large population of cortical excitatory projection neurons express VGLUT1.

Supplementary information, Figure S15 Morphological analysis of *Vgat^fl/fl^* and *Vgat^ECKO^* somatosensory cortex: (A-B) Large basket cells in layer II/III from *Vgat^fl/fl^* somatosensory cortex showed more axonal arborizations when compared to *Vgat^ECKO^* cortex.

Supplementary information, Table S1 List of epilepsy related genes that were altered in *Vgat^ECKO^* telencephalon

Supplementary information, Movie S1 Movie depicts movements of a *Vgat^ECKO^* mouse and a littermate control.
