# Supplementary material for: Endothelial cell-derived GABA signaling modulates neuronal migration and postnatal behavior
Source: Cell Res. 2017 Oct 31;28(2):221–48. doi: 10.1038/cr.2017.135 (PMC5799810; doi:10.1038/cr.2017.135)
Supplement: Supplementary information, Figure S1 — (A) Schematic representation of expression of several GABAA receptor subunits in periventricular endothelial cells (PV ECS) that we have reported previously (Reference 16). [file cr2017135x1.pdf]

Figure S1

A **GABA$_A$ Receptor Subunits**

	α1	α2	α3	α4	α5	β1	β2	β3	γ1	γ2	γ3
PV ECS	+	+	-	+	+	+	+	+	-	+	-
								+			
								+			

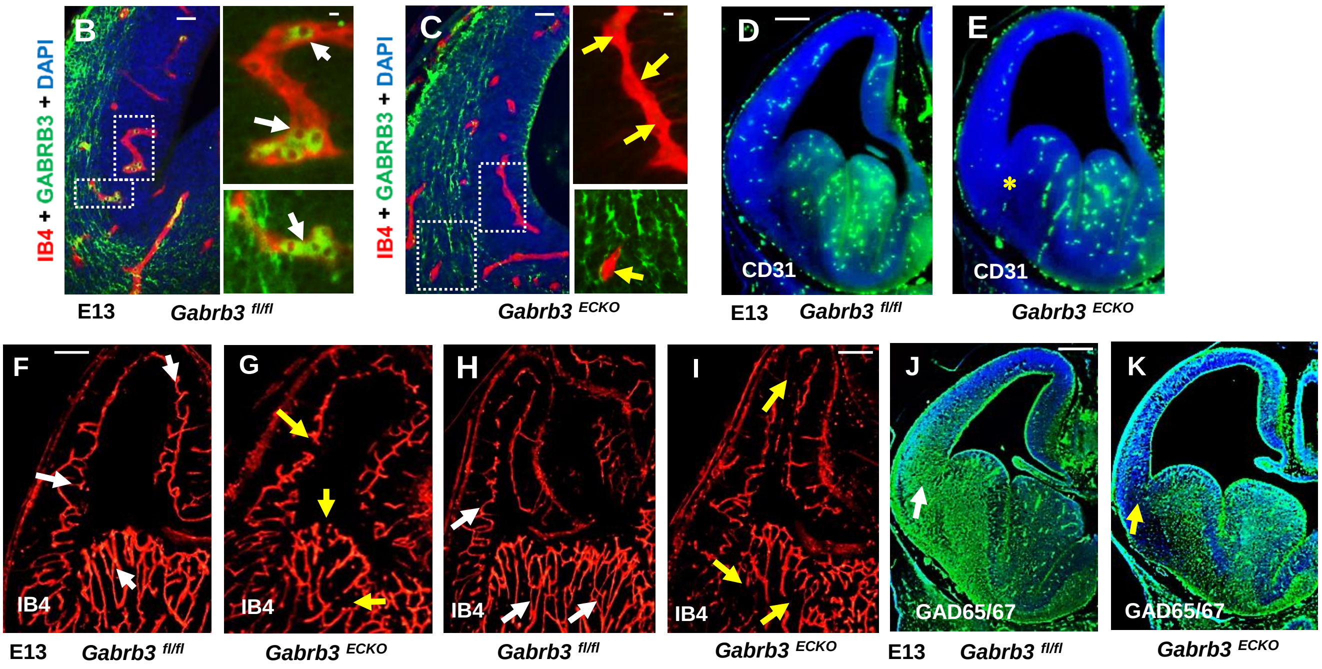

Figure S1: (A) Schematic representation of expression of several GABA$_A$ receptor subunits in periventricular endothelial cells (PV ECS) that we have reported previously (Reference 16). GABA$_A$ receptor beta 3 subunit showed enriched expression. (B, C) Low and high (insets) magnification images showing GABRB3 immunoreactivity in neurons and endothelial cells (B, white arrows) of E13 *Gabrb3 $^{fl/fl}$* telencephalon and its specific loss in endothelial cells of *Gabrb3 ^{ECKO}* telencephalon (C, yellow arrows). (D, E) Labeling with CD31/PECAM-1 revealed marked reduction (yellow asterisk) in E13 *Gabrb3 ^{ECKO}* telencephalon (E) when compared to *Gabrb3 $^{fl/fl}$* telencephalon (D). (F-I) In E13 *Gabrb3 $^{fl/fl}$* telencephalon, while the tube-like plexus of periventricular vessels in the ganglionic eminence and dorsal telencephalon, labeled with isolectin B4 (IB4), was continuous and well formed (white arrows, F, H), it was discontinuous and irregular (yellow arrows, G, I) in *Gabrb3 ^{ECKO}* telencephalon at both rostral (F, G) and caudal levels (H, I). (J, K) GAD65/67 immunoreactivity showed decreased stream of GABA neurons in E13 *Gabrb3 ^{ECKO}* telencephalon (yellow arrow, K) when compared to *Gabrb3 $^{fl/fl}$* telencephalon (white arrow, J). Collective data from 10 µm thick coronal paraffin sections (n=8). Scale bar: B, C, 50 µm; insets, 25 µm; D, 100 µm (applies E-K).
