# Supplementary material for: Endothelial cell-derived GABA signaling modulates neuronal migration and postnatal behavior
Source: Cell Res. 2017 Oct 31;28(2):221–48. doi: 10.1038/cr.2017.135 (PMC5799810; doi:10.1038/cr.2017.135)
Supplement: Supplementary information, Figure S2 — (A-E) Labeling with isolectin B4 (A, B) and CD31/PECAM-1 (C, D) revealed marked reduction (yellow asterisks) in E15 Gabrb3ECKO telencephalon (B, D) when compared to Gabrb3fl/fl telencephalon (A, C). [file cr2017135x2.pdf]

Figure S2

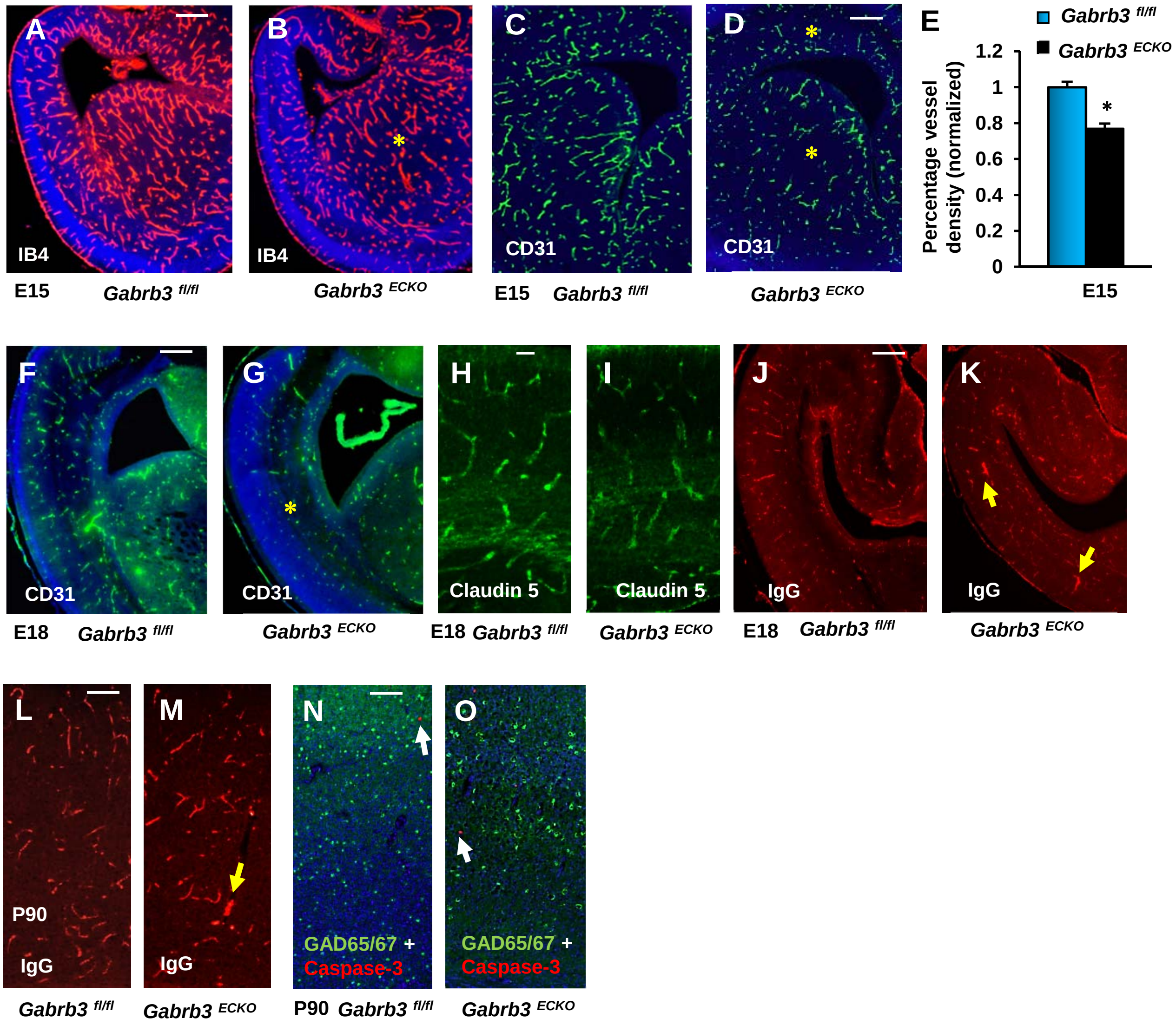

Figure S2: (A-E) Labeling with isolectin B4 (A, B) and CD31/PECAM-1 (C, D) revealed marked reduction (yellow asterisks) in E15 *Gabrb3* ^{ECKO} telencephalon (B, D) when compared to *Gabrb3* $^{fl/fl}$ telencephalon (A, C). (E) A significant reduction of vessel densities was observed in E15 *Gabrb3* ^{ECKO} telencephalon; Data represents mean ± SD (n=11, *P<0.0001, Student's t-test). (F, G) CD31/PECAM-1 labeling was reduced in E18 *Gabrb3* ^{ECKO} telencephalon (yellow asterisk, G) when compared to *Gabrb3* $^{fl/fl}$ telencephalon (F). (H, I) Claudin 5 immunoreactivity showed subtle changes in E18 *Gabrb3* ^{ECKO} dorsal telencephalon (I) when compared to *Gabrb3* $^{fl/fl}$ (H) telencephalon. (J-M) Images of IgG staining from E18 *Gabrb3* $^{fl/fl}$ and *Gabrb3* ^{ECKO} dorsal telencephalon (J, K) and P90 somatosensory cortex (L, M). IgG was localized to vessels and no IgG leakage was observed from *Gabrb3* ^{ECKO} vessels during embryonic and adult stages. However, enlarged and dilated vessels were observed in *Gabrb3* ^{ECKO} brains (yellow arrows, K, M). (N, O) Anti-active caspase 3 immunohistochemistry combined with anti-GAD65/67 staining in P90 mice. Active caspase profiles were rarely observed in *Gabrb3* $^{fl/fl}$ and *Gabrb3* ^{ECKO} cortex. These data suggest that the decreased number of interneurons observed in the *Gabrb3* ^{ECKO} cortex is not due to apoptosis following impaired blood flow. Collective data from 10 µm thick coronal paraffin sections of the embryonic brain (A-K) and 40 µm thick frozen sections of the adult brain (L-O); (n=8). Scale bar: A, 100 µm (applies to B-D, F, G, J-O); H, 50 µm (applies to I).
