# Supplementary material for: Endothelial cell-derived GABA signaling modulates neuronal migration and postnatal behavior
Source: Cell Res. 2017 Oct 31;28(2):221–48. doi: 10.1038/cr.2017.135 (PMC5799810; doi:10.1038/cr.2017.135)
Supplement: Supplementary information, Figure S3 — Experimental paradigm for social interaction test: (A-C) To test for social communication, a three-chambered social approach task (Sociability Cage w/2 Stranger Enclosure, Sterling Co.) was used. [file cr2017135x3.pdf]

Figure S3

A

Sociability Phase:
Mouse 1 versus
Object

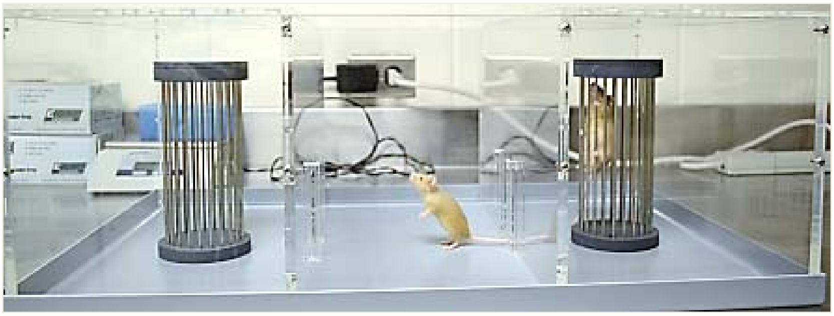

B

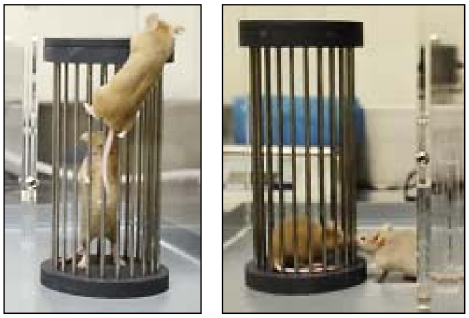

C

Social Novelty:
Familiar Mouse 1
versus
Novel Mouse 2

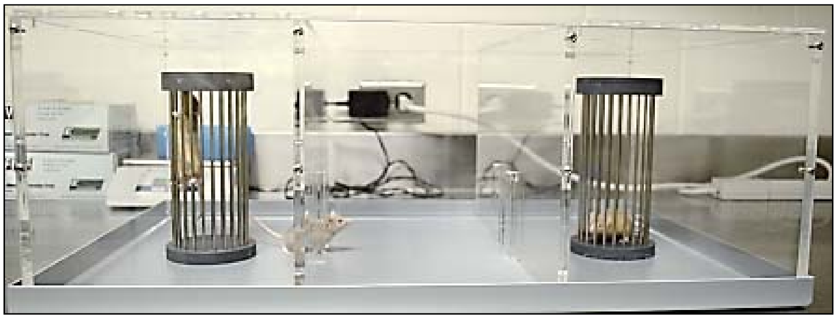

Figure S3: Experimental paradigm for social interaction test: (A-C) To test for social communication, a three-chambered social approach task (Sociability Cage w/2 Stranger Enclosure, Sterling Co.) was used. The first session of the test is based on the concept of free choice that is given to the test mouse (in the center chamber, A) to prefer to spend time with a stranger mouse 1 enclosed in a circular wire cup that allows sensory interactions (smell, sight, sound, taste; A, B) or to spend time alone with an identical but empty cup in another chamber (A). Normal mice will spend more time in the chamber with novel mouse 1 compared to the compartment with the empty cup which is indicative of normal sociability, motivation and affiliation. The second session of the test estimates social novelty and social memory. Preference for social novelty is based on the inclination of the test mouse to spend time with the now familiar mouse 1 versus a new stranger mouse 2 (C). Usually wild type animals will recall their previous contact with mouse 1 and will prefer to explore the novel mouse 2 which would indicate intact social memory and preference to gain new experiences.
