# Supplementary material for: Endothelial cell-derived GABA signaling modulates neuronal migration and postnatal behavior
Source: Cell Res. 2017 Oct 31;28(2):221–48. doi: 10.1038/cr.2017.135 (PMC5799810; doi:10.1038/cr.2017.135)
Supplement: Supplementary information, Figure S4 — (A, B) High magnification images showing co-labeling with isolectin B4 and GABA antibodies. [file cr2017135x4.pdf]

Figure S4

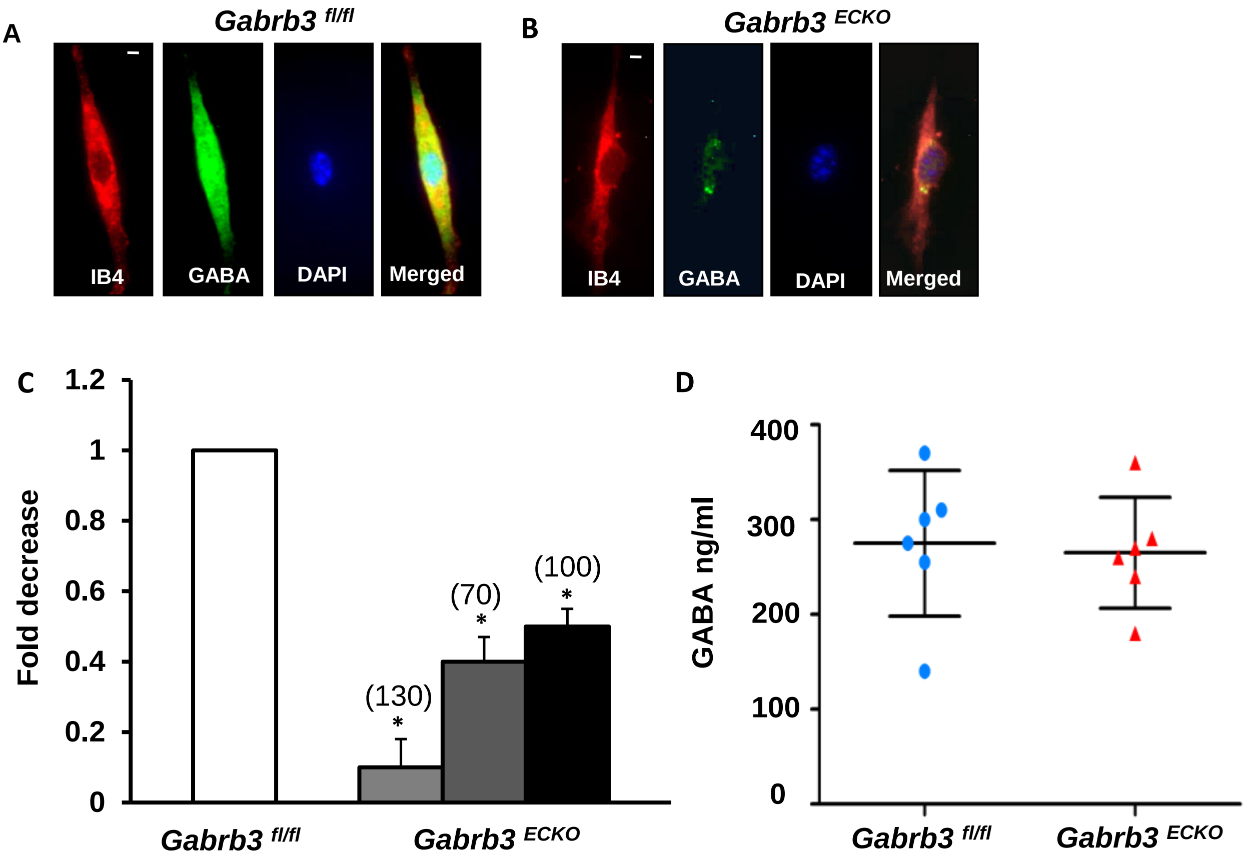

Figure S4: (A, B) High magnification images showing co-labeling with isolectin B4 and GABA antibodies. GABA expression was significantly down regulated in *Gabrb3* ^{ECKO} periventricular endothelial cells (B) when compared to *Gabrb3* $^{fl/fl}$ endothelial cells (A). (C) GABA expression in all *Gabrb3* ^{ECKO} periventricular endothelial cells was overall significantly decreased when compared to control endothelial cells. Fold decrease in GABA expression in individual *Gabrb3* ^{ECKO} periventricular endothelial cells (numbers noted in brackets) was normalised with respect to *Gabrb3* $^{fl/fl}$ endothelial cells. Some variability in GABA expression fold decrease was observed between *Gabrb3* ^{ECKO} endothelial cells. Data represents mean ± SD (n = 300 cells were analyzed and quantified). (D) Pial endothelial cells do not express GABRB3 and are not affected by the deletion. GABA secretion from *Gabrb3* ^{ECKO} pial endothelial cells was comparable to floxed littermate controls; Data represents mean ± SD (n=6). Scale bars: A, 15 μm; (applies to B).
