# Supplementary material for: Endothelial cell-derived GABA signaling modulates neuronal migration and postnatal behavior
Source: Cell Res. 2017 Oct 31;28(2):221–48. doi: 10.1038/cr.2017.135 (PMC5799810; doi:10.1038/cr.2017.135)
Supplement: Supplementary information, Figure S5 — (A, B) Single images of VGAT immunoreactivity (green) in neurons/endothelial cells and merged images of VGAT (green) and isolectin B4 (red) in E13 Vgatfl/fl (A) and VgatECKO (B) neocortex. [file cr2017135x5.pdf]

Figure S5

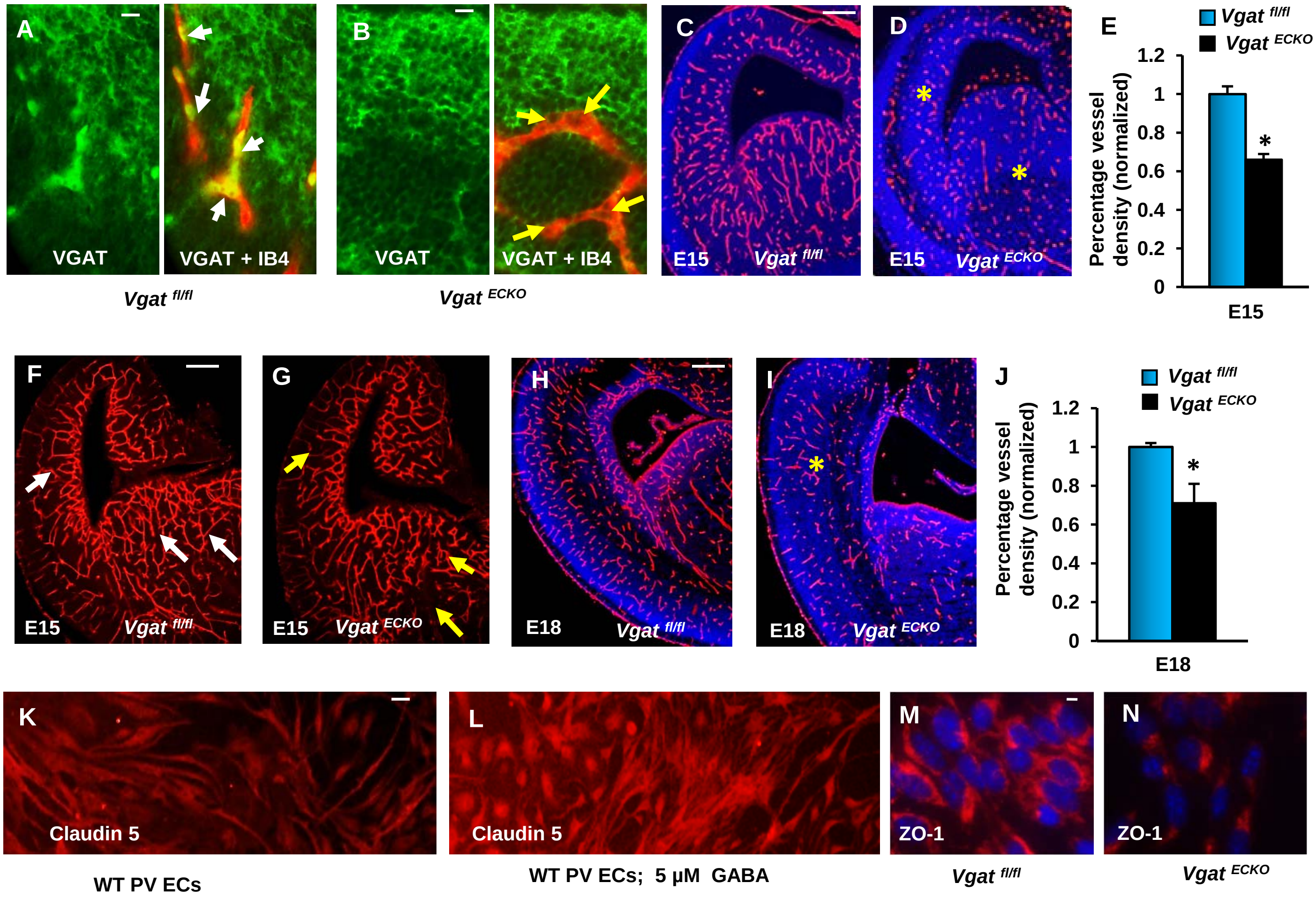

Figure S5: (A, B) Single images of VGAT immunoreactivity (green) in neurons/endothelial cells and merged images of VGAT (green) and isolectin B4 (red) in E13 *Vgat* $^{fl/fl}$ (A) and *Vgat* ^{ECKO} (B) neocortex. Specific loss of VGAT immunoreactivity was observed in *Vgat* ^{ECKO} endothelial cells (B, yellow arrows) versus *Vgat* $^{fl/fl}$ endothelial cells (A, white arrows). (C-E) Labeling with isolectin B4 revealed marked reduction (yellow asterisks, D) in E15 *Vgat* ^{ECKO} telencephalon when compared to *Vgat* $^{fl/fl}$ telencephalon. A significant reduction in vessel densities was observed in E15 *Vgat* ^{ECKO} telencephalon (E); Data represents mean ± SD (n=11, *P<0.05, Student's t-test). (F, G) The organized rhombic vascular patterns of periventricular vessel network seen in *Vgat* $^{fl/fl}$ telencephalon (white arrows, F) was disrupted in the *Vgat* ^{ECKO} telencephalon (yellow arrows, G). (H, I) Isolectin B4^{+} vessels were significantly reduced in E18 *Vgat* ^{ECKO} pallium compared with *Vgat* $^{fl/fl}$ pallium (yellow asterisks, I). (J) Quantification of cortical vessel densities (n=8, mean ± SD, *P<0.05, Student's t-test). (K, L) Claudin 5 expression was increased in wild type (WT) periventricular endothelial cells (PV ECs) after treatment with 5 µM GABA for 24 hours, suggesting that GABA can induce tight junction protein expression and regulate BBB development. (M, N) ZO-1 expression was reduced in *Vgat* ^{ECKO} endothelial cells when compared to *Vgat* $^{fl/fl}$ endothelial cells. Scale bars: A, 30 µm (applies to B); C, 100 µm (applies to D, F-I); K, 50 µm (applies to L); M, 20 µm, (applies to N).
