# Supplementary material for: Endothelial cell-derived GABA signaling modulates neuronal migration and postnatal behavior
Source: Cell Res. 2017 Oct 31;28(2):221–48. doi: 10.1038/cr.2017.135 (PMC5799810; doi:10.1038/cr.2017.135)
Supplement: Supplementary information, Figure S6 — No marked change in neuroepithelial cell proliferation (S phase of the cell cycle) in VgatECKO telencephalon at E13 and E17. [file cr2017135x6.pdf]

Figure S6

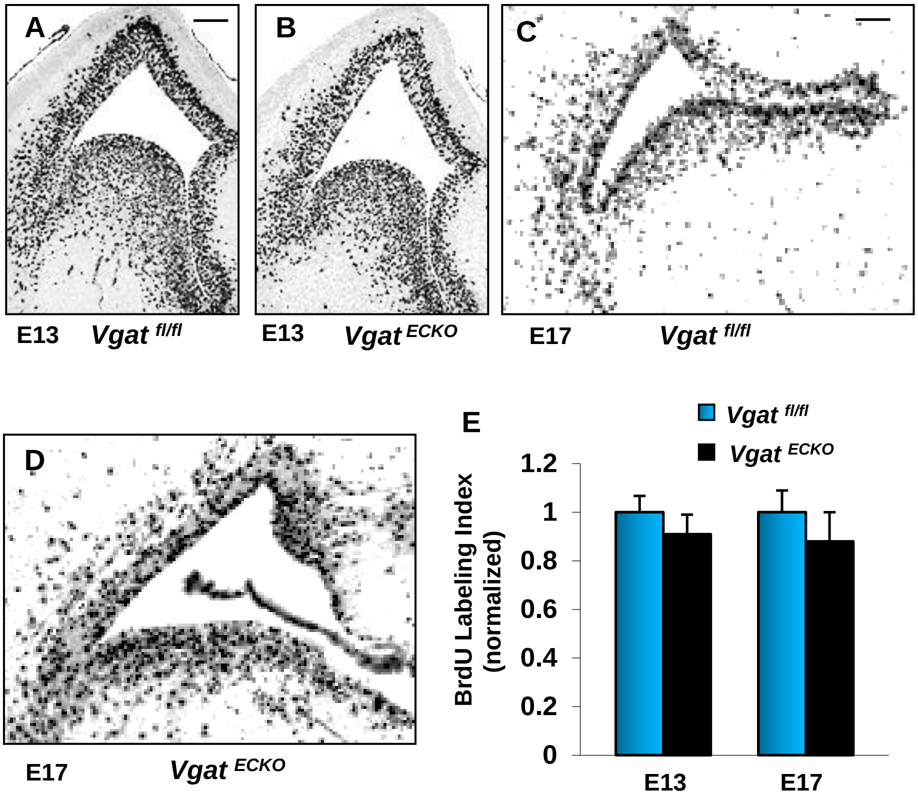

Figure S6: No marked change in neuroepithelial cell proliferation (S phase of the cell cycle) in *Vgat* ^{ECKO} telencephalon at E13 and E17. Pregnant dams were injected with a single dose of bromodeoxyuridine (BrdU) (50 µg per g body weight, i.p.; Sigma) 2 h before sacrifice. Embryos were removed and decapitated, and embryonic heads were immersed in zinc fixative (BD Pharmingen) for 24 hours and processed for paraffin wax histology. (A-D) DAB immunohistochemistry with anti-BrdU antibody was performed on coronal, 8-µm sections. Sections of E13 (A, B) and E17 (C, D) *Vgat* $^{fl/fl}$ and *Vgat* ^{ECKO} telencephalon showing BrdU-labeled (black) cells following BrdU exposure. (E) Quantification of BrdU labeling index showed no significant difference when compared to *Vgat* $^{fl/fl}$ telencephalon (n=10). Scale bars: A, 100 um (applies to B-D).
