# Supplementary material for: Endothelial cell-derived GABA signaling modulates neuronal migration and postnatal behavior
Source: Cell Res. 2017 Oct 31;28(2):221–48. doi: 10.1038/cr.2017.135 (PMC5799810; doi:10.1038/cr.2017.135)
Supplement: Supplementary information, Figure S7 — (A-E) Phosphorylated histone 3 (PHH3), a specific marker for cells undergoing mitosis was used to identify dividing progenitors in E15 Vgatfl/fl (A) and VgatECKO (B and C) ventral telencephalon. [file cr2017135x7.pdf]

Figure S7

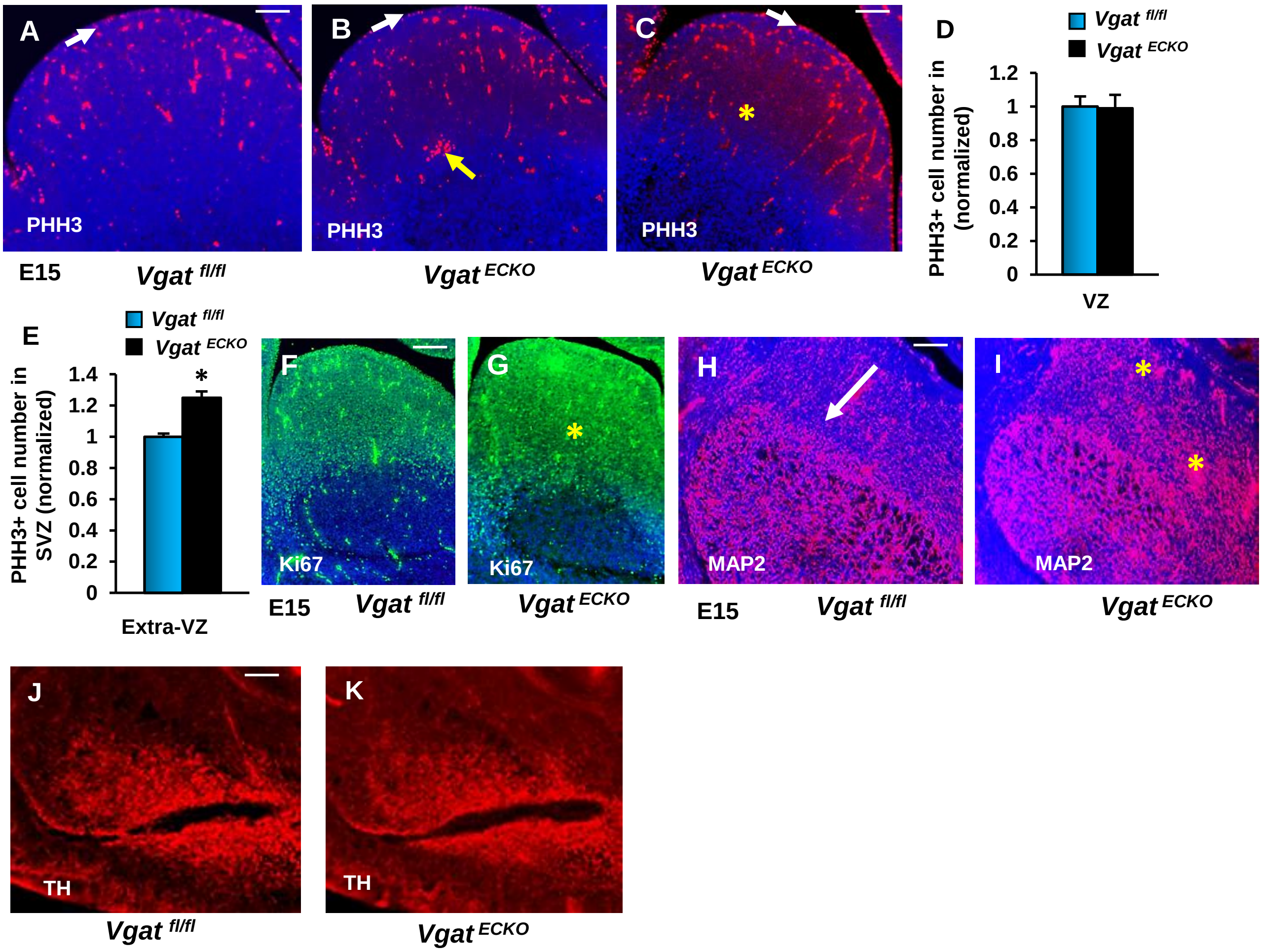

Figure S7: (A-E) Phosphorylated histone 3 (PHH3), a specific marker for cells undergoing mitosis was used to identify dividing progenitors in E15 *Vgat*$^{fl/fl}$ (A) and *Vgat*^{ECKO} (B and C) ventral telencephalon. Sections were also stained with DAPI. There were no significant differences in the number of PHH3$^+$ cells at the VZ surface (white arrows, A-C) of *Vgat*$^{fl/fl}$ and *Vgat*^{ECKO} telencephalon (D). However, abnormal PHH3$^+$ profiles were observed in the extra-VZ surface of *Vgat*^{ECKO} telencephalon with clusters of cells (yellow arrows, B) or lack of cells (yellow asterisk, C) in some areas. A small, but significant increase in the number of PHH3$^+$ cells was observed in the extra-VZ surface when quantified (E). Data represents mean ± SD (n=7, *P<0.05, Student's t-test. (F, G) An increase in cell proliferation was observed at the extra-VZ surface of *Vgat*^{ECKO} ventral telencephalon (G) by labeling with Ki67 (which labels all phases of the cell cycle - S, G2, M and G1 phases) when compared to *Vgat*$^{fl/fl}$ ventral telencephalon (F). (H, I) Neuronal differentiation marker MAP2 (red) and nuclear marker DAPI (blue) labeling in *Vgat*$^{fl/fl}$ and *Vgat*^{ECKO} ventral telencephalon at E15. MAP2 immunoreactivity was localized in *Vgat*$^{fl/fl}$ striatum (H, white arrow). Some MAP2 labeling was also observed outside the differentiation zone in *Vgat*^{ECKO} ventral telencephalon (I, yellow asterisks). (J, K) Tyrosine hydroxylase (TH) staining showed no significant differences in *Vgat*$^{fl/fl}$ and *Vgat*^{ECKO} ventral telencephalon. Collective data from 10 µm thick coronal paraffin sections (n=7). Scale bars: A, 100 um (applies to B, C, F-K).
