# Supplementary material for: Endothelial cell-derived GABA signaling modulates neuronal migration and postnatal behavior
Source: Cell Res. 2017 Oct 31;28(2):221–48. doi: 10.1038/cr.2017.135 (PMC5799810; doi:10.1038/cr.2017.135)
Supplement: Supplementary information, Figure S8 — (A) Experiment schematic: E15 Vgatfl/fl periventricular endothelial cells (pv ecs) or VgatECKO pv ecs (that do not secrete GABA) were seeded in a specific track spanning a 35 mm culture dish (red dotted long box). [file cr2017135x8.pdf]

Figure S8

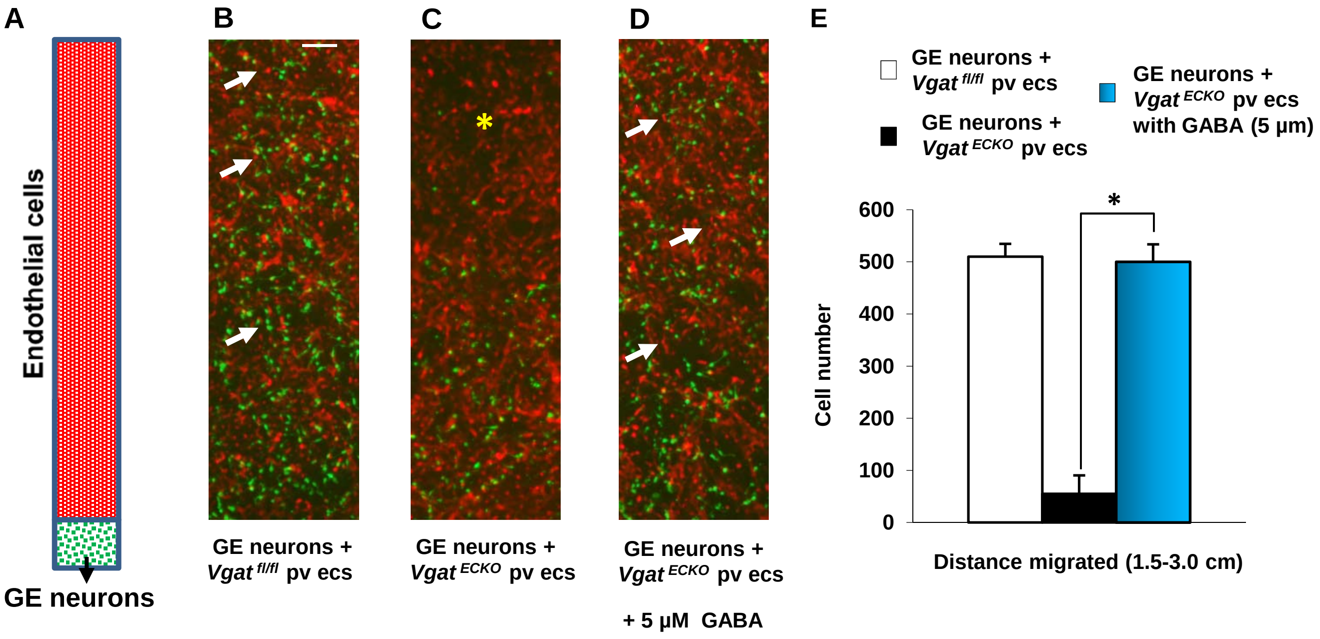

Figure S8: (A) Experiment schematic: E15 *Vgat* $^{fl/fl}$ periventricular endothelial cells (pv ecs) or *Vgat* ^{ECKO} pv ecs (that do not secrete GABA) were seeded in a specific track spanning a 35 mm culture dish (red dotted long box). GE neurons from E15 GAD65-GFP telencephalon were plated at one end of the track (green dotted small box). One group of *Vgat* ^{ECKO} pv ecs with GE neurons was treated with 5 µM GABA**.** (B-D) While GE neurons failed to migrate long-distance on *Vgat* ^{ECKO} pv ecs (C), robust neuronal migration was observed in the control group (B) and in the *Vgat* ^{ECKO} pv ecs treated with 5 µM GABA (D). White arrows point to robust neuronal migration and yellow asterisk reveals lack of migration. (E) Quantification of cell migration in B-D; Data represents mean ± SD (n=10, *P<0.05, Student's t-test). Scale bars: B, 100 um (applies to C, D).
