# Supplementary material for: Endothelial cell-derived GABA signaling modulates neuronal migration and postnatal behavior
Source: Cell Res. 2017 Oct 31;28(2):221–48. doi: 10.1038/cr.2017.135 (PMC5799810; doi:10.1038/cr.2017.135)
Supplement: Supplementary information, Figure S9 — (A-F) Telencephalic coronal sections of Vgatfl/fl (A, C, E) and VgatECKO (B, D, F) E17 embryos that received a single BrdU injection at E13, showing DAB immunohistochemistry with anti-BrdU antibody (n=10). [file cr2017135x9.pdf]

Figure S9

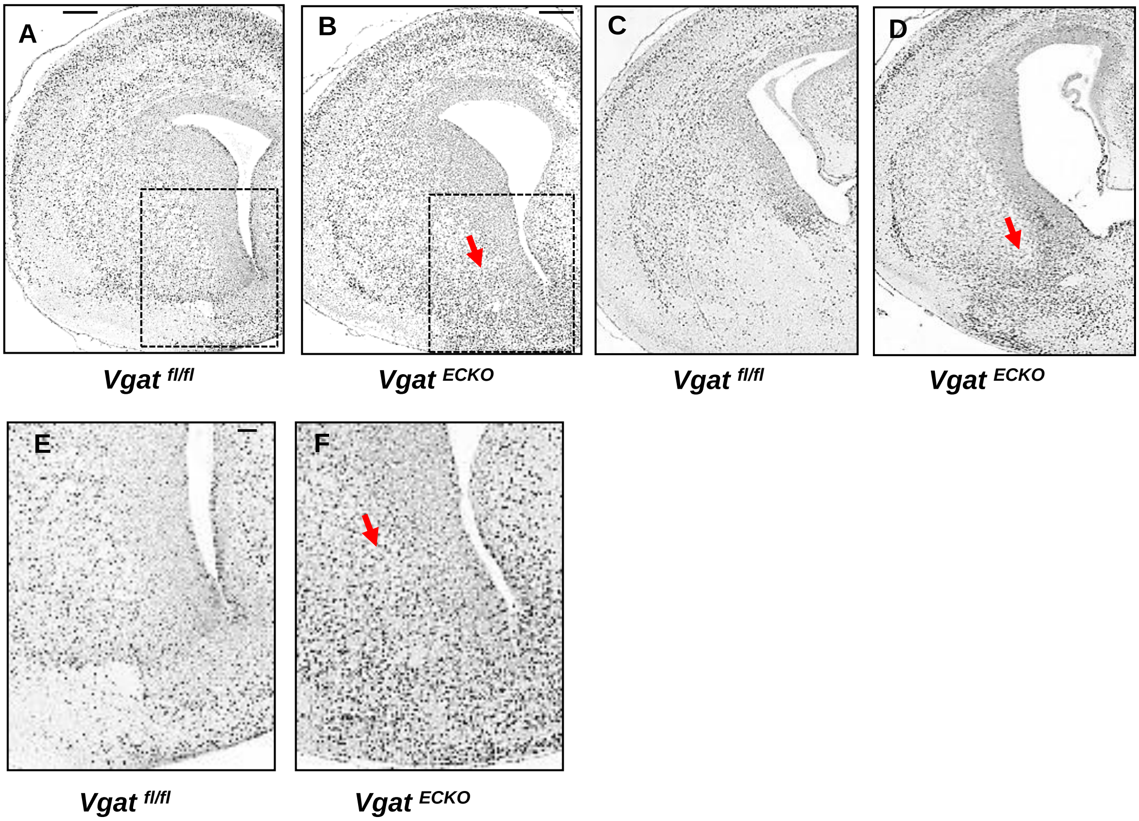

Figure S9: (A-F) Telencephalic coronal sections of *Vgat* $^{fl/fl}$ (A, C, E) and *Vgat* ^{ECKO} (B, D, F) E17 embryos that received a single BrdU injection at E13, showing DAB immunohistochemistry with anti-BrdU antibody (n=10). Several stalled BrdU+ cells were observed in *Vgat* ^{ECKO} ventral telencephalon at both rostral (B) and caudal (D) levels (red arrows). Insets in A and B are magnified in E and F. Scale bars: A, 100 um (applies to B-D), E, 50 um (applies to F).
