# Supplementary material for: Endothelial cell-derived GABA signaling modulates neuronal migration and postnatal behavior
Source: Cell Res. 2017 Oct 31;28(2):221–48. doi: 10.1038/cr.2017.135 (PMC5799810; doi:10.1038/cr.2017.135)
Supplement: Supplementary information, Figure S10 — (A, B) PHH3 profiles showed differences in E15 Vgatfl/fl (A) versus VgatECKO (B) dorsal telencephalon. [file cr2017135x10.pdf]

Figure S10

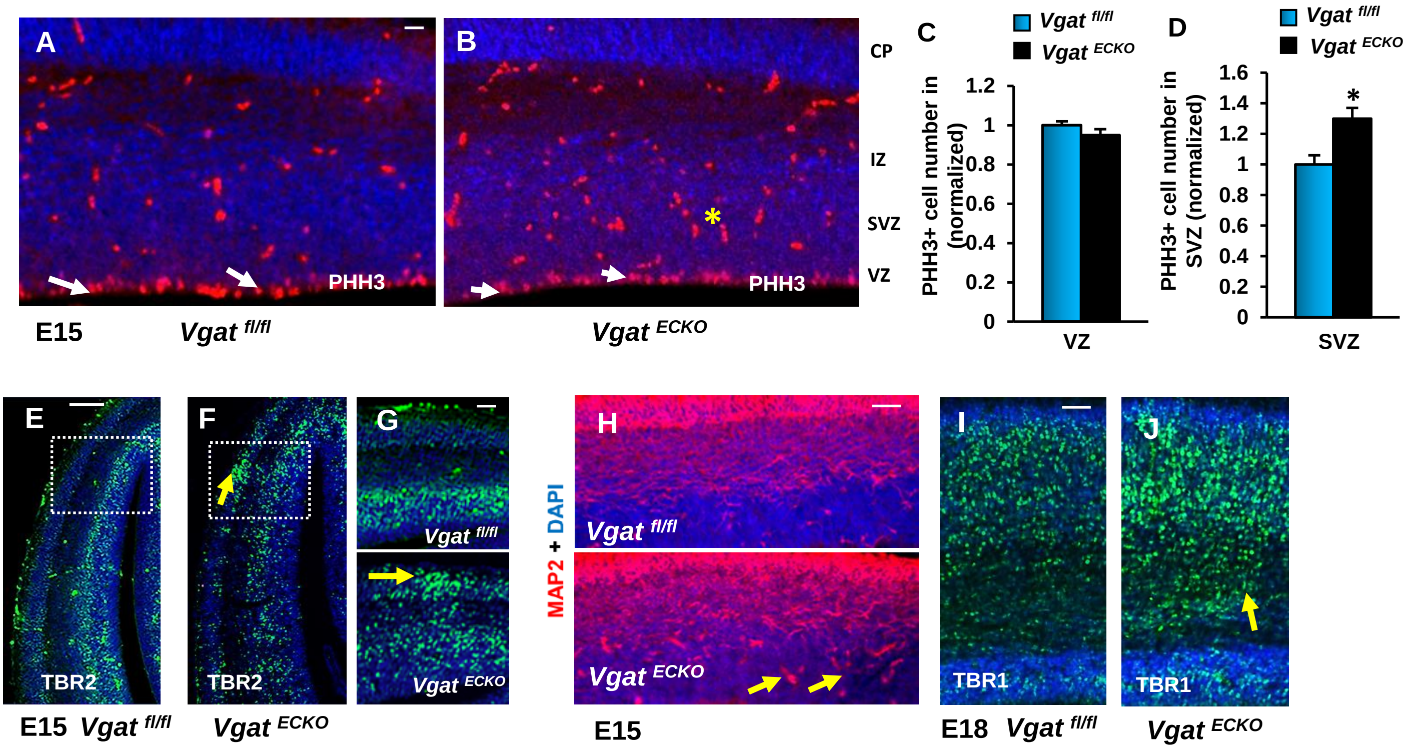

Figure S10: (A, B) PHH3 profiles showed differences in E15 *Vgat* $^{fl/fl}$ (A) versus *Vgat* ^{ECKO} (B) dorsal telencephalon. While no significant differences in the number of PHH3^{+} cells was observed at the VZ surface (white arrows, A, B) of both groups, PHH3^{+} cells were increased in the SVZ of *Vgat* ^{ECKO} dorsal telencephalon (yellow asterisk, B) when compared to *Vgat* $^{fl/fl}$ telencephalon. (C, D) Quantification of the number of PHH3^{+} cells in the VZ and SVZ. Data represents mean ± SD (n=7, *P<0.05, Student's t-test). (E-G) The distribution of cells expressing Tbr2, a transcription factor that selectively marks intermediate progenitor cells (IPCs, precursors of cortical projection neurons) was examined in the dorsal telencephalon. While Tbr2 expression was localized to VZ and SVZ of E15 *Vgat* $^{fl/fl}$ telencephalon (E), ectopic Tbr2^{+} cells was observed in the IZ and cortical plate of *Vgat* ^{ECKO} telencephalon (F, yellow arrow). Boxed regions in E and F have been magnified in G. (H) MAP2 immunoreactivity revealed normal neuronal differentiation zone of E15 *Vgat* $^{fl/fl}$ pallium, while MAP2^{+} cells were abnormally positioned in the *Vgat* ^{ECKO} IZ-SVZ (yellow arrows). (I, J) Expression of the transcription factor Tbr1, that marks newly generated projection neurons of the cerebral cortex upon exit from the cell cycle was also examined. Tbr1 immunoreactivity revealed a continuous uniform band of Tbr1-positive cells in the cortical plate of E18 *Vgat* $^{fl/fl}$ embryos (I). On the other hand, abnormally arranged Tbr1-positive cells were detected outside the cortical plate, trailing in the IZ of *Vgat* ^{ECKO} embryos (J, yellow arrow). Collective data from 10 µm thick coronal paraffin sections (n=7). All sections were stained with DAPI. Scale bars: A, 50 um (applies to B, G); E, 100 um (applies to F), H , 75 um (applies to I, J).
