# Supplementary material for: Endothelial cell-derived GABA signaling modulates neuronal migration and postnatal behavior
Source: Cell Res. 2017 Oct 31;28(2):221–48. doi: 10.1038/cr.2017.135 (PMC5799810; doi:10.1038/cr.2017.135)
Supplement: Supplementary information, Figure S11 — Gene expression profiling of VgatECKO telencephalon predicts its postnatal phenotype. [file cr2017135x11.pdf]

Figure S11

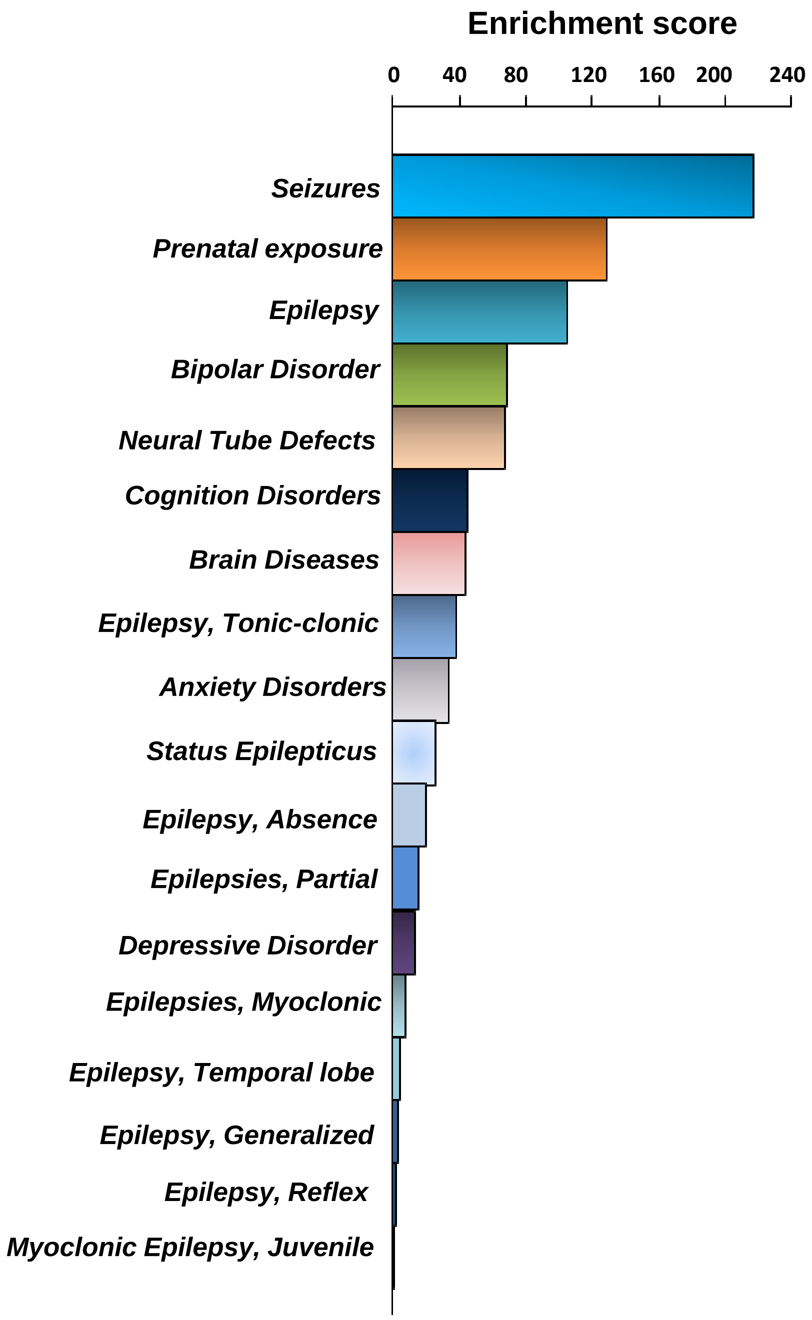

Figure S11: Gene expression profiling of *Vgat* ^{ECKO} telencephalon predicts its postnatal phenotype. A classification of genes expressed in E18 *Vgat* ^{ECKO} telencephalon by MTRR CTD analysis shows particular enrichment in several neurological and psychiatric disease categories. Seizures/Epilepsy tops the list.
