# Supplementary material for: Endothelial cell-derived GABA signaling modulates neuronal migration and postnatal behavior
Source: Cell Res. 2017 Oct 31;28(2):221–48. doi: 10.1038/cr.2017.135 (PMC5799810; doi:10.1038/cr.2017.135)
Supplement: Supplementary information, Figure S12 — Epilepsy-related gene expression in VgatECKO telencephalon with links to childhood epilepsies. [file cr2017135x12.pdf]

Figure S12

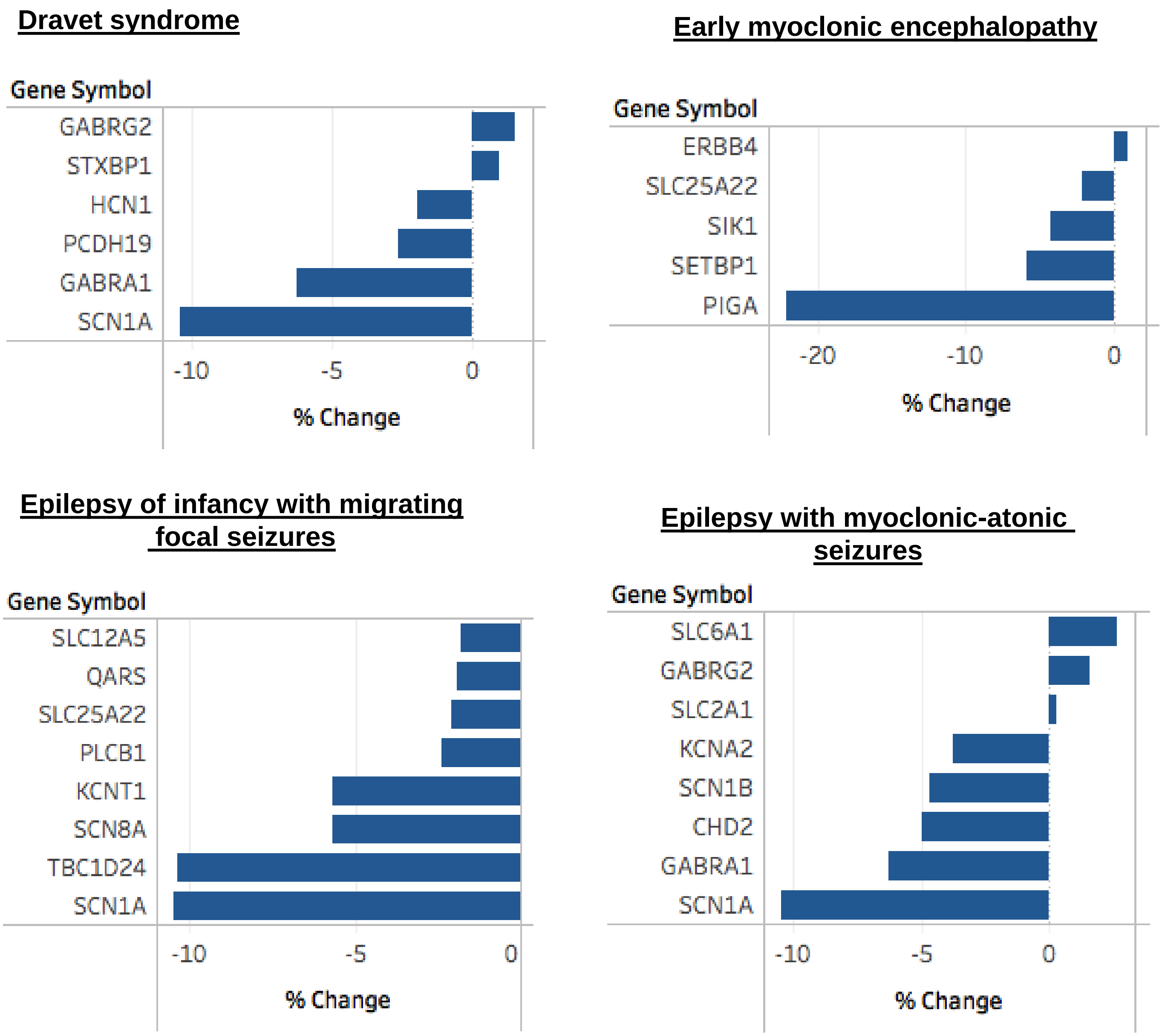

Figure S12: Epilepsy-related gene expression in *Vgat* ^{ECKO} telencephalon with links to childhood epilepsies. Graphical representation of genes that showed percentage change in *Vgat* ^{ECKO} telencephalon, compared to controls, with respect to different categories of childhood epilepsies (isolated from McTague *etal*., 2015), continued in Figure S13.
