# Supplementary material for: Endothelial cell-derived GABA signaling modulates neuronal migration and postnatal behavior
Source: Cell Res. 2017 Oct 31;28(2):221–48. doi: 10.1038/cr.2017.135 (PMC5799810; doi:10.1038/cr.2017.135)
Supplement: Supplementary information, Figure S13 — Graphical representation of genes that showed percentage change in VgatECKO telencephalon, compared to control, with respect to different categories of childhood epilepsies (isolated from McTague et al., 2015), continued from Figure S12. [file cr2017135x13.pdf]

Figure S13

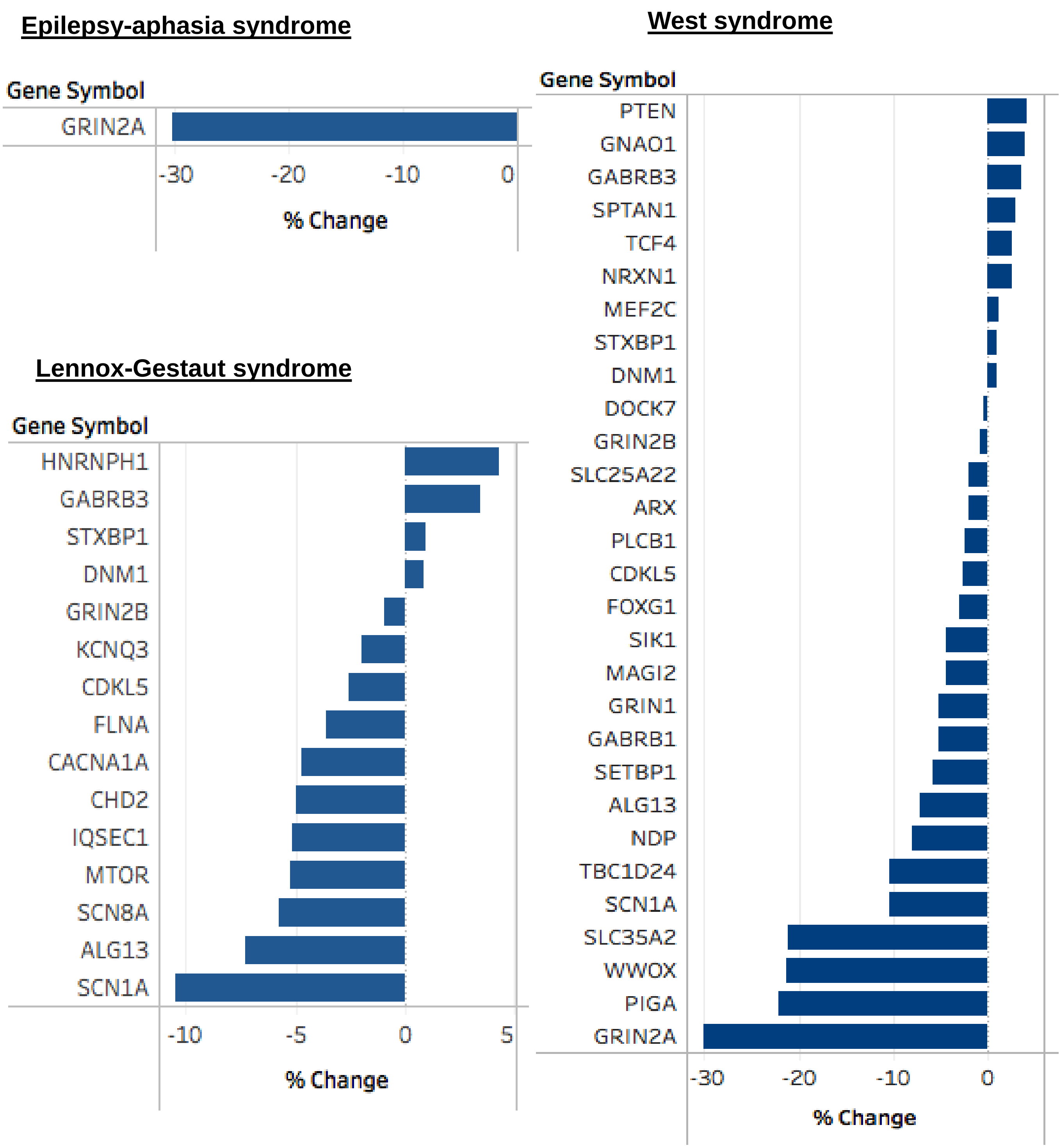

Figure S13: Graphical representation of genes that showed percentage change in *Vgat* [ECKO] telencephalon, compared to control, with respect to different categories of childhood epilepsies (isolated from McTague *etal*., 2015), continued from Figure S12.
