# Supplementary material for: Endothelial cell-derived GABA signaling modulates neuronal migration and postnatal behavior
Source: Cell Res. 2017 Oct 31;28(2):221–48. doi: 10.1038/cr.2017.135 (PMC5799810; doi:10.1038/cr.2017.135)
Supplement: Supplementary information, Figure S14 — (A, B) A large population of cortical excitatory projection neurons express VGLUT1. [file cr2017135x14.pdf]

Figure S14

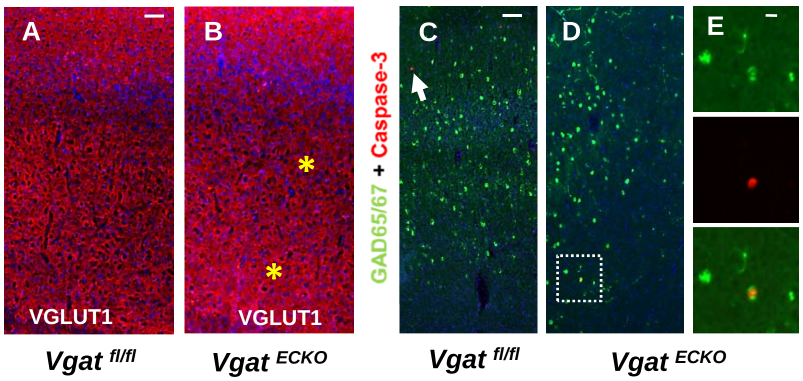

Figure S14: (A, B) A large population of cortical excitatory projection neurons express VGLUT1. Therefore, anti-VGLUT1/DAPI immunohistochemisty was used to analyze glutamatergic projection neuron distribution in P30 *Vgat* $^{fl/fl}$ and *Vgat* ^{ECKO} parietal cortex. Abnormal distribution of $VGLUT^{+}$ neurons was observed in *Vgat* ^{ECKO} cortex (yellow asterisks, B) when compared to the uniform distribution in *Vgat* $^{fl/fl}$ cortex (A). (C-E) To assess neuronal death in *Vgat* ^{ECKO} mice, anti-active caspase-3 and GAD65/67 immunohistochemistry was performed. Active caspase profiles were few and insignificant in P30 *Vgat* $^{fl/fl}$ and *Vgat* ^{ECKO} cortex. White arrow points to $caspase^{+}$ cell and inset in D has been magnified in E to show individual GAD65/67, caspase-3 and merged images. Scale bars: A, 100 um (applies to B-D); E, 50 um.
