# Supplementary material for: Endothelial cell-derived GABA signaling modulates neuronal migration and postnatal behavior
Source: Cell Res. 2017 Oct 31;28(2):221–48. doi: 10.1038/cr.2017.135 (PMC5799810; doi:10.1038/cr.2017.135)
Supplement: Supplementary information, Figure S15 — Morphological analysis of Vgatfl/fl and VgatECKO somatosensory cortex: (A-B) Large basket cells in layer II/III from Vgatfl/fl somatosensory cortex showed more axonal arborizations when compared to VgatECKO cortex. [file cr2017135x15.pdf]

Figure S15

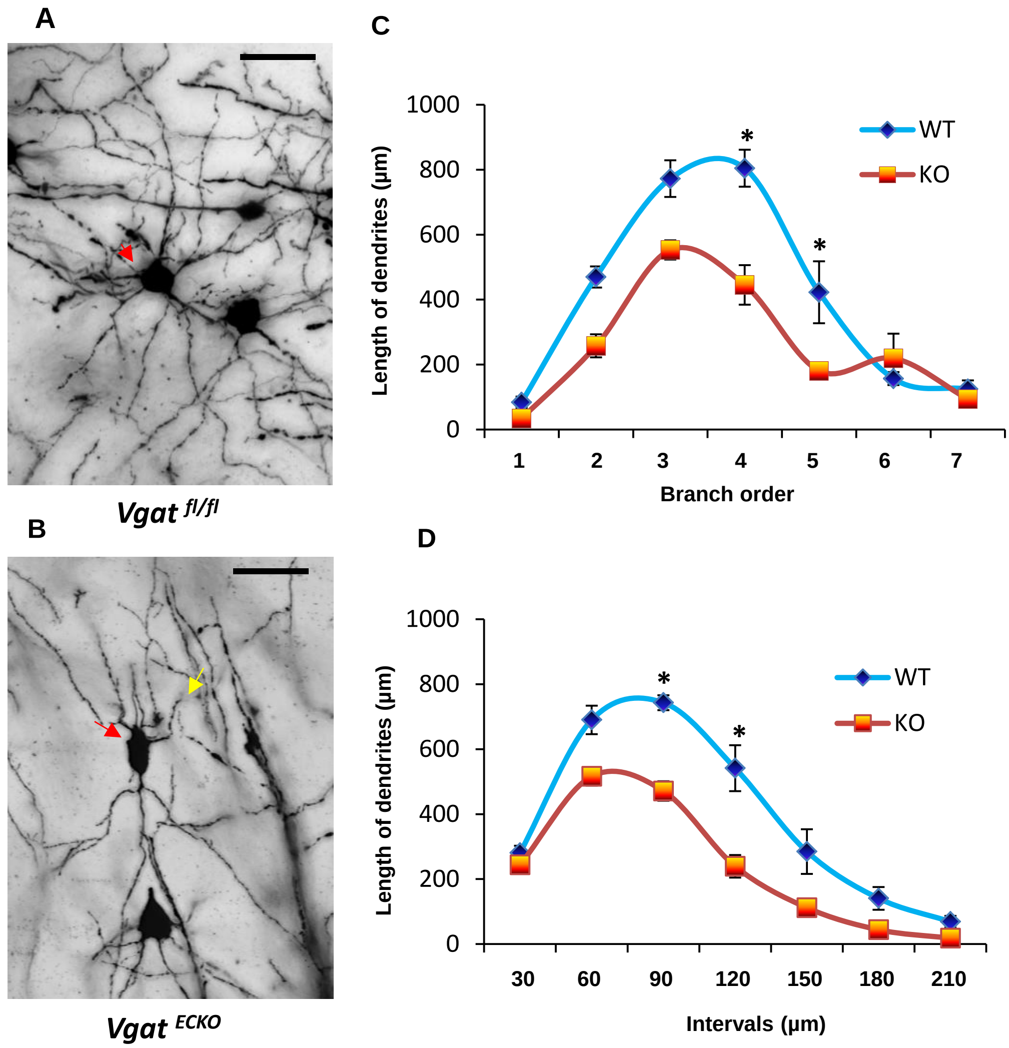

Figure S15: Morphological analysis of *Vgat* $^{fl/fl}$ and *Vgat* ^{ECKO} somatosensory cortex: (A-B) Large basket cells in layer II/III from *Vgat* $^{fl/fl}$ somatosensory cortex showed more axonal arborizations when compared to *Vgat* ^{ECKO} cortex. (C) Comparison of dendritic length x branch order of basket cells between *Vgat* $^{fl/fl}$ and *Vgat* ^{ECKO} somatosensory cortex. The results showed a significant reduction in dendritic lengths at the 4th and 5th branch orders of basket cells of *Vgat* ^{ECKO} group (n=9, *P<0.01, ANOVA and post-hoc tests). (D) Comparison of dendritic length x 30-um interval from the soma of basket cells between *Vgat* $^{fl/fl}$ and *Vgat* ^{ECKO} somatosensory cortex. The results showed a significant reduction in dendritic lengths at a distance of 90 to 120-um from the soma of basket cells of *Vgat* ^{ECKO} group (n=9, *P<0.01, ANOVA and post-hoc tests).
