# Supplementary material for: Endothelial cell-derived GABA signaling modulates neuronal migration and postnatal behavior
Source: Cell Res. 2017 Oct 31;28(2):221–48. doi: 10.1038/cr.2017.135 (PMC5799810; doi:10.1038/cr.2017.135)
Supplement: Supplementary information, Table S1 — List of epilepsy related genes that were altered in VgatECKO telencephalon [file cr2017135x16.pdf]

Table 1: List of epilepsy related genes that were altered in *Vgat* ^{ECKO} telencephalon

Mctague 2015 Syndromic Cases Gene Table:

Syndromic cases	Gene Symbol
Dravet syndrome	GABRG2, STXBP1, HCN1, PCDH19, GABRA1, SCN1A
Early infantile epileptic encephalopathy	GNAO1, STXBP1, CACNA2D2, PIGQ, NECAP1, SLC25A22, ARX, AARS, KCNA2, SIK1, KCNT1, SCN8A, BRAT1, PIGA
Early myoclonic encephalopathy	ERBB4, SLC25A22, SIK1, SETBP1, PIGA
Epilepsy of infancy with migrating focal seizures	SLC12A5, QARS, SLC25A22, PLCB1, KCNT1, SCN8A, TBC1D24, SCN1A
Epilepsy with myoclonic atonic seizures	SLC6A1, GABRG2, SLC2A1, KCNA2, SCN1B, CHD2, GABRA1, SCN1A
Epilepsy-aphasia spectrum	GRIN2A
Lennox-Gastaut syndrome	HNRNPH1, GABRB3, STXBP1, DNM1, GRIN2B, KCNQ3, CDKL5, FLNA, CACNA1A, CHD2, IQSEC1, MTOR, SCN8A, ALG13, SCN1A
West syndrome	PTEN, GNAO1, GABRB3, SPTAN1, TCF4, NRXN1, MEF2C, STXBP1, DNM1, DOCK7, GRIN2B, SLC25A22, ARX, PLCB1, CDKL5, FOXG1, SIK1, MAGI2, GRIN1, GABRB1, SETBP1, ALG13, NDP, TBC1, D24, SCN1A, SLC35A2, WWOX, PIGA, GRIN2A

CDT Selected Conditions with Direct Evidence Indications

Disease Name	Gene Symbol
Amish Infantile Epilepsy Syndrome	ST3GAL5
Borjeson-Forssman-Lehmann syndrome	PHF6
Cortical Dysplasia-Focal Epilepsy Syndrome	CNTNAP2
Epilepsies, Myoclonic	GABRG2, PMP22 , SCN1A, STXBP1
Epilepsies, Partial	KCNT1
Epilepsy	DRA2A, AKT1, AUTS2, BDNF, CHD2, CHRM1, CHRM2, CNTNAP2, ERN1, FLNA, FOLR1, GABRB3, GFAP GPX1, GRIN2A, GRIN2B, GRM1, HCN1, ILK, KCNAB2, KCNH1, KCNQ2, L2HGDH, MEF2C, NPY, NPY2R, OPRM1, P2RX2, P2RX4, PCDH19, POLG, RAB39B, SCN1A, SCN8A, SLC1A1, SLC1A2, SLC1A3, SLC4A10, SLC12A2, SLC12A5, SPARCL1, STAMBP, STX1B, SYNGAP1, TSC1, TSC2, TXNRD1, UBE3A, VDAC1, VDAC2
Epilepsy, Absence	CACNA1A, CACNA1H, CACNA2D2, CLCN2, EFHC1, GABRB3, GABRG2, JRK, KCNK9
Epilepsy, Benign Neonatal	KCNQ2
Epilepsy, Complex Partial	SCN3A

Epilepsy, Familial Temporal Lobe, 1	LGI1
Epilepsy, Familial Temporal Lobe, 4	ETL4
Epilepsy, Female-Restricted, Mental Retardation	PCDH19
Epilepsy, Frontal Lobe	CHRNA4, CHRNB2, KCNT1, SLC4A10
Epilepsy, Generalized	CACNB4, CHRNA7, JRK, SCN1A
Epilepsy, Idiopathic Generalized	CACNB4, CLCN2, GABRD, ME2, OPRM1
Epilepsy, Nocturnal Frontal Lobe, Type 1	CHRNA4
Epilepsy, Nocturnal Frontal Lobe, Type 3	CHRNB2
Epilepsy, Partial, with Variable Foci	DEPDC5
Epilepsy, Progressive myoclonic, 3	KCTD7
Epilepsy, Rolandic	GRIN2A
Epilepsy, Temporal Lobe	CNR1, GRM1, GRM2, GRM3, GRM4, GRM5, KCNC4, KDR, NPY, NPY2R, P2RX4, P2RX7, SLC1A1, SLC12A2, SLC12A5, SLIT2, TEK, VEGFA
Epilepsy, Tonic-Clonic	BCHE, BDNF, HBEGF, NES, NGF, NTF3, OXT
Epilepsy, X-Linked, with Learning Disabilities	SYN1
Epileptic Encephalopathy, Early Infantile	ARX, CDKL5, HCN1, CDKL5, SLC25A22, STXBP1, SPTAN1, KCNQ2, PLCB1
Epileptic encephalopathy, Lennox-Gastaut type	MAPK10
Familial encephalopathy	SERPINI1
Focal cortical dysplasia of Taylor	TSC1
Generalized Epilepsy and Paroxysmal Dyskinesia	KCNMA1
Generalized Epilepsy, Febrile Seizures	GABRD, GABRG2, SCN1A, SCN1B, SCN1A, SCN9A, GABRG2
Hyperekplexia and Epilepsy	ARHGEF9
Hypoparathyroidism-retardation-dysmorphism	TBCE
Lafora Disease	EPM2A, NHLRC1
Landau-Kleffner Syndrome	GRIN2A
Mental Retardation, Autosomal Dominant 20	MEF2C
Mental retardation, X-linked, syndromic 5	AP1S2
Mental Retardation, X-Linked, Syndromic	SLC9A6
Mental Retardation, X-Linked, with Epilepsy	ATP6AP2
Microcephaly, Postnatal Progressive, with seizures and brain atrophy	MED17
Microcephaly, Seizures and developmental delay	PNKP
Myoclonic Epilepsies, Progressive	AFG3L2, ATN1, CLN6, EPM2A, KCNC1, NEU1, NHLRC1, PRNP, SACS, SERPINI1, TBC1D24, TSC1
Myoclonic Epilepsy, Familial Infantile	TBC1D24
Myoclonic Epilepsy, Juvenile	CACNB4, CLCN2, EFHC1, GABRA1, JRK
Norrie disease	NDP
Pachygyria with mental retardation, seizures	WDR62
Partington X-linked mental retardation syndrome	ARX
Phosphoserine Aminotransferase Deficiency	PSAT1

Megalencephaly, And Symptomatic Epilepsy	STRADA
Prickle1-Related Progressive Myoclonic Epilepsy with Ataxia	PRICKLE1
Pyridoxamine 5-Prime-Phosphate Oxidase Deficiency	PNPO
Pyridoxine-dependent epilepsy	ALDH7A1
Seizures	ABAT, ABCB1A, ACAT1, ACHE, ADORA2A, ADRA1B, AGT, ALAD, ALPL, APEX1, APOE, ATP7A, BCHE BDNF, CAT, CCK, CDKL5, CHRM1, CHRNA3, CHRNA4, CHRNA5, CHRNA7, CHRNB4, CLU, CNNM2 CNR1, DRD2, FAAH, FOXG1, GABRA5, GAD2, GAMT, GRIK1, HCN1, HTR1A, HTR1B, IMPA1, KCNA2, KCNJ11, LETM1, MECP2, MIB1, MPDZ, MT3, NGF, NGFR, NOS1, NOS2, NPY, OPRK1, OPRM1, OXT, PAM, PDYN, PIGM, PNKP, POLG, PRKCD, RBFOX1, REST, SIGMAR1, SLC1A1, SLC2A1, SLC6A1, SLC8A1, SLC8A3, SLC12A5, SLC17A7, SLC30A1, SOD2, SST, SSTR2, SYN2, TCF4, TRH, TSC2
Seizures, Benign Familial Neonatal	KCNQ2, KCNQ3
Seizures, Febrile	ANO3, GABRG2, IMPA2, SCN1A, SCN1B, STX1B,
SeSAME syndrome	KCNJ10
Spasms, Infantile	HSD17B4, TSC1, TSC2
Status Epilepticus	ABCB1A, ANK3, AQP4, ATP2A2, BDNF, BECN1, CASP3, CASP8, CAT, CCL2, CCL3, CCR7, CDH2, CNR1, DMD, EIF2AK2, EIF2AK3, EIF2S1, GAP43, GRIA2, GRM1, GRM5, HMOX1, IL1RN, JUN, JUNB, JUND, KCNMA1, LAMP2, MEF2C, NGF, NOS1, NOS2, NTF3, NTRK2, NTRK3, PDXK, PNPO, PTK2B, RET, SCN8A, SLC8A1, SLC8A3, SLC12A5, SNTA1, SRC, SSTR1, SSTR2, SSTR4, VEGFA
Unverricht-Lundborg Syndrome	CSTB
